# Supramolecular packing of alkyl substituted Janus face all-*cis* 2,3,4,5,6-pentafluorocyclohexyl motifs†

**DOI:** 10.1039/d1sc02130c

**Published:** 2021-06-04

**Authors:** Joshua L. Clark, Alaric Taylor, Ailsa Geddis, Rifahath M. Neyyappadath, Bruno A. Piscelli, Cihang Yu, David B. Cordes, Alexandra M. Z. Slawin, Rodrigo A. Cormanich, Stefan Guldin, David O'Hagan

**Affiliations:** School of Chemistry, University of St Andrews North Haugh, St Andrews, Fife KY16 9ST UK do1@st-andrews.ac.uk; Department of Chemical Engineering, University College London Torrington Place London WC1E 7JE UK; Chemistry Institute, University of Campinas Monteiro Lobato Street, Campinas Sao Paulo 13083-862 Brazil

## Abstract

This study uses X-ray crystallography, theory and Langmuir isotherm analysis to explore the conformations and molecular packing of alkyl all-*cis* 2,3,4,5,6-pentafluorocyclohexyl motifs, which are prepared by direct aryl hydrogenations from alkyl- or vinyl-pentafluoroaryl benzenes. Favoured conformations retain the more polar triaxial C–F bond arrangement of the all-*cis* 2,3,4,5,6-pentafluorocyclohexyl ring systems with the alkyl substituent adopting an equatorial orientation, and accommodating strong supramolecular interactions between rings. Langmuir isotherm analysis on a water subphase of a long chain fatty acid and alcohol carrying terminal all-*cis* 2,3,4,5,6-pentafluorocyclohexyl rings do not show any indication of monolayer assembly relative to their cyclohexane analogues, instead the molecules appear to aggregate and form higher molecular assemblies prior to compression. The study indicates the power and potential of this ring system as a motif for ordering supramolecular assembly.

## Abstract 2

Theory and Langmuir isotherm analysis was used to explore the conformations and molecular packing of alkyl all-*cis* 2,3,4,5,6-pentafluorocyclohexyl motifs, which are prepared by direct aryl hydrogenations from alkyl- or vinyl-pentafluoroaryl benzenes.

## Graphical abstract

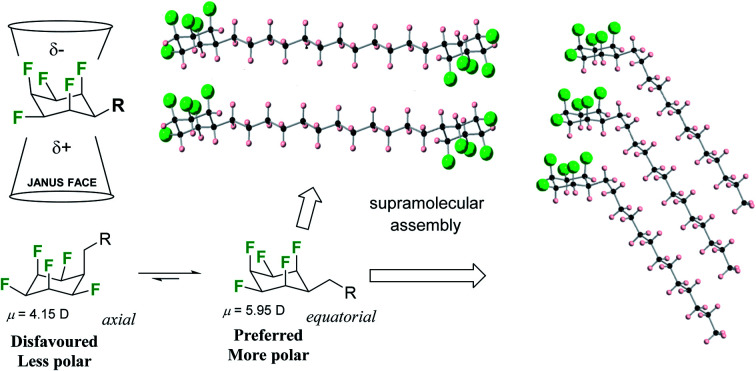

## Introduction

**Fig. 1:**
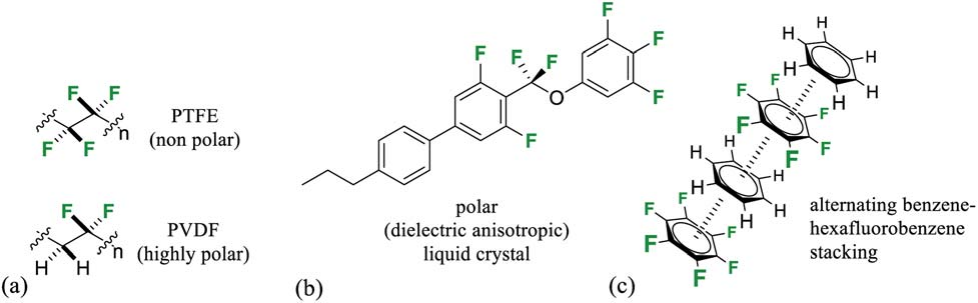
(a) Minimal structures of (PTFE) and polar (PVDF) organofluorine polymers; (b) selectively fluorinated −ve dieletric anisotropic liquid crystal;^6*a*^ (c) representation of benzene–hexafluorobenzene stacking in the solid state.^11,12^

Organofluorine compounds have long made important contributions to organic materials chemistry and society more generally.^1^ Perfluorocarbons where all of the hydrogens in an aliphatic material are replaced by fluorines display very high heat stabilities and are known for their chemical stability as well as their immiscibility with both hydrocarbons and water and ability to dissolve gases.^2^ The ‘fluorous phase’^3^ has been coined to recognise the unique properties of this class of materials which is exemplified most prominently by the ‘non-stick’ polymer and coating poly(tetrafluoroethylene) (PTFE).^4^ Partially fluorinated materials have very different properties and are characterised by an increasing polarity relative to fluorocarbons or hydrocarbons. Selective fluorination will induce polarity, and a polymer such as poly-(vinylidine fluoride)-PVDF displays piezoelectric properties due to dipoles created by the alternating –CF_2_– and –CH_2_– groups, a property maximised by poling (Fig. 1a).^5^ Similarly there are a range of liquid crystalline materials which are used in modern displays and which derive their performance due to an orientated polarity introduced by selective fluorination of an organic material (Fig. 1b).^6^

**Fig. 2:**
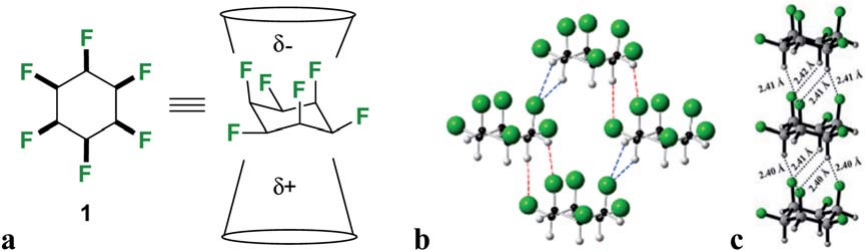
Hexafluorocyclohexane **1** (a) structure and non-equivalent facial polarity profile; (b) X-ray structure of the prototype Janus face all-*cis* hexafluorocyclohexane **1**; (c) theoretically predicted optimal molecular packing of **1** differs from the observed X-ray derived structure.^11^

We have developed an interest in the synthesis and properties of selectively fluorinated cyclohexanes and find that partial fluorination of aliphatic rings can lead to highly polar aliphatic motifs, particularly if isomers are selected that place fluorines on the same side of the cyclohexane ring.^7^ All-*syn*-1,2,3,4,5,6-hexafluorocyclohexane **1** shown in Fig. 2a is the prototype compound of this class.^8^ Cyclohexane **1** has all of its six fluorines on one face of the ring and is considered to be among the most polar aliphatic compounds known.^9^ The molecule decomposes/sublimes at 208 °C, extraordinarily high for a low molecular weight aliphatic, and it has a molecular dipole moment of 6.2 D [calculated at M11/6-311G(2d,p) level], again extraordinarily high for an aliphatic. The polarity arises because there are three axial C–F bonds in the cyclohexane ring which are co-aligned and which generate a strong orientated dipole due to the maximal electronegativity of fluorine. The hydrogens around the ring, three of them co-axial, become polarized by the geminal fluorine atoms rendering them electropositive.^7*b*^ Accordingly the molecule has an electrostatically biased negative (fluorine) face and an electrostatically biased positive (hydrogen) face. More generally aromatic rings are electrostatically negative on both faces and cycloalkanes are neutral and hydrophobic, however there is no obvious counterpart ring in organic chemistry that has a −ve and a +ve face. This polarised aspect has led to **1** being described as a ‘Janus face’ ring system,^10^ and it can be envisaged that electrostatic attraction between the fluorine and hydrogen faces of such ring systems could play a role as a motif for ordering and stabilising supramolecular assembly. Perhaps the closest related phenomenon to that described for these Janus face cyclohexanes is the widely celebrated outcome, described in the early 1960's by Patrick and Prosser, when benzene and hexafluorobenzene are mixed equally (Fig. 1c).^11^ The two liquid components generate a solid material (mp 24 °C) which has stacked and alternating (offset) aryl- and hexafluoroaryl-rings, an arrangement accommodated by the complementary electrostatic profiles of these ring systems. For benzene the ring core is electronegative and the peripheral hydrogens electropositive, whereas for the hexafluorobenzene the ring core is electropositive and the peripheral fluorines are electronegative. This ordering opened up a wide area of supramolecular exploration which remains active until the present.^12^ Janus rings of **1** can be considered to approximate a fusion of benzene and hexafluorobenzene, with the ability to display a similar ordering phenomenon within a single ring system. When rings of **1** associate, they order electrostatically with the fluorine face of one ring contacting the hydrogen face of another (Fig. 2b). Theoretical studies indicate that the interaction energy of two isolated rings of **1** is ∼8.2 kcal mol^−1^,^13^ about the strength or stronger than a hydrogen bond, an energy that will be substantial in supramolecular ordering. The crystal structure of **1** (Fig. 2b) has the rings arranged such that stacking is offset from the perpendicular, however theoretical studies indicate that perpendicular packing shown in Fig. 2c should be optimal and that there is a relatively low energy between these polymorphic arrangements. In this paper we explore supramolecular ordering of mono-alkyl substituted derivatives of cyclohexane **1**.

**Fig. 3:**
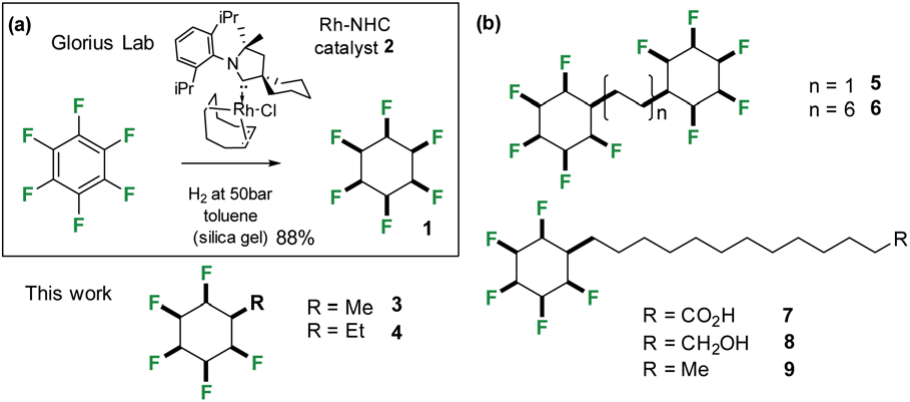
(a) Aryl hydrogenation developed by Glorius^14*b*^ was adapted to the synthesis of alkyl substituted all-*cis* 1,2,3,4,5-pentafluorocyclohexane rings systems; (b) structures of alkyl substituted all-*cis* pentafluorocyclohexanes **3–9** prepared in this study.

**Fig. 4:**
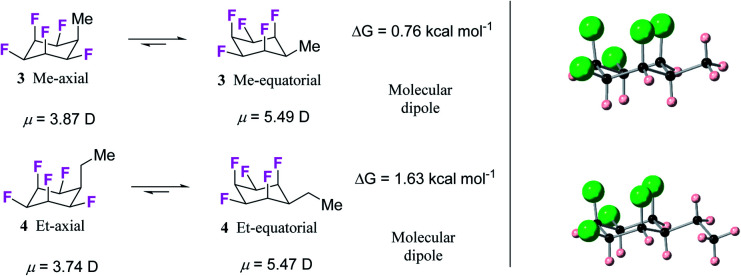
(a) Calculated (M06L-D3/aug-cc-pVTZ level) equilibrium energies between axial/equatorial conformers of alkyl substituted all-*cis* 1,2,3,4,5,-pentafluorocyclohexanes **3** and **4**; (b) solid state X-ray structures of **3** (upper) and **4** (lower). In each case the equatorial conformer is favoured.

A clear challenge in order to explore the properties of these Janus face systems is access to an appropriate syntheses to prepare such compounds. Recent and important progress has been made by the Glorius laboratory,^14^ who have demonstrated that multiply-fluorinated aromatics can be directly hydrogenated to generate the corresponding all-*cis* fluorocyclohexane products in one step, without any significant loss of fluorine. The method used the cyclic(alkyl)(amino)carbene (CAAC)/Rh catalyst **2** developed by Zeng^15^ for aryl hydrogenations. Glorius demonstrated that cyclohexane **1** could be efficiently prepared from hexafluorobenzene by this approach as illustrated in Fig. 3a reducing the original twelve step protocol to one step. It follows that a range of monosubstituted alkyl derivatives of cyclohexane **1** could now be prepared by direct hydrogenation of alkyl substituted pentafluorobenzenes, an approach that forms the focus of this paper. Indeed, we find that both the R = Me **3** and R = Et **4** (Fig. 4) substituents are accessible directly by hydrogenation of their pentafluoroaryl precursors, as the first examples of alkyl all-*cis* 1,2,3,4,5-pentafluorocyclohexyl derivatives. Developing this further, we also report the synthesis and some properties the bis-cyclohexyl tethered rings **5** and **6** with short and long alkyl spacers between the rings and then systems **7–9**, with longer alkyl chains terminating in either alkyl, alcohol or carboxylic acid residues. These compounds are illustrated in Fig. 3b. In order to gain insight into supramolecular assembly we have determined the solid state structures of the **3** and **4**, bis-ring systems **5** and **6**, as well as **8** and **9** and we have investigated the deposition of the long chain alkyls **7–9** on a water subphase. The outcomes are reported below.

## Results and discussion

Hydrogenation (50 bar H_2_) of pentafluoroaryl-toluene and pentafluoroaryl-ethylbenzene using catalyst **2** generated the corresponding methyl- and ethyl-functionalised all-*cis* pentafluorocyclohexanes **3** (55% yield) and **4** (76% yield) respectively (Fig. 4). Cyclohexanes **3** (mp = 182 °C) and **4** (mp = 148 °C) have high melting points consistent with strong electrostatic attraction between the rings, and this contrasts with their hydrocarbon or perfluorocarbon analogues which are liquids at ambient temperature. It was of immediate interest in the context of supramolecular assembly to establish if the alkyl substituents preferred an axial over an equatorial orientation as it was not obvious if the steric effect of an axial Me/Et group and two axial C–F bonds would be more or less stabilising than the electrostatic repulsion associated with three axial C–F bonds when the Me/Et group is equatorial (Fig. 4a).

Since both **3** and **4** are crystalline solids their crystal structures were determined by X-ray analyses and are illustrated in Fig. 4b. In each case the solid-state structures have the alkyl groups equatorial with triaxial C–F bonds. To gain a deeper insight into the favoured conformations of these mono alkylated cyclohexyl systems a theory study (M06L-D3/aug-cc-pVTZ level) was carried out to establish the relative energies between the axial and equatorial conformers of **3** and **4** in the gas phase.^16^ This theory level showed the best results in benchmarking calculations using the DLPNO-CCSD(T)/def2-TZVP level as the benchmark (see ESI†). The results are summarised in Fig. 4a. For cyclohexane **3** it emerged that the equatorial Me conformer is of lower energy despite it having a significantly higher molecular dipole (**3**_ax_ = 3.87 D *versus***3**_eq_ = 5.49 D), although the energy difference between conformers is not large (Δ*G* = 0.76 kcal mol^−1^), tending towards an isoenergetic situation, and being significantly less than for example methylcyclohexane (∼1.74 kcal mol^−1^).^17^ However the equatorial preference increases significantly (Δ*G* = 1.63 kcal mol^−1^) for ethylcyclohexane **4** although the situation is a little more complex due to ethyl group rotation. The equilibrium energy difference was calculated after rotational energy profiles (see ESI†) established the minimum energies for the axial and equatorial conformers of **4**. The conformations in the solid-state structures of **3** and **4** are clearly equatorial, reinforced by the strong intermolecular association between the more polar conformers in the solid state, and this augers well for the reliability of supramolecular packing for this series (alkyl all-*cis* 1,2,3,4,5-pentafluorocyclohexane motifs) more generally.

**Fig. 5:**
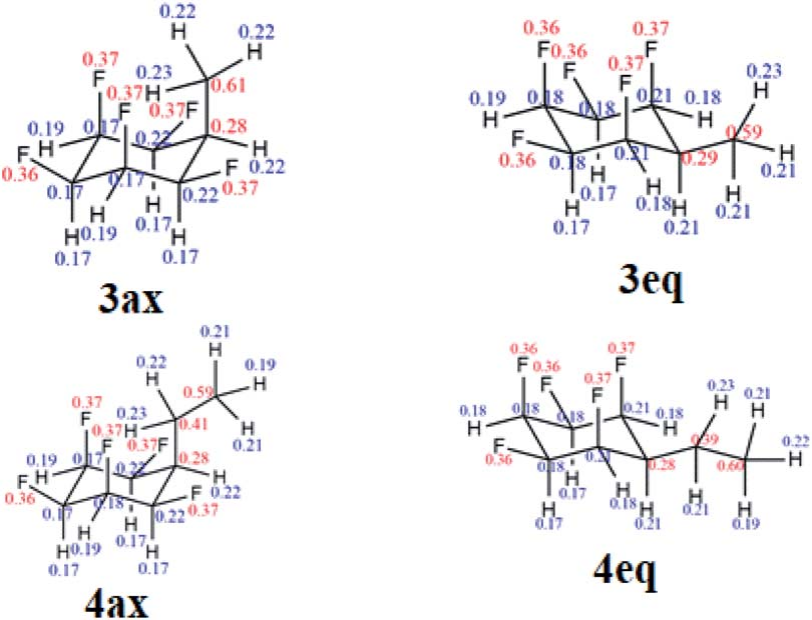
Calculated NPA charges for the axial and equatorial conformers of **3** and **4** at the M06-2X/aug-cc-pVTZ level. Blue and red represent positive and negative charges, respectively (in au).

Natural bond orbital (NBO) analysis^16,18^ was carried out to rationalise the significantly larger equatorial preference for **4** over **3** (Table S1 in the ESI†). NBO analysis separates the relative global electronic energy [Δ*E*(T)] into its Natural Lewis energy [Δ*E*(L)], which accounts for a chemical structure without any delocalisation and therefore representing steric and electrostatic contributions only, from Natural Non-Lewis energies [Δ*E*(NL)] which account for hyperconjugation. NBO analysis indicates that the increase in energy difference between the ax/eq conformers of **4** relative to **3** is due to a balance between electrostatics Δ*E*(NCE) as calculated from the Natural Coulomb Electrostatic (NCE) analysis,^16*b*^ and hyperconjugation Δ*E*(NL), with electrostatics favouring the axial conformer (−0.95 kcal mol^−1^) and global hyperconjugation interactions favouring the equatorial conformer in **3** (1.17 kcal mol^−1^) (Table S1 in ESI†). On the other hand, the Et derivative **4** has a higher preference for the equatorial conformer, because now the electrostatic term [Δ*E*(L)] (0.68 kcal mol^−1^) also favours the equatorial conformer. This arises from replacing a positively charged H atom in **3** by a CH_3_ group with a negatively charged C atom in **4** (Fig. 5), the interactions with the axial F atoms become destabilising (see Tables S2 and S3 in the ESI† for individual atom–atom interactions) and disfavour the axial geometry to a greater extent (Δ*G* = 1.63 kcal mol^−1^).

Another interesting feature of the NBO analysis is the possibility to decompose the total molecular dipole moment quantitatively into the individual bonds and lone pairs that sum to give the overall value.^16^ Since the total dipole moment of compounds **1**, **3** and **4** is mainly directed from the hydrogen to the fluorine face of the cyclohexane ring, we analysed the bonds and lone pairs that contribute to this axis (Table S5 in the ESI†). Accordingly, each C–F_ax_ bond contributes ∼1.8 D, and the F_ax_ lone pairs ∼1.4 D, to the total dipole, whereas the C–F_eq_ bonds and F_eq_ lone pairs contribute only ∼0.5 D and 0.4 D, respectively. Thus, by replacing an equatorial C–F_eq_ bond from **1** by an alkyl group as in **3** and **4**, only a small decrease in the total dipole moment occurs, and therefore the mono substituted ring retains high polarity.

**Fig. 6:**
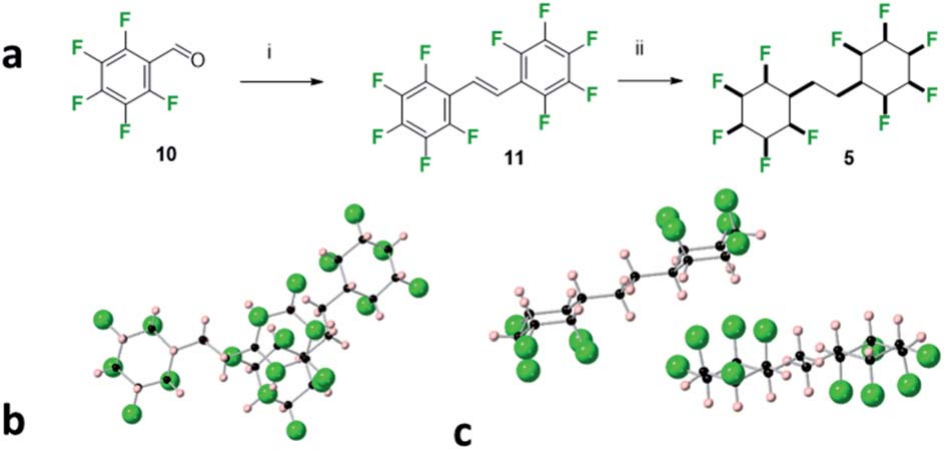
(a) Synthesis of **5**. (i) Zn, TiCl_4_, THF, 70 °C, 4 h, 19% yield; (ii) 50 bar H_2_ gas, 1 mol% cat (**2**), 4 Å MS, hexane, 24 h, 28% yield. (b) X-ray crystal structure showing two molecules of 1,2-bis-(all-*cis*-2,3,4,5,6-pentafluorocyclohexyl)ethane **5** with one molecule lying above the other and (c) with an orientation highlighting an interdigitated packing.

**Fig. 7:**
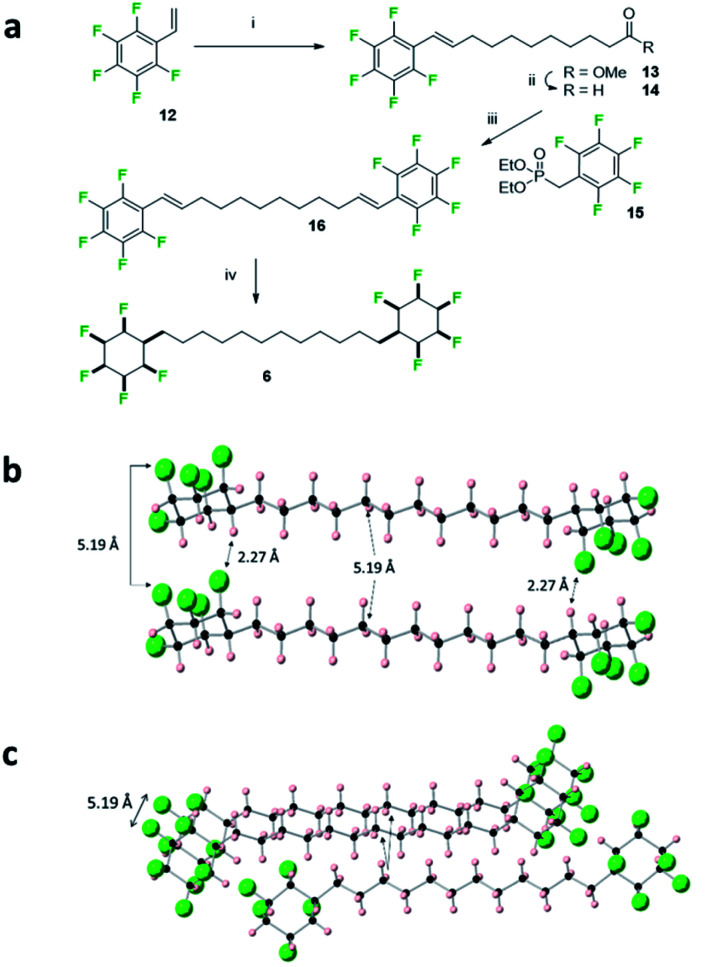
(a) Synthesis route to **6** involving the exhaustive hydrogenation of **16**. (i) Methyl 10-undecenoate 2nd generation Hoveyda–Grubbs catalyst, CH_2_Cl_2_, 40 °C, 14 h, 54%; (ii) DIBAlH, CH_2_Cl_2_, −78 °C, 1 h, quantitative; (iii) NaH, THF, 0 °C then **14**, 0 °C to 66 °C, 53%; (iv) H_2_ (50 bar), **2** (5 mol%), 4 Å MS, hexane, r.t., 33%. (b) X-ray crystal structure showing the alignment of adjacent molecules of **6**. Short CF⋯HC contacts (2.27 Å) are illustrated which suggest a strong electrostatic interaction between the cyclohexane rings. (c) View incorporating a third molecule of an adjacent stack illustrating the condensed packing of the alkyl chains.

The bis-cyclohexane systems **5** and **6** were next prepared, with two all-*cis* 1,2,3,4,5-pentafluorocyclohexyl rings anchored between a shorter and a longer aliphatic spacer. The synthesis routes to **5** and **6** are summarised in Fig. 6 and 7. Compound **5** was prepared by direct hydrogenation of decafluorostilbene **11**, a known substrate readily accessible by a McMurry coupling of pentafluorobenzaldehyde **10**.^19^ The hydrogenation reaction (catalyst **2**, 50 bar H_2_) proved sluggish and product **5** was notably highly insoluble in standard chromatography solvents. As a consequence, a suitable sample for crystal structure analysis was isolated by recrystallisation rather than column chromatography. It was notable that bis-cyclohexane **5** did not have a classical melting profile; showing discolouration at 210 °C and remaining unmelted at 300 °C! This unusual behaviour for a relatively low molecular weight aliphatic is similar to that observed for cyclohexane **1** (mp = 208 °C),^8^ and it is indicative of the strong electrostatic interactions between the rings in the solid state. A suitable crystal of **5** was selected for crystal structure analysis and views of the resultant structure are shown in Fig. 6.

In the solid state, the linking alkyl chain lies equatorial and molecules of **5** pack in an interdigitated manner rather than one on top of each other. The rings of adjacent molecules associate, with the complementary fluorine and hydrogen faces contacting each other. When two molecules are viewed one above the other (Fig. 6a) an axial fluorine of one ring is pointing directly towards the axial hydrogens of the next ring. The three CF⋯HC contact distances are almost equidistant, (2.46 Å, 2.46 Å & 2.54 Å) and below the van der Waals contact distance (2.67 Å)^20^ for hydrogen and fluorine. The orientation of the peripheral cyclohexyl rings in any molecule of **5** is antiparallel, consistent with an arrangement which reduces the molecular dipole of an isolated molecule and reduces the net polarity of the crystal.

The bis-cyclohexyl system **6** with a longer linker was prepared as illustrated in Fig. 7 after a metathesis reaction^21^ between styrene **12** and methyl undec-1-enoate, to generate ester **13**. Selective reduction gave aldehyde **14** which was amenable to a Horner–Wadsworth–Emmons olefination^22^ with phosphonate **15** to generate bis-olefin **16**. Exhaustive hydrogenation of both the double bonds and the aryl rings with catalyst **2** generated the desired product **6**, again as a solid material. The melting point of **6** (188 °C) was lower than that of **5** presumably on account of the longer aliphatic spacer.

In the solid state, the structure shows simpler packing than **5**, with the peripheral rings in **6** stacked directly one above another along the *b*-axis, and where the linking chains align, spaced by a repeat distance of 5.19 Å for both the cyclohexane rings and the aliphatic chains, slightly longer than the typical (4.5–5.0 Å) distances for optimal packing in hydrocarbon chain assemblies.^23^ There are three CF⋯HC contacts within stacks of molecules, two at 2.39 Å and 2.46 Å, and one notably short at a CF⋯HC distance of 2.27 Å. Such distances are among the shortest CF⋯HC contacts found in the Cambridge Structural Database^24^ and well below the sum of the van der Waal's F⋯H contact distance (2.67 Å)^20^ suggesting that the electrostatic attraction between the fluorine and hydrogen faces of the rings is leading to a compression and accommodating these non-classical hydrogen bonds. As well as these contacts within stacks, adjacent stacks are also linked by CF⋯HC contacts, at 2.32 Å.

The study was then extended to the synthesis of the long chain fatty acid **7** with a terminal pentafluorocyclohexane ring system. The classical amphiphilic nature of fatty acids is such that they adopt organised supramolecular assemblies in the solid state or on the surface of water supported by hydrogen bonding of the carboxylate head groups and van der Waals interactions of the aliphatic chains, typical of lipid membranes.^25^ In the solid state such arrangements can be interdigitated to form a repeat monolayer or the molecules can arrange head to head and form bilayers. On water, the carboxylate head groups associate with the aqueous subphase and chains aggregate into monolayer or bilayer assemblies, a process that can be monitored in a Langmuir trough through pressure area isotherm analysis.^26^ Therefore long chain fatty acid **7** was targeted as a molecular tool in which to further explore the mode of supramolecular assembly of the all-*syn* pentafluorocyclohexyl motif. The study extended to preparing the corresponding alcohol **8** and methyl terminated alkane **9**. The analogous long chain fatty acid **21** and alcohol **22**, without any fluorines, were also prepared as reference compounds for Langmuir isotherm analyses.

**Scheme 1:**
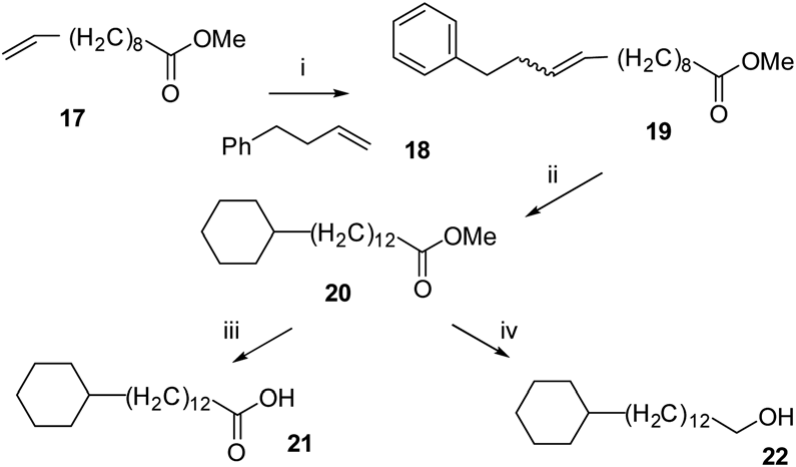
Synthetic routes to non-fluorinated cyclohexyl fatty acid **12** and long chain alcohol **13**. (i) 4-phenylbutene **18**, Grubbs first generation catalyst ®, CH_2_Cl_2_, 40 °C, 14 h, 39%; (ii) H_2_ (50 bar), **2** (8 mol%), 4 Å MS, hexane, r.t., 74%; (iii) NaOH, H_2_O, MeOH, 65 °C; (iv) DIBALH, CH_2_Cl_2_, −78 °C to r.t., 1 h, 81%.

**Scheme 2:**
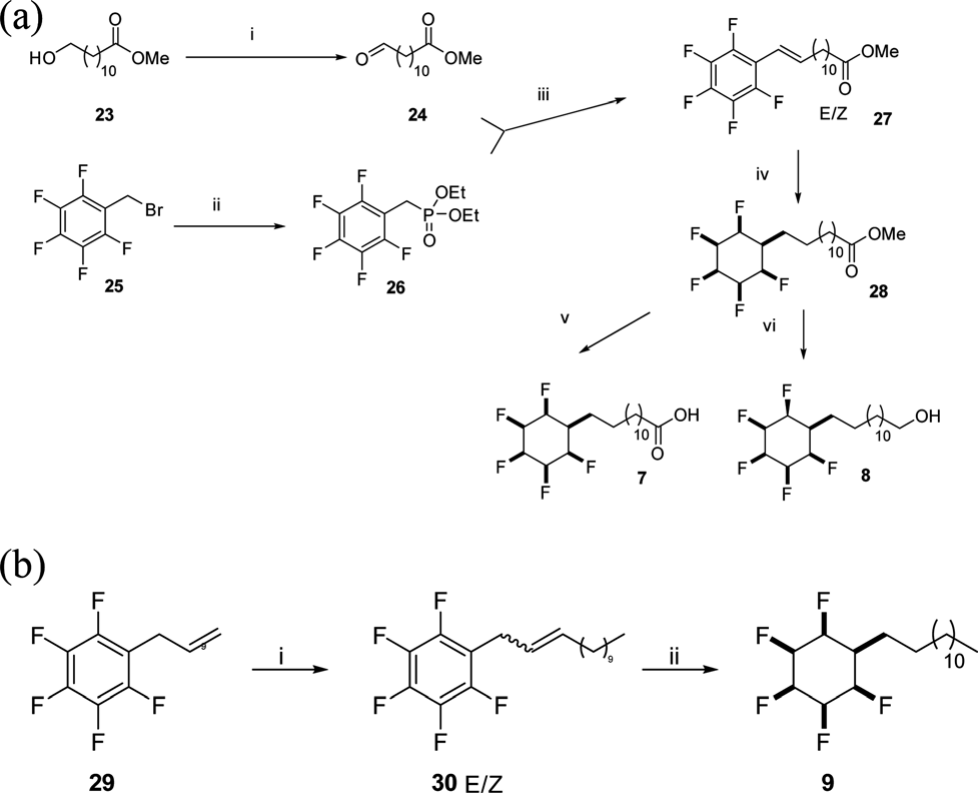
(a) Synthetic routes to all-*cis* pentafluorocyclohexyl fatty acid **7** and alcohol **8**. (i) (COCl)_2_, DMSO, Et_3_N, CH_2_Cl_2_, −78 °C, 74%; (ii) P(OEt)_3_, microwave, 140 °C, 5 min, 99%; (iii) NaH, THF, 0 °C to 50 °C, 14 h, 58%; (iv) **2** (3 mol%), H_2_ (50 bar), silica gel, hexane, r.t., 22%; (v) HCl (6 N), H_2_O, 100 °C, 14 h, 95%; (vi) DIBAlH, CH_2_Cl_2_, −78 °C to r.t., 64%. (b) Synthetic route to all-*cis* pentafluorocyclohexyl long chain aliphatic **9**. (i) Grubbs first generation catalyst, 1-dodecene, CH_2_Cl_2_, 40 °C, 14 h; (ii) H_2_ (50 bar), **2** (3 mol%), 4 Å MS, hexane, r.t., 32% over two steps.

The synthesis routes to fatty acid **21** and long chain alcohol **22** are illustrated in Scheme 1. The length of the chain was assembled by fusing together methyl 10-undecenoate **17** and phenylbutene **18** in a cross-metathesis protocol.^21^ This proved to be relatively straightforward and the resultant ester **19** was subject to an exhaustive aryl/olefin hydrogenation reaction with catalyst **2**. The product aliphatic ester **20** was either progressed by reduction to generate alcohol **22** or it was hydrolysed to the desired fatty acid **21**. The synthesis of carboxylic acid **7** and alcohol **8** was accomplished as illustrated in Scheme 2. This involved a Horner–Wadsworth–Emmons (HWE) olefination^23^ between pentafluoroaryl phosphonate **26** and long chain ester-aldehyde **24** to generate ester **27** as a mixture of *E* and *Z* stereoisomers. Direct aryl hydrogenation under pressure resulted in the formation of the saturated ester **28**. Ester **28** was then hydrolysed to generate fatty acid **7** and was separately reduced to long chain alcohol **8**. In a separate sequence, a cross metathesis reaction between terminal alkene **29** and 1-dodecene to generate olefin **30** generated the resultant olefin **30** which was also submitted to an exhaustive hydrogenation reaction with catalyst **2** (ref. 15) to generate alkylcyclohexane **9**. Melting point comparisons where made between the analogous partially fluorinated and non-fluorinated products as an immediate comparator of the anticipated difference in physical properties between the two series. These are summarised in Table 1.

**Table 1:** Melting point comparisons between cyclohexyl and all-*cis* 2,3,4,5,6-pentafluorocyclohexyl systems

	Pentafluoro	Hydrocarbon
Fatty acids	**7** = 178 °C	**21** = 65 °C
Long chain alcohols	**8** = 140 °C	**22** = 34 °C
Tridecylcyclohexane	**9** = 121 °C	14 °C^a^

a Ref. 25.

It is clear that the melting points are very much higher for the fluorinated analogues consistent with electrostatic attraction between the faces of the all-*cis*-2,3,4,5,6-pentafluorocyclohexyl rings in the solid state.

**Fig. 8:**
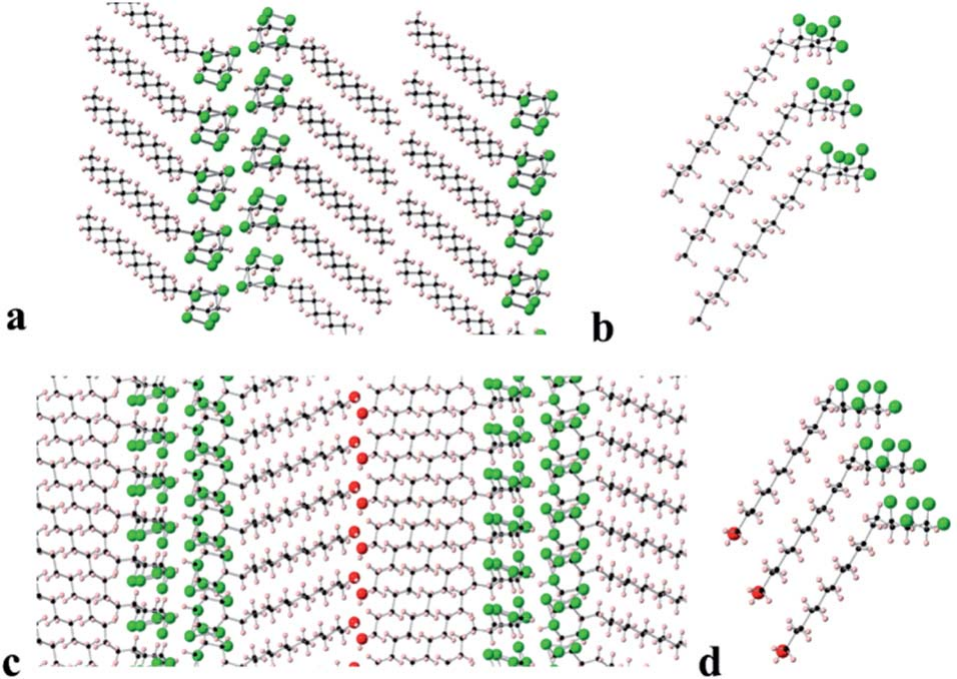
Views of the X-ray determined crystal structures of tridecyl alkane **9** (upper) and long chain alcohol **8** (lower). (a) Cross sectional view down the *b*-axis of the packing arrangement of tridecyl alkane **9** and (b) the stacking arrangement of three molecules of **9** showing an angled (130°) arrangement between the alkyl chains and the cyclohexyl rings. (c) Cross sectional view down the (1 −1 0) axis of the packing arrangement of long chain alcohol **8** and (d) the stacking arrangement of three molecules of **8** also showing an angled (120°) arrangement optimised for intermolecular contacts between the alkyl chains and cyclohexyl rings.

Long chain alkane **9** and alcohol **8** generated suitable crystals for X-ray crystal structure analysis. The resultant packing structures are shown in Fig. 8.

Compound **9** (Fig. 8a and b) adopts a packing structure similar to that found for hexafluorocyclohexane **1** where there is an offset between adjacent cyclohexane rings. The equivalent ring atoms (fluorines or hydrogens) are 5.62 Å apart, which is an increase on the equivalent spacing found in **6** (5.19 Å). The long chain alkane moiety extends linearly in a classical extended anti zig-zag conformation and the terminal methyl groups interface to form a double lamellar (bilayer) hydrophobic domain. The aliphatic chains are compact running parallel to each other at a regular distance of 4.0–4.2 Å along the chains. To accommodate this narrowing relative to the cyclohexane ring spacing (5.62 Å) and to presumably maximise van der Waals interactions, the chains are orientated at an angle 130° with respect to the plane of the cyclohexane rings.

In the case of long chain alcohol **8** (Fig. 8c and d), the molecules arrange in a similar manner but there is an additional extended hydrogen bonding network, forming chains running along the *a*-axis between the terminal alcohol moieties of two molecular domains. The pentafluorocyclohexane rings are again offset, one above another, and the rings associate edge to edge at the molecular interface with the rings of another domain of extended molecules forming an infinite bilayer array. Again, the extended aliphatic chains run parallel and are set at an angle of 120° to the plane of the cyclohexane ring in a similar arrangement to that found in **9**. This angle allows the aliphatic chains to approach closely (4.0–4.1 Å), because the equivalent atoms of the cyclohexane rings are spaced more widely (5.57 Å apart), an arrangement that accommodates close van der Waals contacts between the long chains.

## Langmuir isotherm analysis

**Fig. 9:**
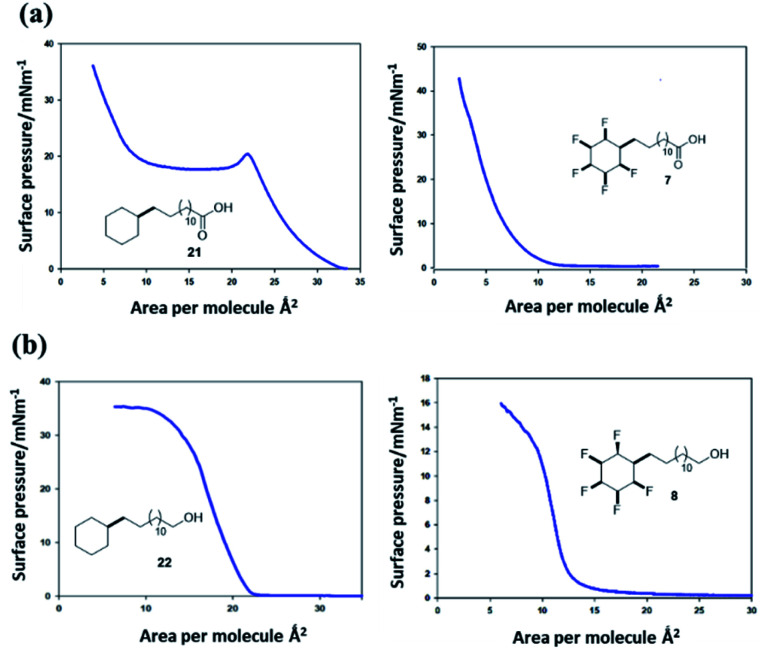
(a) Pressure–area isotherms^26^ on a water subphase of long chain cyclohexyl fatty acids **21** (left) and **7** (right). Fatty acid **21** presents a classical isotherm indicating an initial monolayer collapsing to a bilayer on compression. Fatty acid **7** only displays a low surface area isotherm indicative of a bi- or multi-layer formation. (b) Pressure–area isotherms of long chain cyclohexyl alcohols **22** and **8**. The aliphatic alcohol **22** displays classical monolayer behaviour whereas the fluorinated alcohol **8** displays bilayer behaviour.

Surface pressure–area isotherms^26^ were recorded after deposition of fatty acids **21** and **7** and long chain alcohols **22** and **8** on a water subphase in a Langmuir trough, in order to compare the influence of cyclohexyl fluorination on the surface behaviour (see ESI†). In the first instance the non-fluorinated fatty acid **21** was deposited onto the water subphase from a solution in chloroform, as a reference. The resultant isotherm for **21** is illustrated in Fig. 9a. There is a very clear monolayer transition at about 28–31 Å^2^ per molecule, a little expanded relative to a fatty acid such as stearic acid. With additional compression this monolayer collapses until a second transition is apparent at ∼12 Å^2^ per molecule, suggesting a thermodynamically stable bi- or multi-layer, before infinite compression. Deposition of the fluorocyclohexyl fatty acid **7** required preparation of a pre-solution with 50% acetone in chloroform to overcome its relatively poor solubility. The resultant isotherm in Fig. 9a shows a clear single transition at about 7–9 Å^2^ per molecule for **7**, similar in area to the second compression for the reference fatty acid **21**. The outcome is indicative of the immediate formation of at least a bilayer assembly on the surface. There was no evidence at all for an initial monolayer transition for **7**. This is consistent with pre-aggregation of **7** in the 2D-gas phase.

The long chain alcohols **8** and **22** gave more consistent data due to the increased solubility of **8** as illustrated in isotherms in Fig. 9b. The reference isotherm for the nonfluorinated alcohol **22** indicated well behaved monolayer formation on the aqueous subphase with a condensed phase apparent at around 22–23 Å^2^ per molecule.

**Fig. 10:**
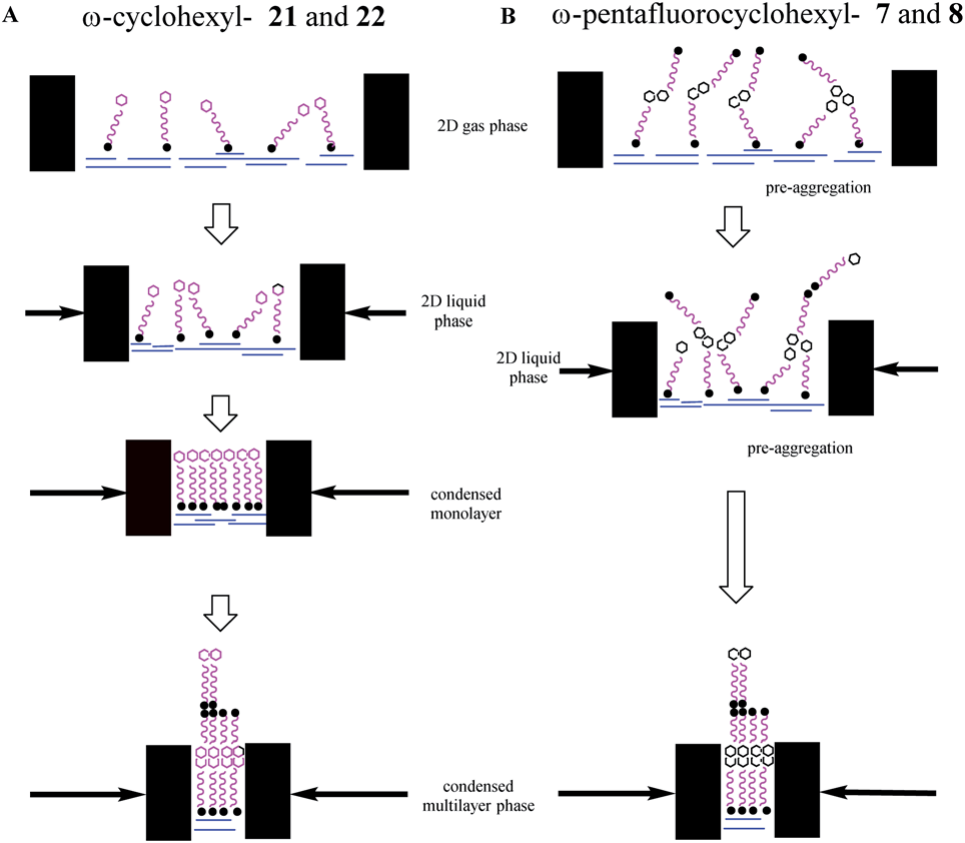
Schematic illustration of the Langmuir isotherms behaviour of the ω-cyclohexyl and ω-pentafluorocyclohexanol fatty acids and alcohols on the water subphase. Arrows indicate the movement of the barriers to reduce surface area. (A) ω-Cyclohexyl fatty acid **21** and alcohol **22** behave classically to generate a coherent condensed monolayer, which further compresses to a multilayer. (B) ω-Pentafluoroyclohexyl fatty acid **7** and alcohol **8** pre-aggregate due to associations between the terminal rings and compression progresses directly to a multilayer.

By comparison, isotherms for the pentafluorinated cyclohexyl alcohol **8** did not show monolayer formation. After deposition from a solution in chloroform, a first phase transition is apparent at an area per molecule of 13–14 Å^2^, below half that for the transition observed for reference alcohol **22**, indicative of a spontaneous bilayer formation. Interestingly, when the long chain alcohol **8** is deposited from a solution which contains acetone (10% by volume in chloroform), then this perturbs the surface isotherm. A first compression is apparent indicating a more classical monolayer, at an area of 24 Å^2^ per molecule. However with further compression a phase transition to the bilayer (at 13 Å^2^) becomes favoured as illustrated in the right hand isotherm in Fig. 9b. It would appear that the more polar solvent (acetone), helps disperse pre-formed aggregations such that a monolayer becomes observable, before reorganisation to a bilayer on compression. Repeated compressions and expansion cycles could not recover any evidence of the monolayer. This is illustrated in Fig. 10.

These experiments suggest that the all-*syn* pentafluorocyclohexyl ring systems **7** and **8** pre-aggregate and generate bilayers directly, whereas the non-fluorinated cyclohexane systems **21** and **22** display classical behaviour. It is clear that the all-*cis* petafluorocyclohexyl ring systems are self-associating. The interaction energy of these rings has been calculated^13^ to be ∼6–8 kcal mol^−1^ and competitive with the strength of hydrogen bonding interactions between the polar head groups and water, and this is consistent with the observed deviation from classical behaviour.

In conclusion, in this study we have prepared the first examples of alkyl substituted 2,3,4,5,6-pentafluorocyclohexanes where all of the substituents are *cis*. The bis ring systems **5** and **6** were prepared and structure **6** with the longer alkyl spaces shows a simpler packing arrangement, in that both rings for each molecule associate with both on adjacent molecules, rather than the more interdigitated arrangement observed in **5**. The very high melting points (>210 °C for **5** and 188 °C for **6**) for these bis-ring systems are striking and is also indicative of the strong intermolecular interactions between these cyclohexane rings. Long chain alkane **9**, alcohol **8** and carboxylic acid **7** were prepared and all had very significantly higher melting points relative to their hydrocarbon analogues. These long chain alkyl derivatives were variously subject to crystal structure and pressure–area isotherm analysis in a Langmuir trough. It is notable that in the X-ray determined structures that were solved for **8** and **9**, and also of the shorter chain alkyls **3** and **4**, that the alkyl substituents always lie equatorial to the all-*cis* pentafluorocyclohexane in a conformation where there are three axial C–F bonds rather than the alternative with two axial C–F bonds and the alkyl group adopting an axial orientation. A theory study indicative of behaviour in the gas phase also supported lower energy equatorial conformers for **3** and **4** despite an increased molecular dipole (polarity) with this arrangement. This augurs well for strong intermolecular associations which are not impeded sterically as there will be stronger electrostatic interactions between the more polar equatorial conformers. This conformation is consistently observed in the solid-state structures presented here. The study illustrates that these functionalised rings constitute a novel motif for the design of supramolecular organic frameworks, a prospect that becomes practical due to improved methods for the synthesis of building blocks by the recent developments in perfluoroaryl hydrogenations.^27^

## Author contributions

**University of St Andrews, UK:** Joshua L. Clark (PhD student) synthesised compounds **4**, **7**, **8** and **9**, **21** and **22**. Cihang Yu (PhD student) made and crystallised compound **3**. Rifahath M. Neyyappadath (post doc) and Ailsa Geddis (Master's student) worked together and made and crystallised compound **5**. David Cordes (SEO) and Alexandra Slawin (Professor) are crystallographers, who between them solved the structures of compounds **3**, **4**, **5**, **6**, **8** & **9**. They also deposited cifs and data at the CCDC. David O'Hagan (Professor) guided the work, arranged the collaborations and wrote the manuscript. **University College London, UK:** Alaric Taylor (post doc) and Stefan Guldin (Professor) are surface scientists at UCL London, where the Langmuir isotherm studies were carried out for compounds **7** and **8** and **21** and **22**. **University of Campinas, Brazil:** Rodrigo Cormanich (Professor) and Bruno Piscelli (PhD student) are theorists based at the University of Campinas in Brazil. They carried out the theory analysis of compounds **3** and **4**.

## Conflicts of interest

There are no conflicts to declare.

## Supplementary Material

SC-012-D1SC02130C-s001

SC-012-D1SC02130C-s002

SC-012-D1SC02130C-s003
