# Supplementary material for: Supramolecular packing of alkyl substituted Janus face all-cis 2,3,4,5,6-pentafluorocyclohexyl motifs
Source: Chem Sci. 2021 Jun 4;12(28):9712–9. doi: 10.1039/d1sc02130c (PMC8293821; doi:10.1039/d1sc02130c)

Electronic Supplementary Material (ESI) for Chemical Science.
This journal is © The Royal Society of Chemistry 2021

NMR Spectra

(1R,2R,3s,4S,5S,6r)-1,2,3,4,5-pentafluoro-6-methylcyclohexane (**3**)

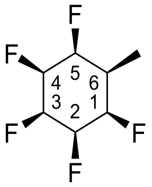

^{1}H NMR (400 MHz, Acetone-d_6)

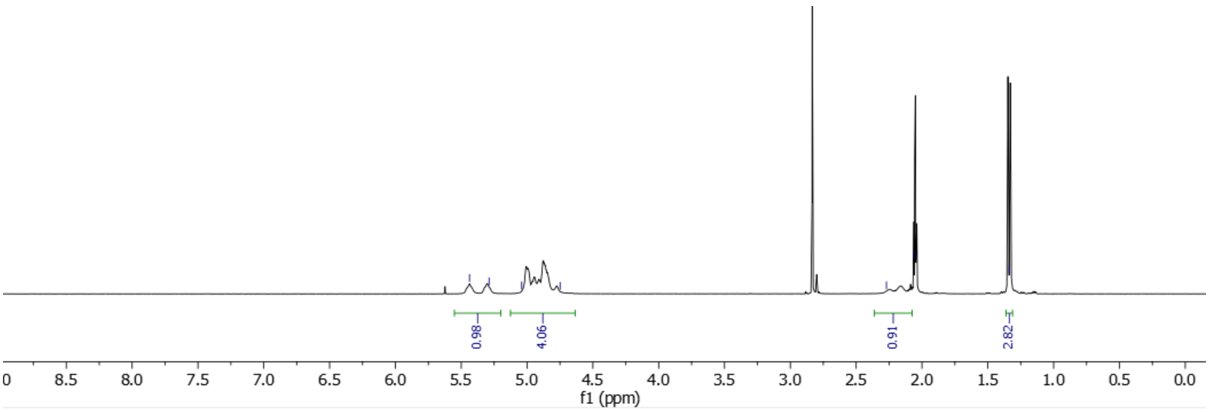

^{19}F NMR (377 MHz, Acetone-d_6)

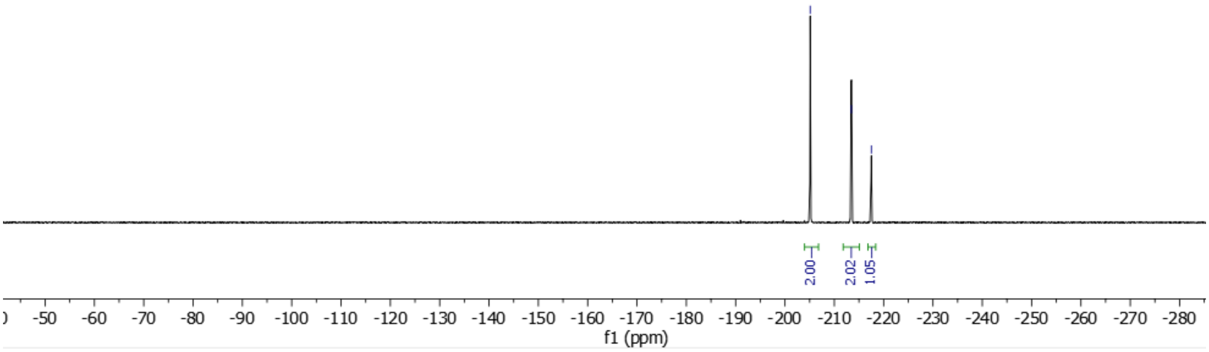

^{13}C NMR (101 MHz, Acetone)

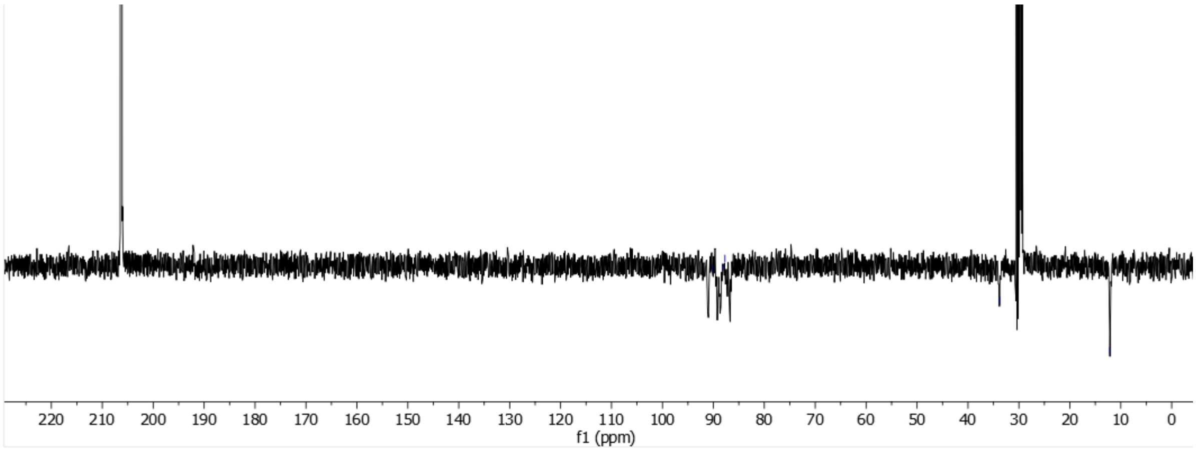

220 210 200 190 180 170 160 150 140 130 120 110 100 90 80 70 60 50 40 30 20 10 0
f1 (ppm)

(1r,2R,3R,4s,5S,6S)-1-ethyl-2,3,4,5,6-pentafluorocyclohexane (**4**)

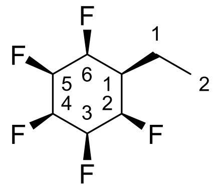

^{1}H NMR (400 MHz, Acetone)

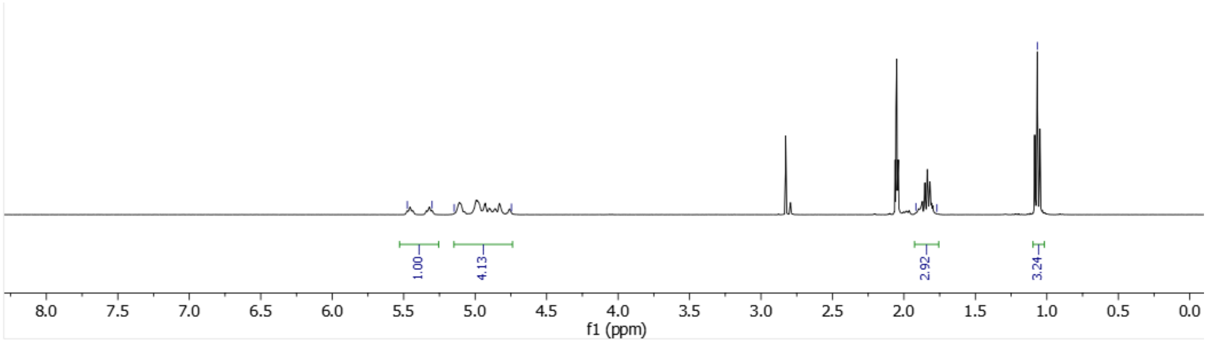

^{13}C NMR (176 MHz, Acetone)

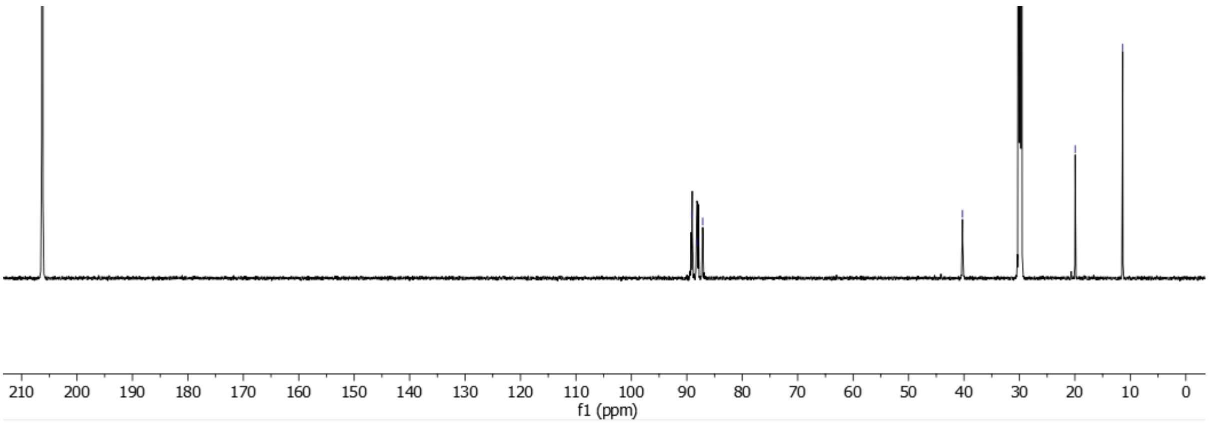

^{19}F NMR (377 MHz, Acetone)

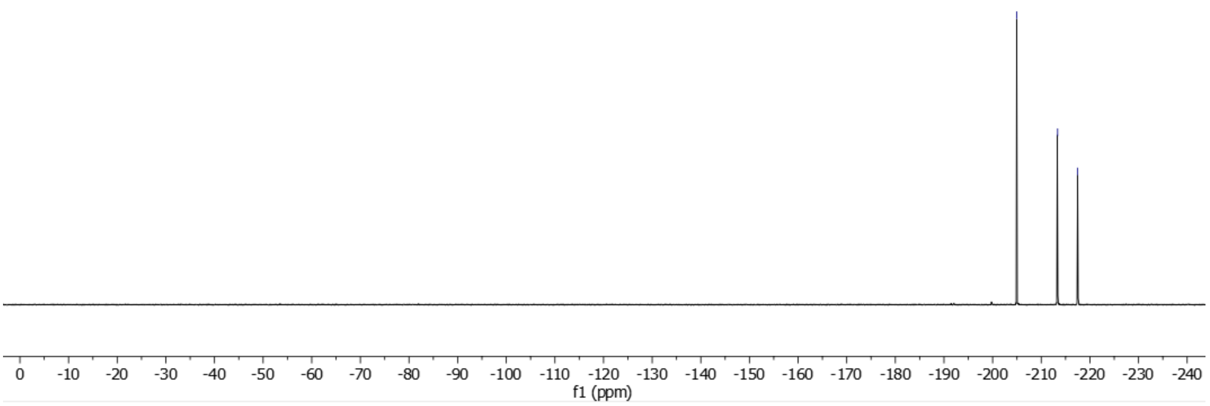

(E)-1,2-bis(perfluorophenyl)ethene (**11**)

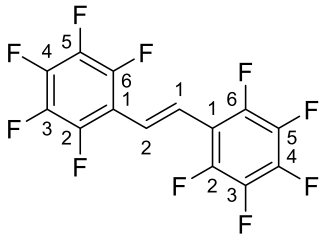

^{1}H NMR (400 MHz, Acetone)

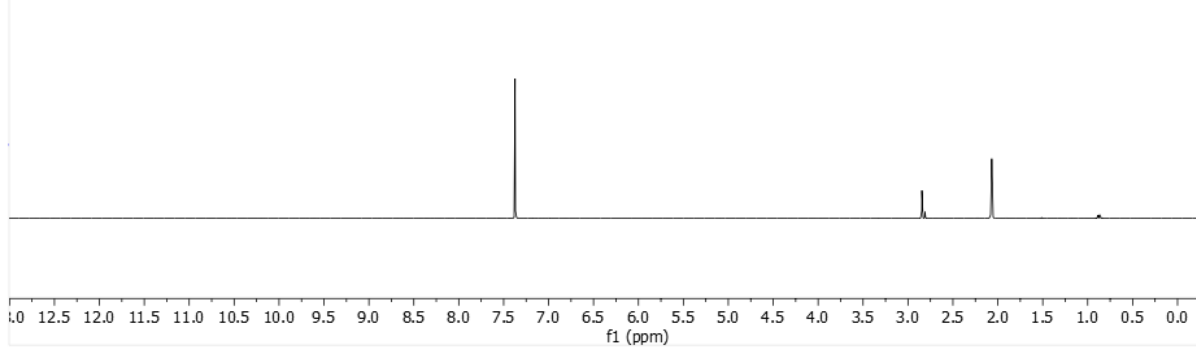

^{19}F NMR (376 MHz, Acetone)

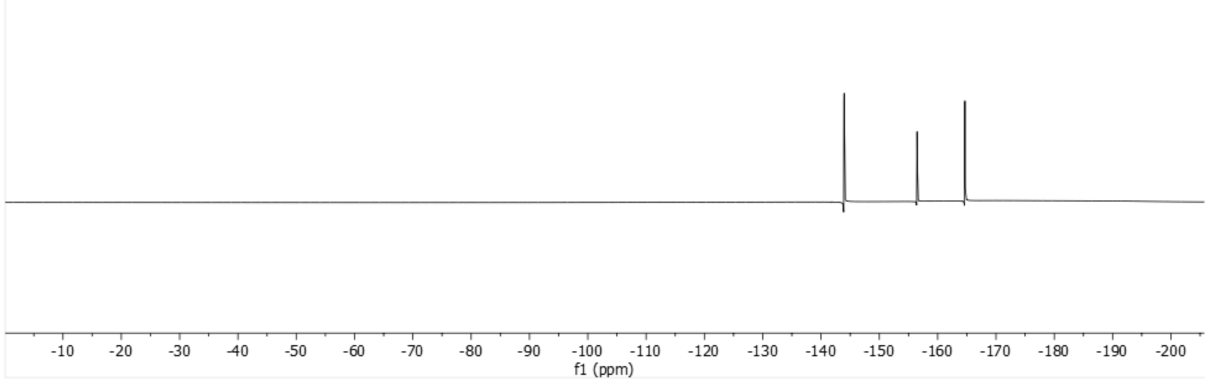

1,2-bis((1r,2R,3R,4S,5S,6S)-2,3,4,5,6-pentafluorocyclohexyl)ethane (**5**)

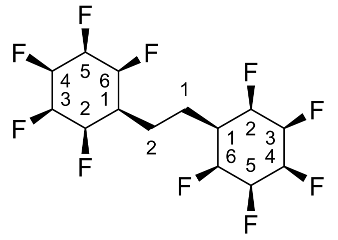

^{1}H NMR (400 MHz, DMSO)

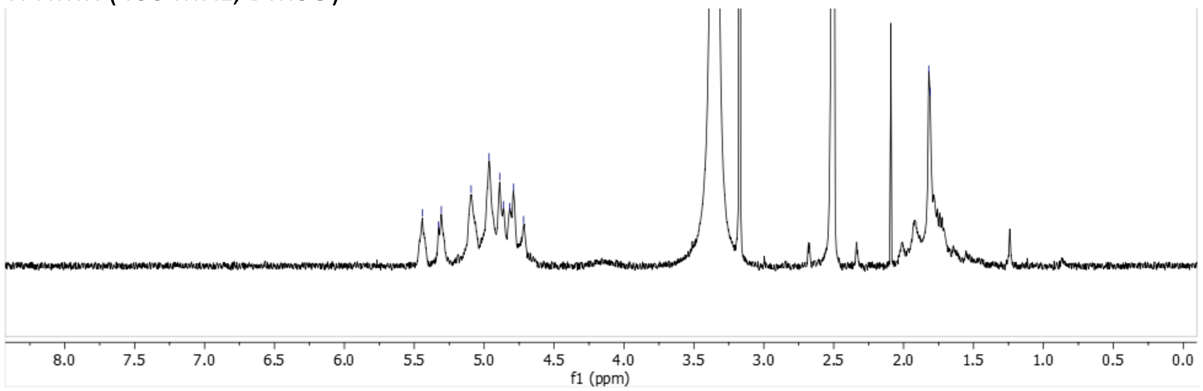

^{19}F NMR (376 MHz, DMSO)

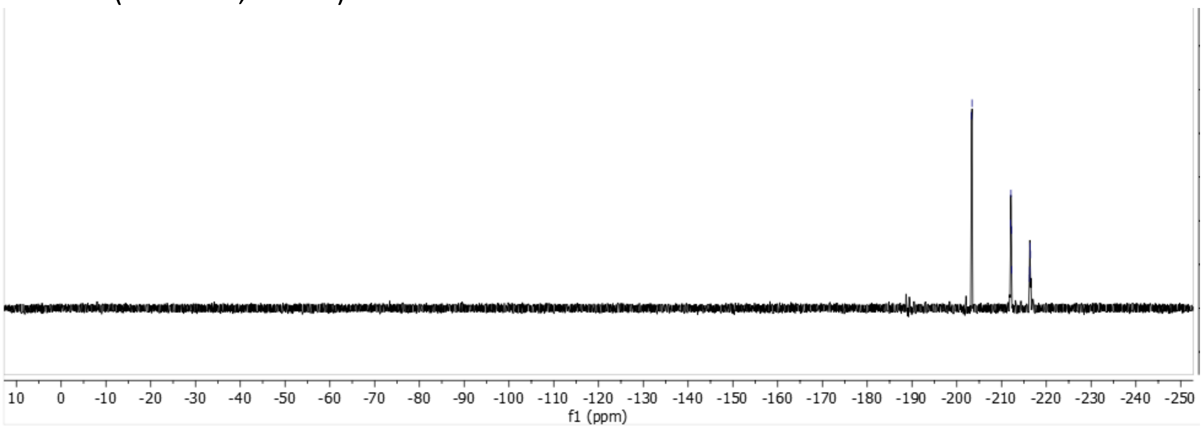

methyl E-11-(perfluorophenyl)undec-10-enoate (**13**)

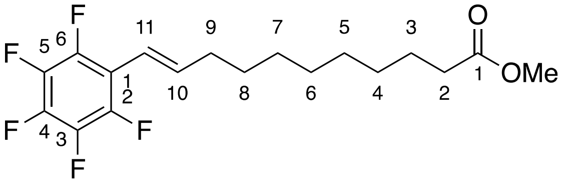

^{1}H {^{19}F} NMR (500 MHz, $CDCl_3$)

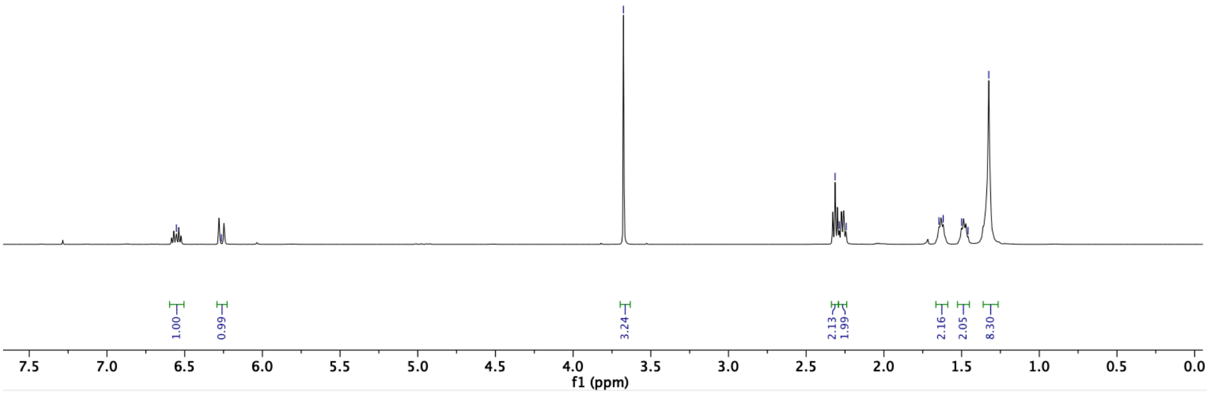

^{13}C NMR (126 MHz, $CDCl_3$)

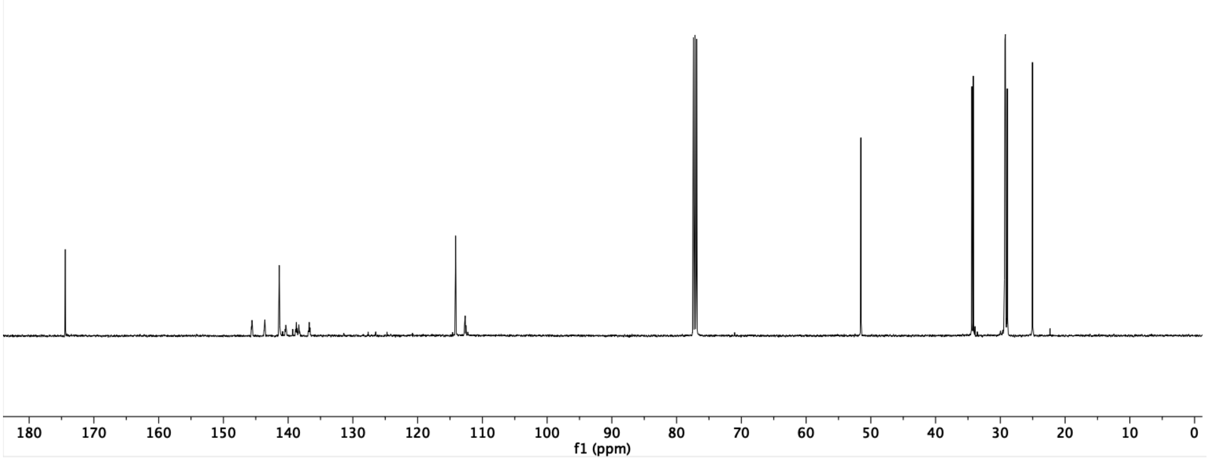

^{19}F {^{1}H} NMR (470 MHz, $CDCl_3$)

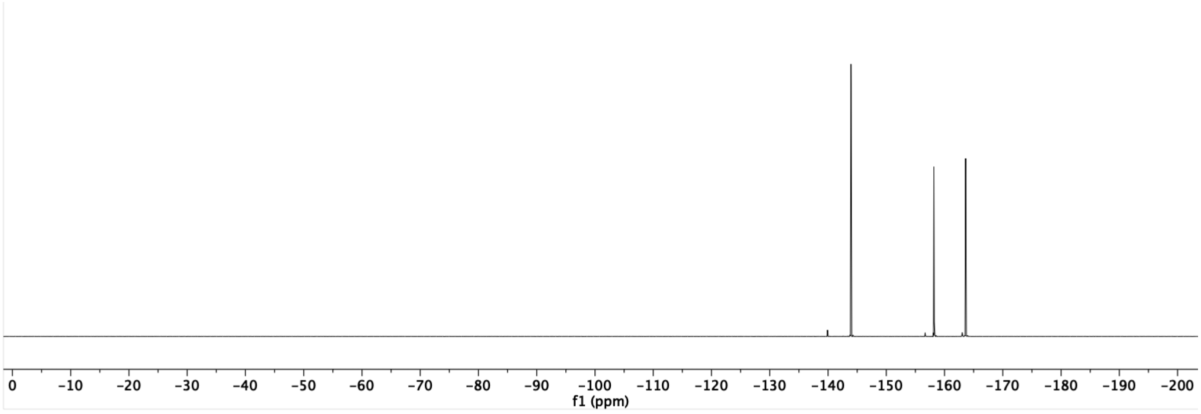

E-11-(perfluorophenyl)undec-10-enal (**14**)

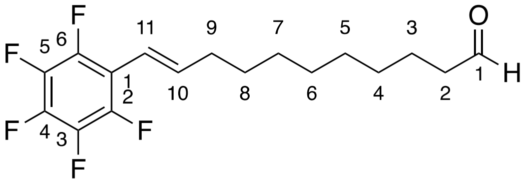

^{1}H NMR (700 MHz, $CDCl_3$)

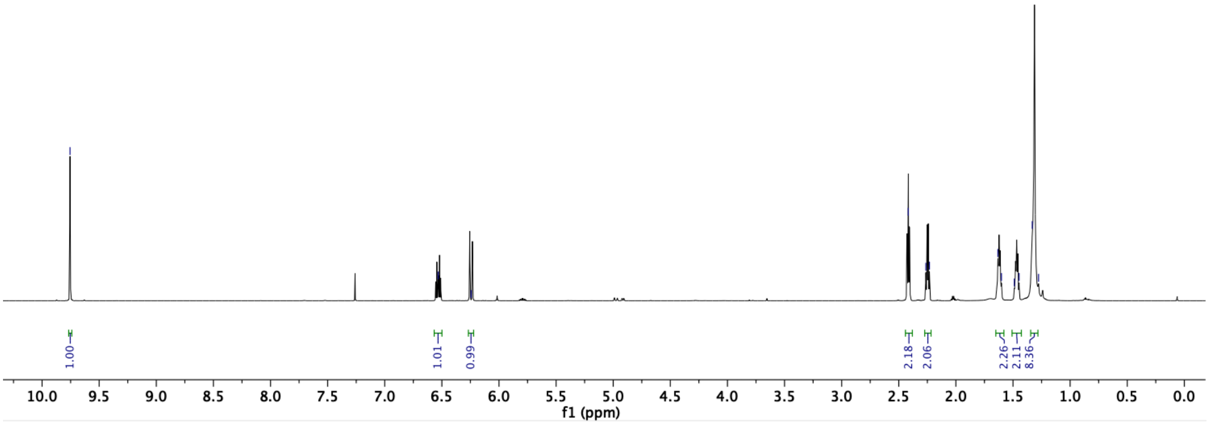

^{19}F NMR (377 MHz, $CDCl_3$)

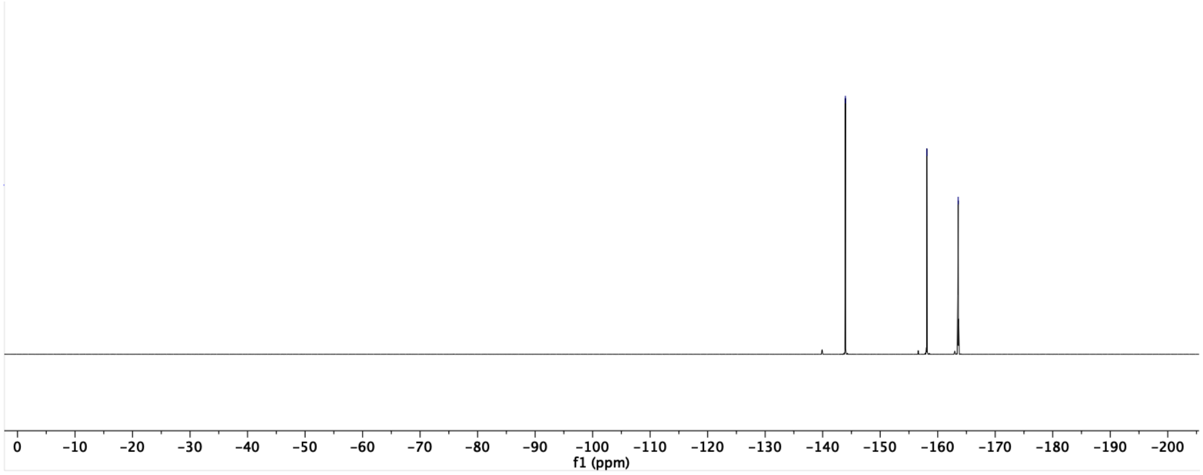

^{13}C NMR (101 MHz, $CDCl_3$)

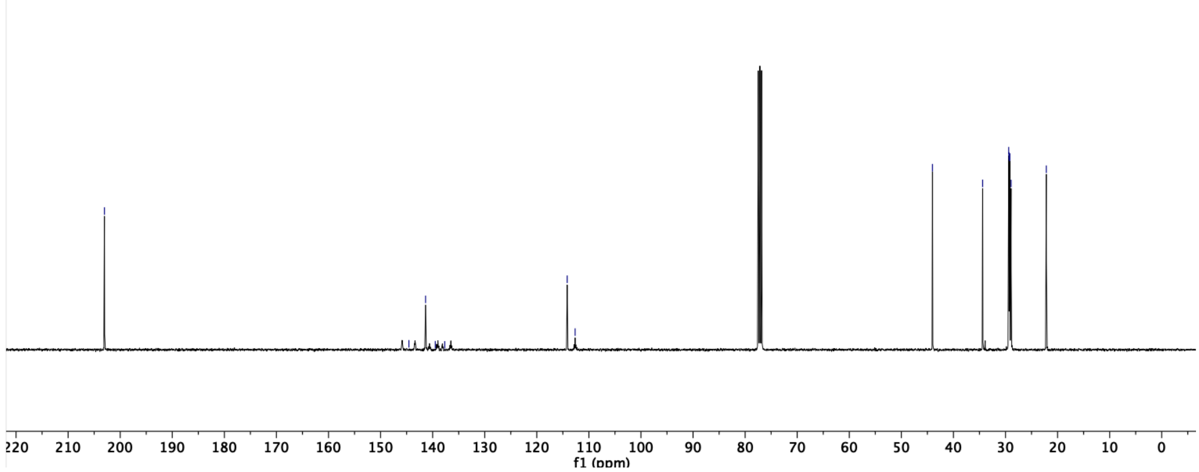

diethyl ((perfluorophenyl)methyl)phosphonate (**15**)

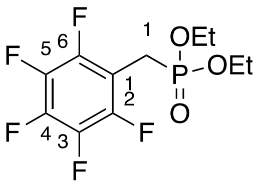

^{1}H NMR (300 MHz, $CDCl_3$)

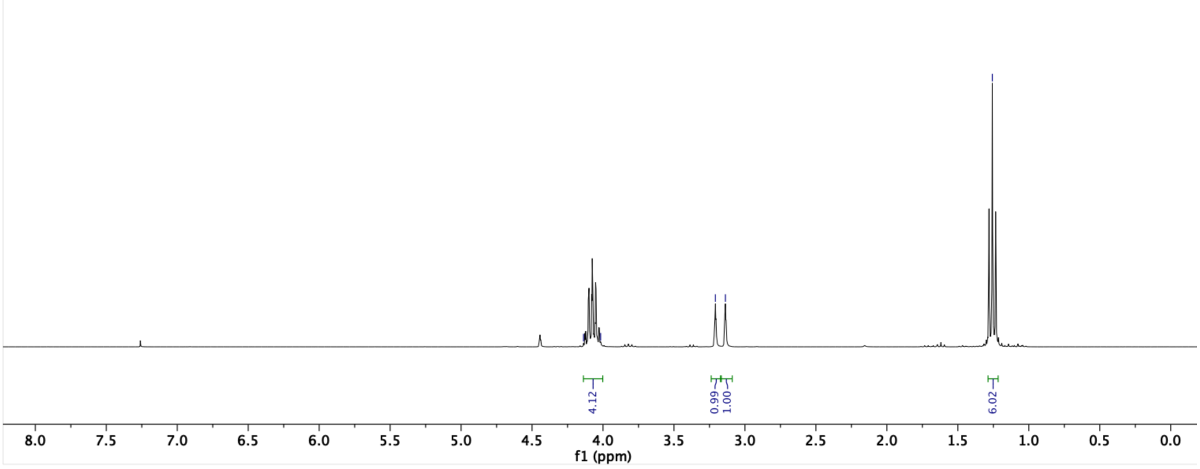

^{13}C NMR (126 MHz, $CDCl_3$)

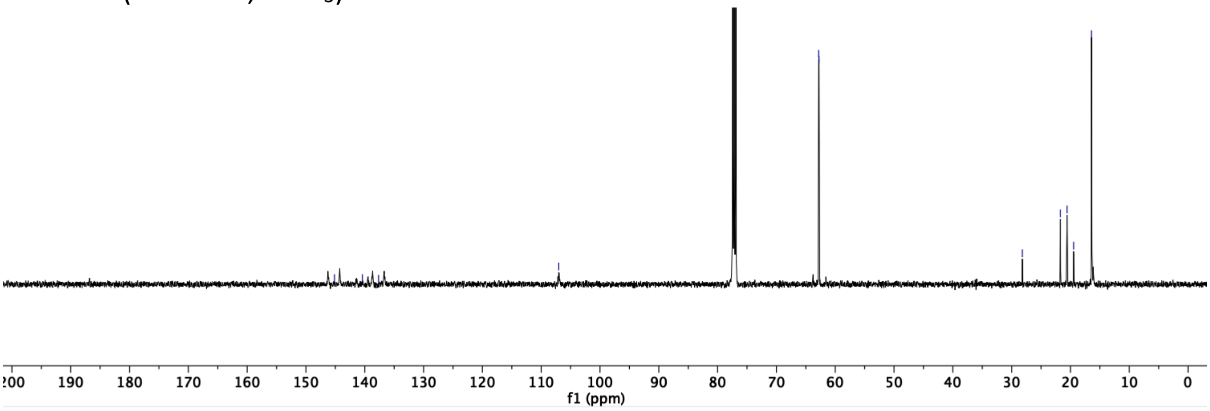

^{31}P NMR (162 MHz, $CDCl_3$)

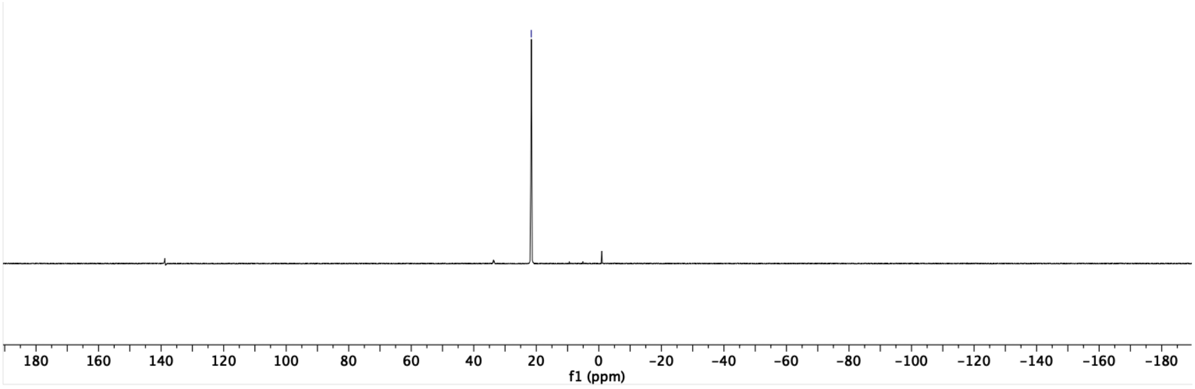

^{19}F NMR (376 MHz, $CDCl_3$)

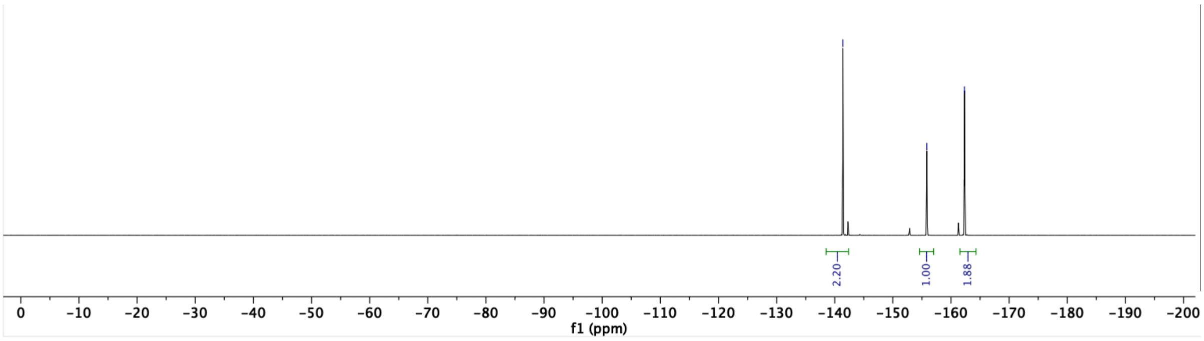

2.20
1.00
1.88
0
−10
−20
−30
−40
−50
−60
−70
−80
−90
−100
−110
−120
−130
−140
−150
−160
−170
−180
−190
−200
f1 (ppm)

(1E,11E)-1,12-bis(perfluorophenyl)dodeca-1,11-diene (**16**)

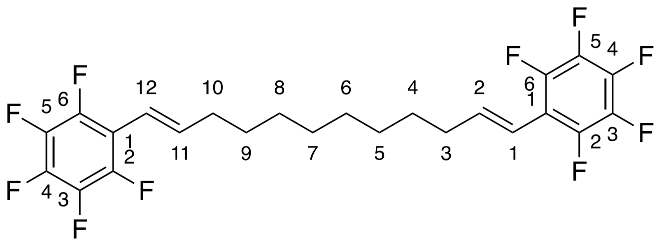

^{1}H NMR (700 MHz, $CDCl_3$)

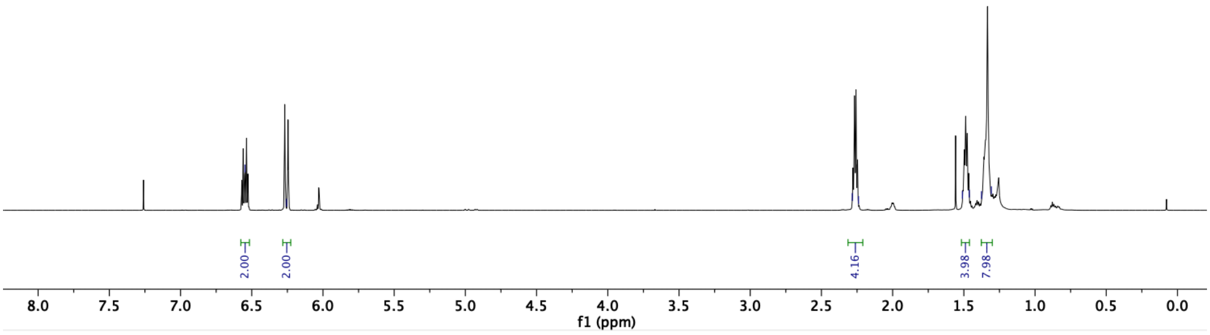

^{19}F NMR (471 MHz, $CDCl_3$)

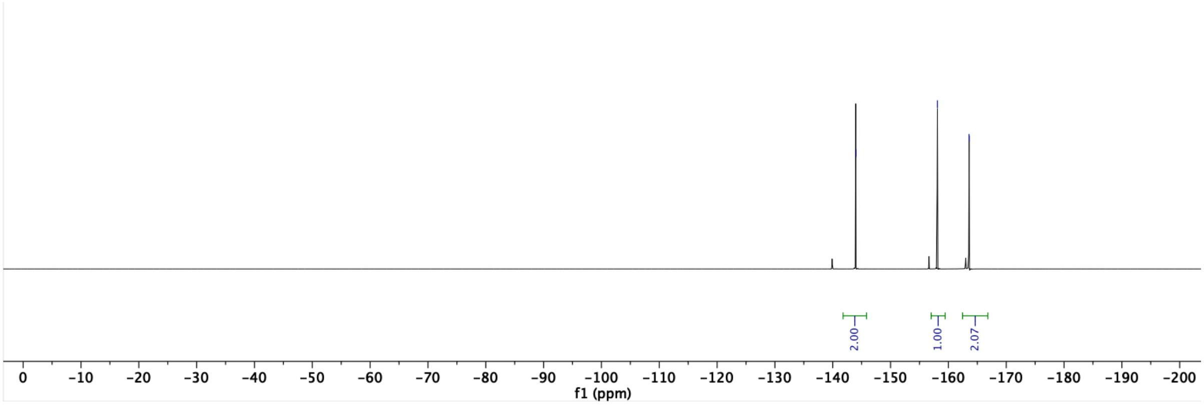

^{13}C NMR (176 MHz, $CDCl_3$)

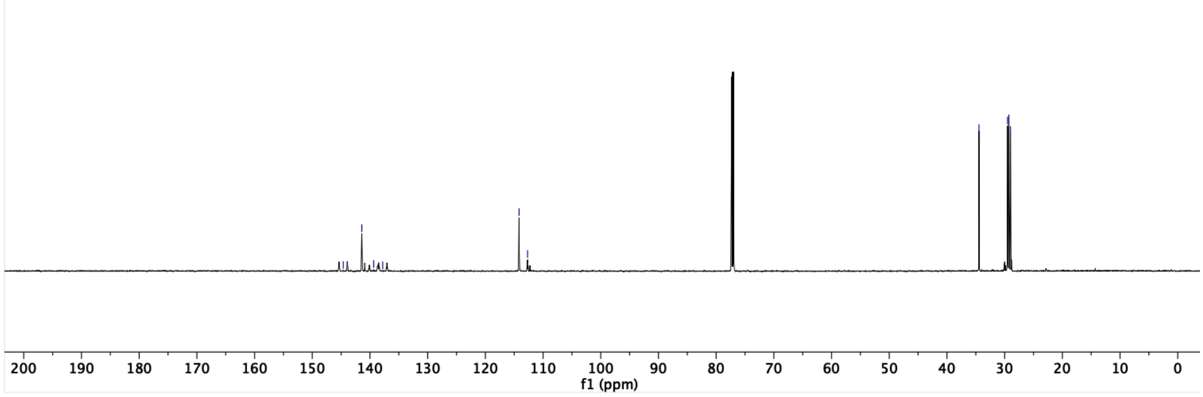

Synthesis of 1,12-bis((1r,2R,3R,4s,5S,6S)-2,3,4,5,6-pentafluorocyclohexyl)dodecane (**6**)

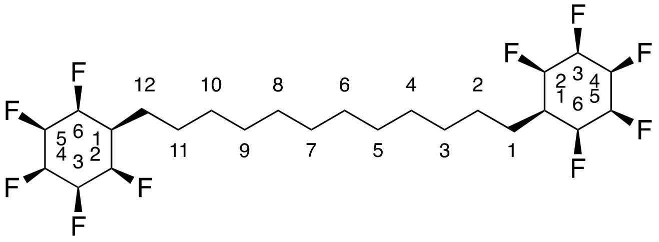

^{1}H NMR (500 MHz, $CDCl_3$)

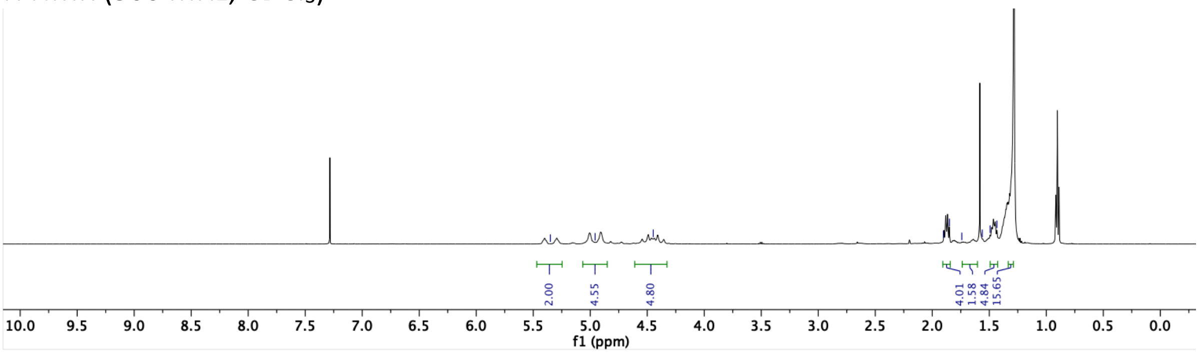

^{19}F NMR (471 MHz, Acetone)

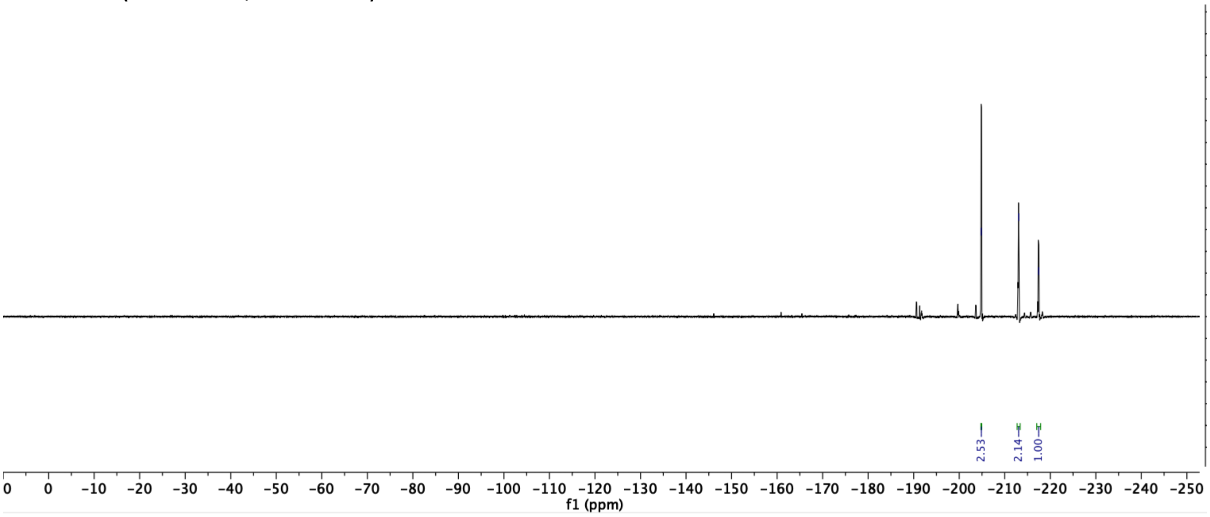

^{13}C NMR (126 MHz, $CDCl_3$)

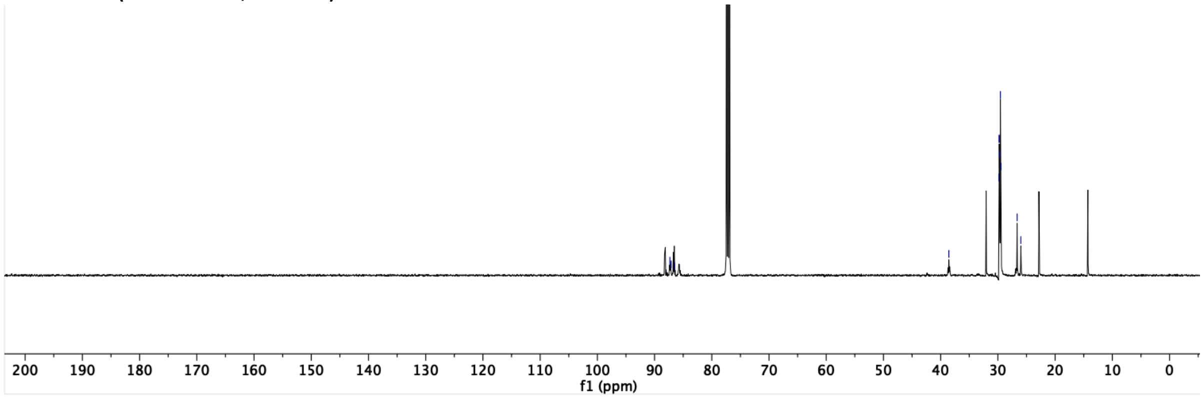

methyl 13-phenyltridec-10-enoate (**19**)

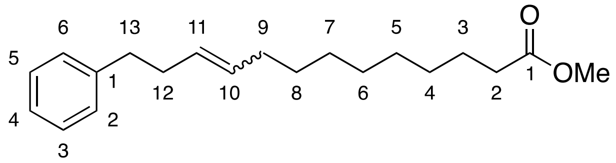

^{1}H NMR (500 MHz, $CDCl_3$)

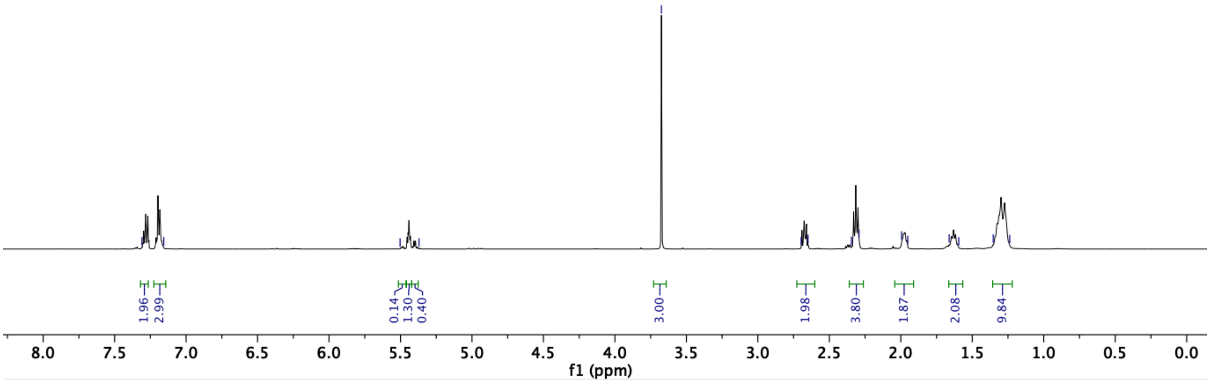

^{13}C NMR (126 MHz, $CDCl_3$)

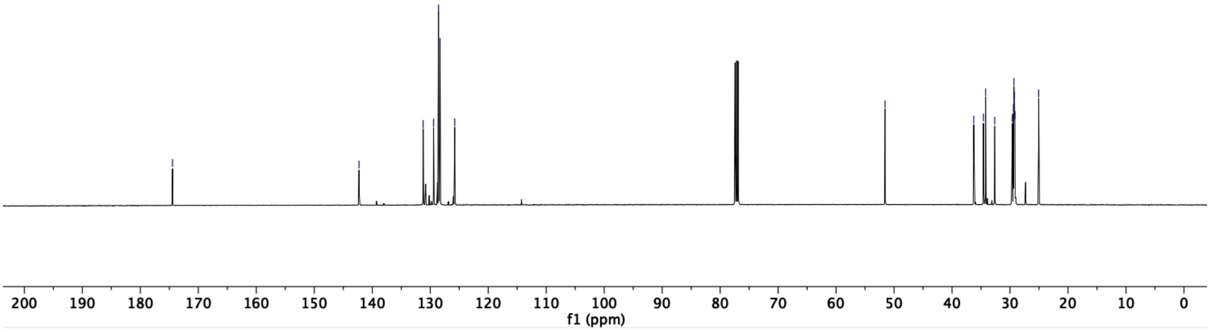

methyl 13-cyclohexyltridecanoate (**20**)

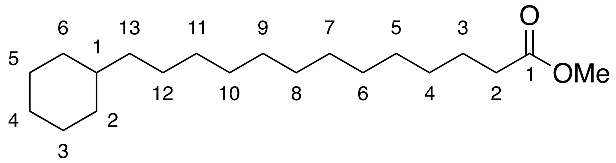

^{1}H NMR (500 MHz, $CDCl_3$)

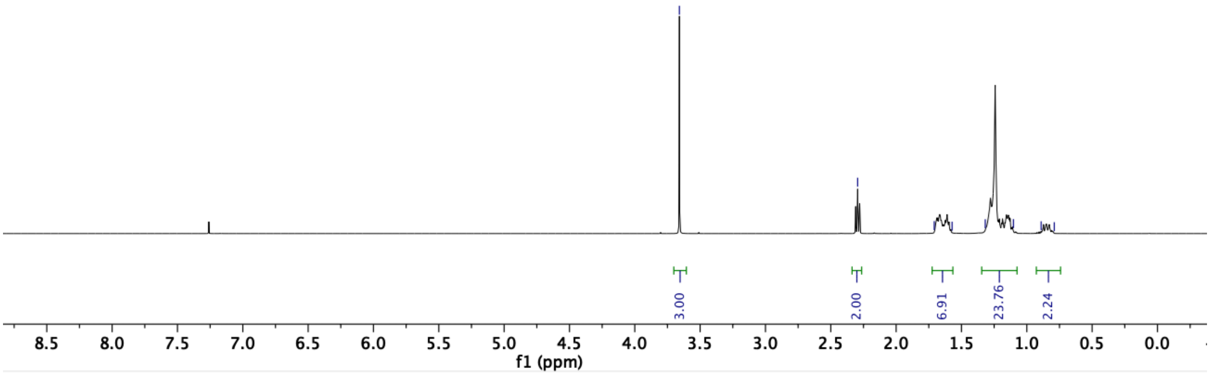

^{13}C NMR (126 MHz, $CDCl_3$)

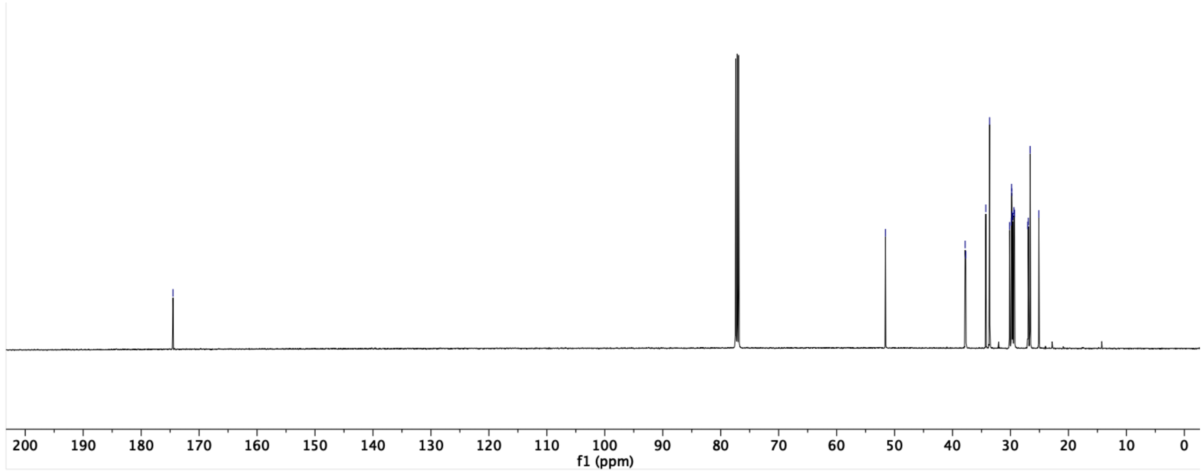

Synthesis of 13-cyclohexyltridecanoic acid (**21**)

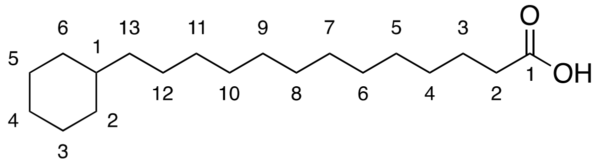

^{1}H NMR (500 MHz, $CDCl_3$)

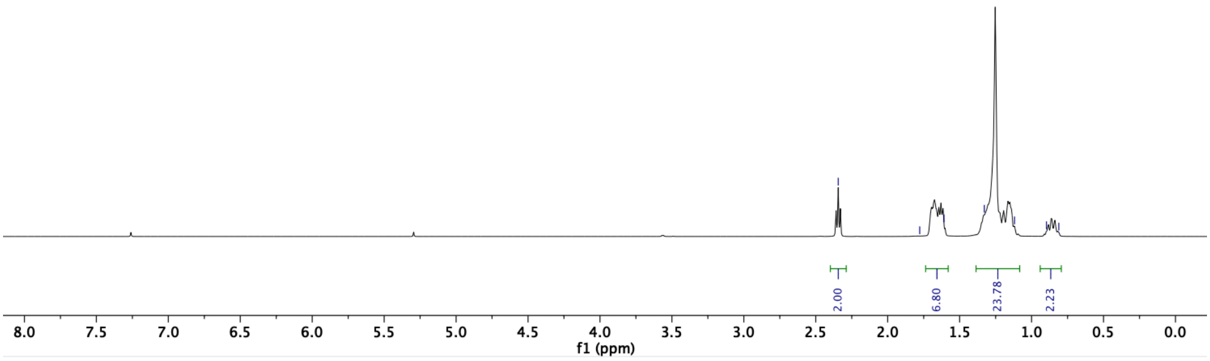

^{13}C NMR (126 MHz, $CDCl_3$)

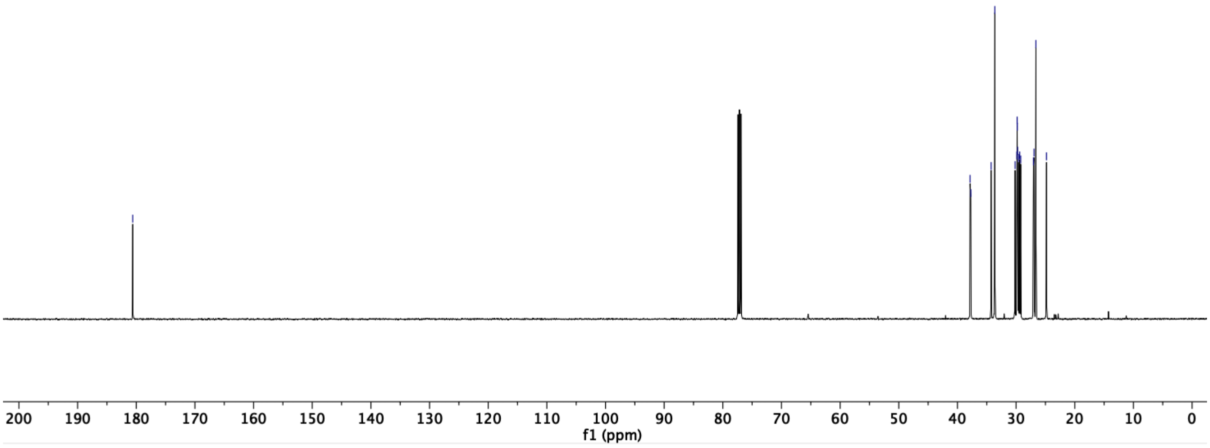

Synthesis of 13-cyclohexyltridecan-1-ol (**22**)

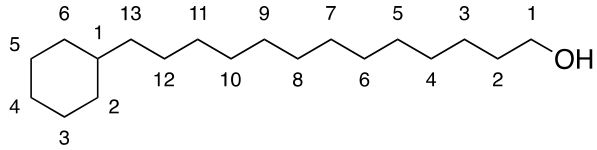

^{1}H NMR (500 MHz, $CDCl_3$)

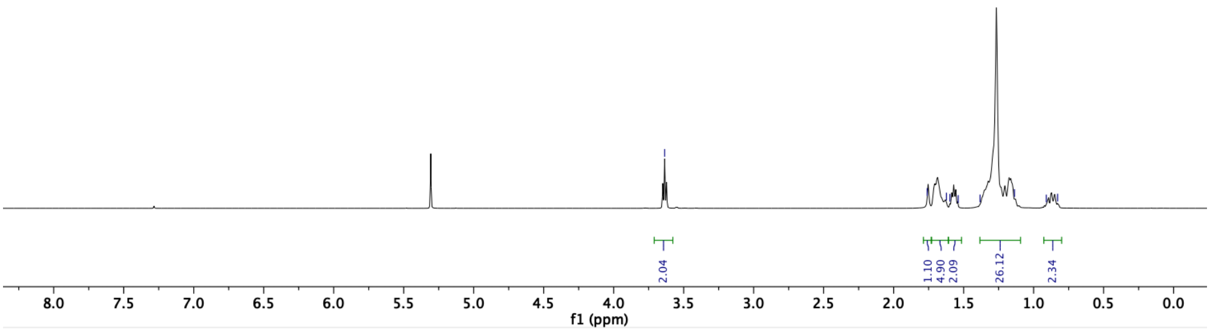

^{13}C NMR (126 MHz, $CDCl_3$)

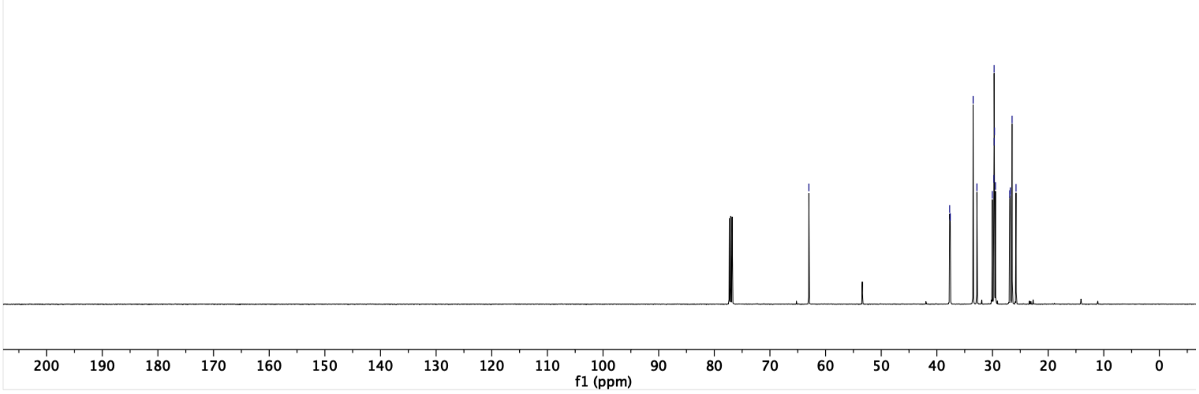

Synthesis of methyl 12-hydroxydodecanoate (**23**)

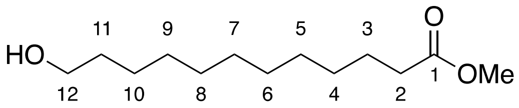

^{1}H NMR (500 MHz, $CDCl_3$)

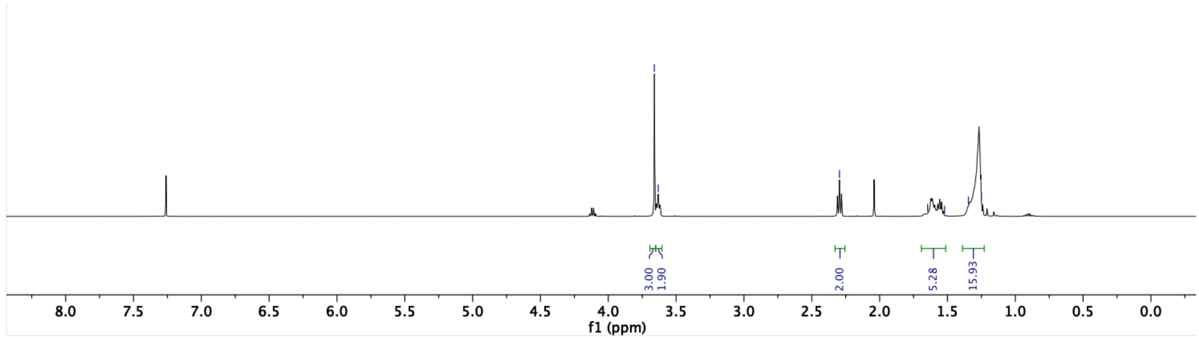

^{13}C NMR (126 MHz, $CDCl_3$)

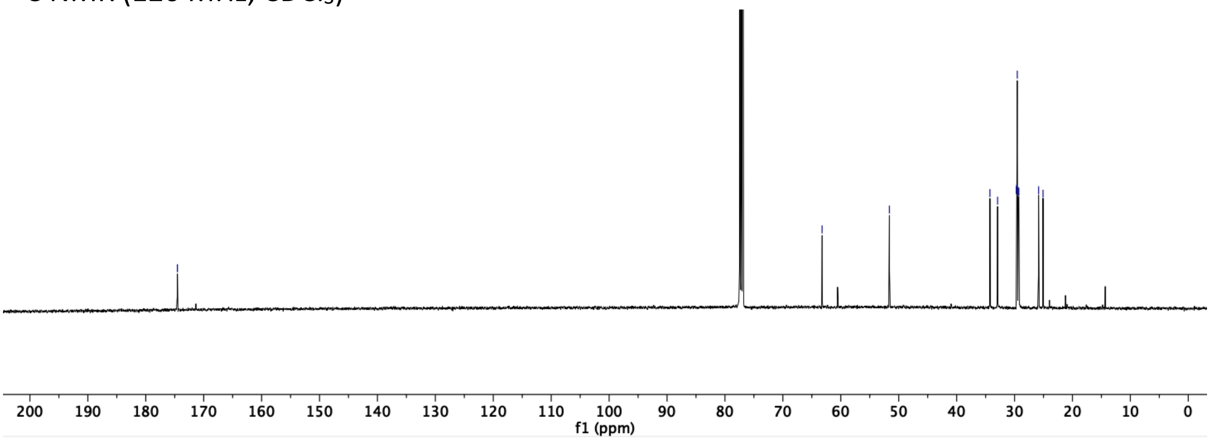

Synthesis of methyl 12-oxododecanoate (**24**)

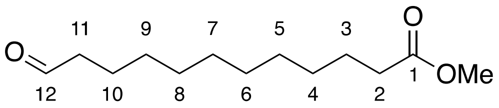

^{1}H NMR (500 MHz, $CDCl_3$)

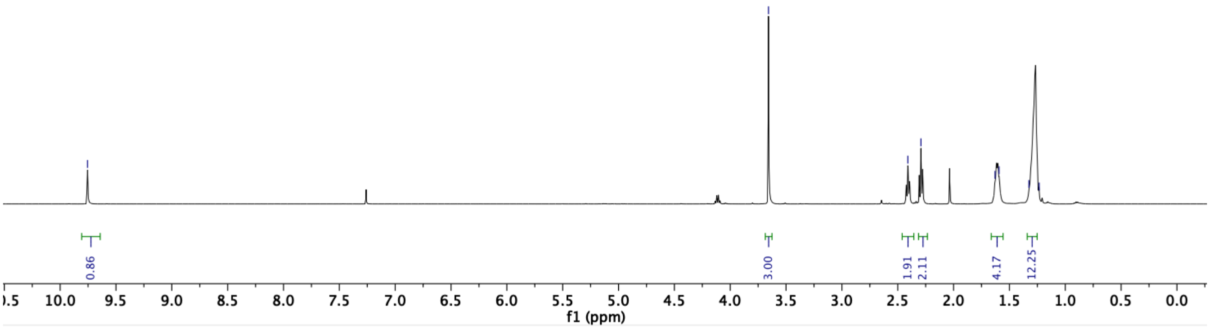

^{13}C NMR (126 MHz, $CDCl_3$)

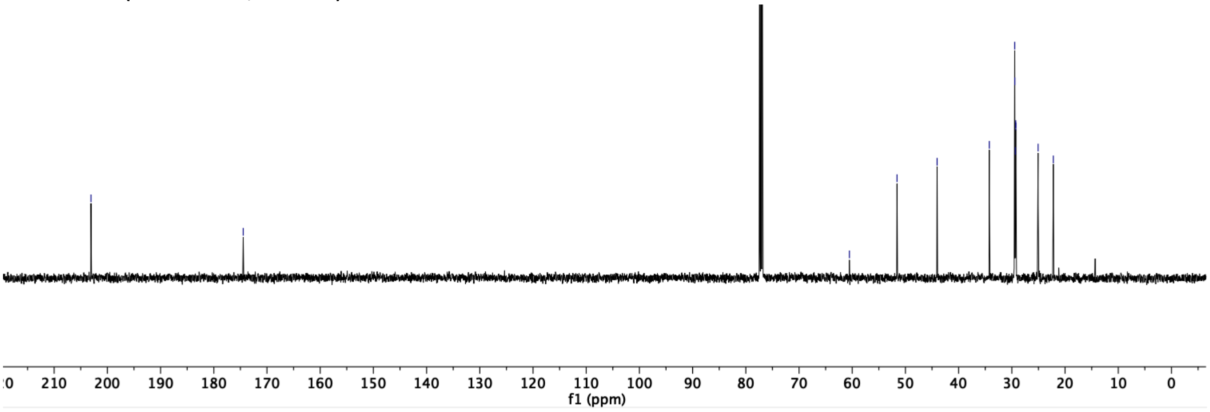

Synthesis of methyl *E*-13-(perfluorophenyl)tridec-12-enoate (**27**)

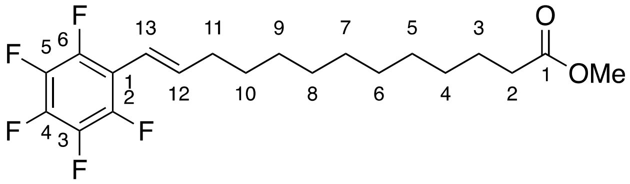

^{1}H NMR (500 MHz, $CDCl_3$)

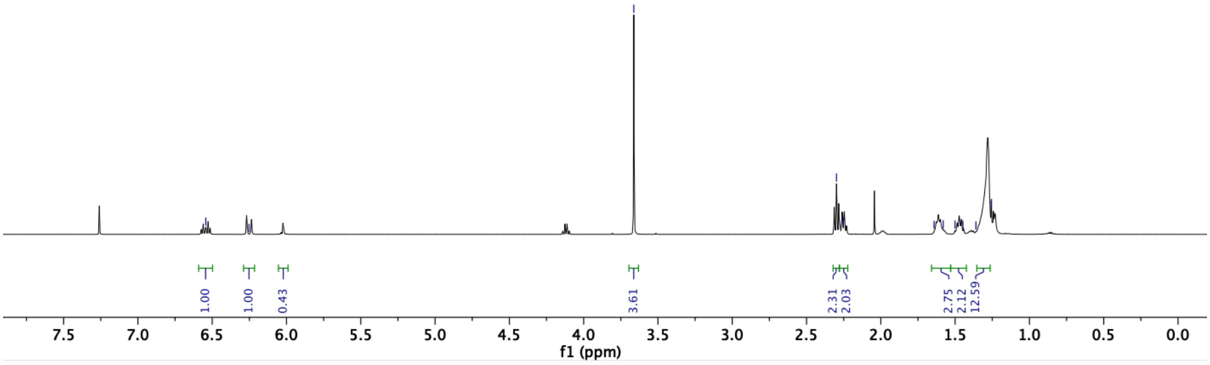

^{13}C NMR (126 MHz, $CDCl_3$)

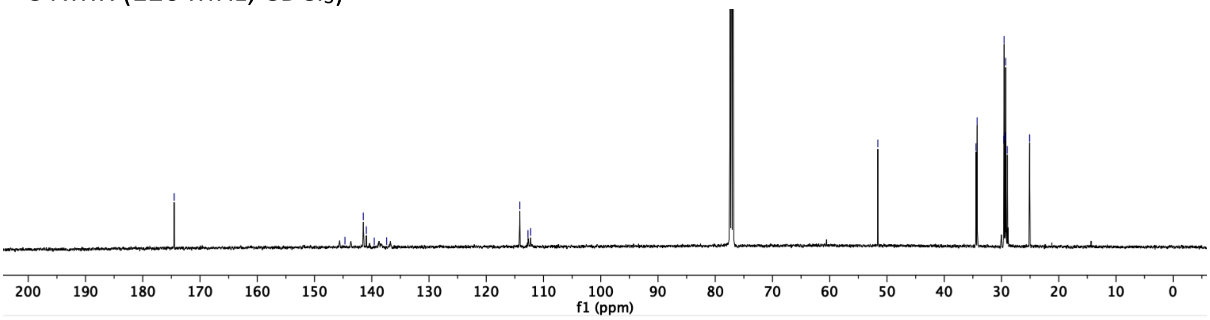

^{19}F NMR (471 MHz, $CDCl_3$)

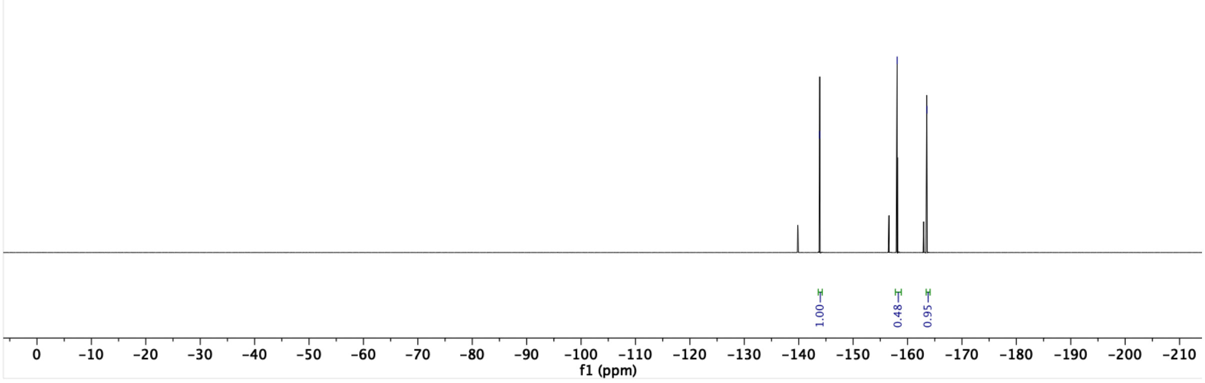

methyl 13-((1r,2R,3R,4s,5S,6S)-2,3,4,5,6-pentafluorocyclohexyl)tridecanoate (**28**)

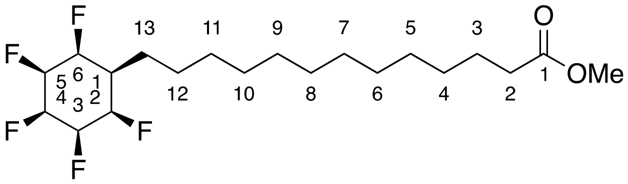

^{1}H NMR (500 MHz, CDCl$_3$)

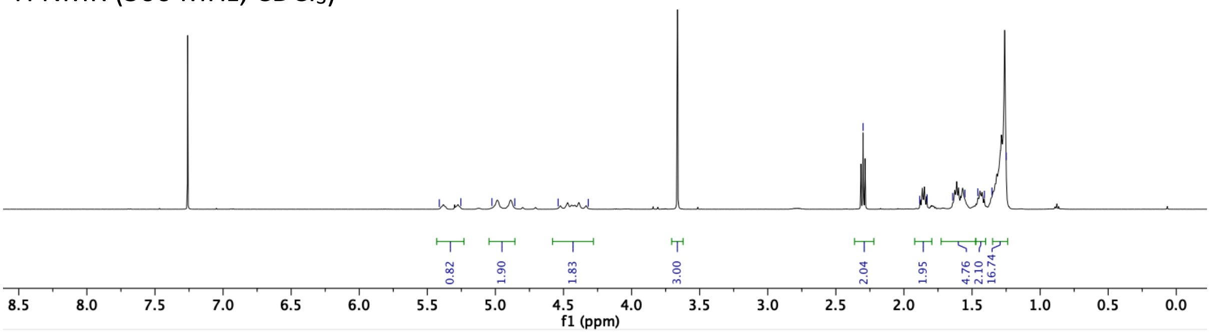

^{19}F NMR (471 MHz, CDCl$_3$)

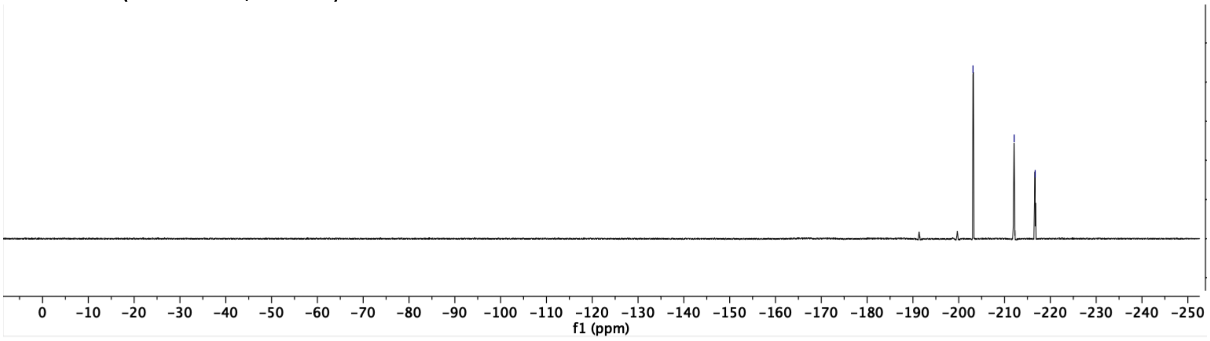

^{13}C NMR (126 MHz, CDCl$_3$)

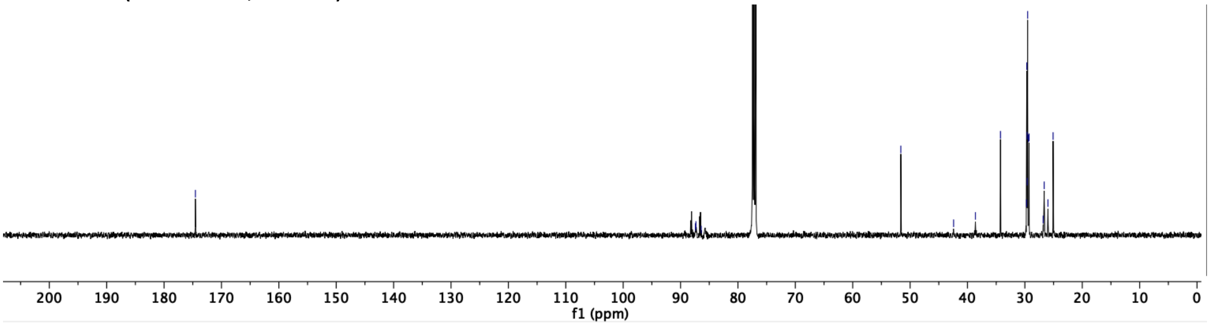

13-((1r,2R,3R,4s,5S,6S)-2,3,4,5,6-pentafluorocyclohexyl)tridecanoic acid (**7**)

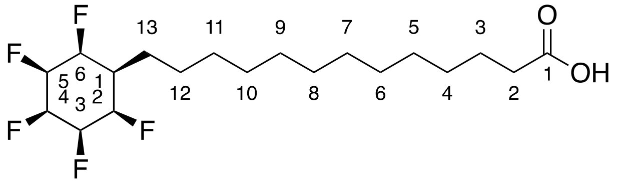

^{1}H NMR (500 MHz, Acetone)

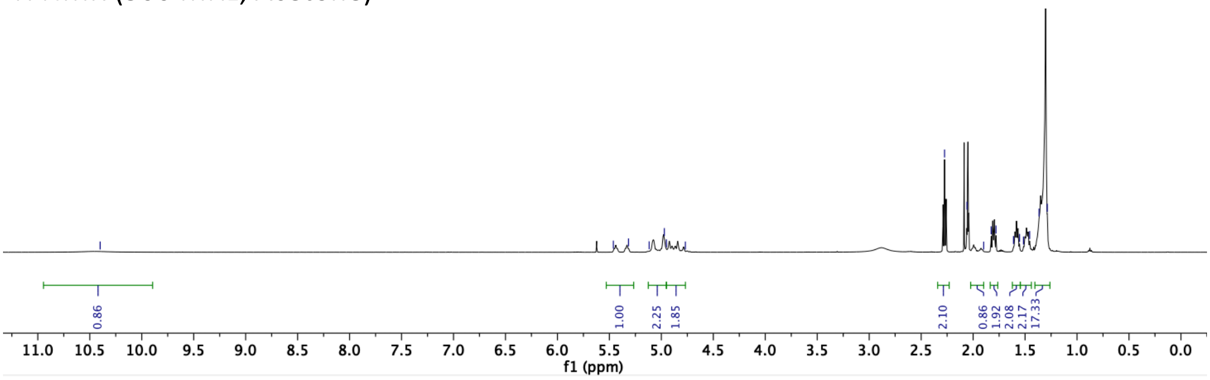

^{19}F NMR (471 MHz, Acetone)

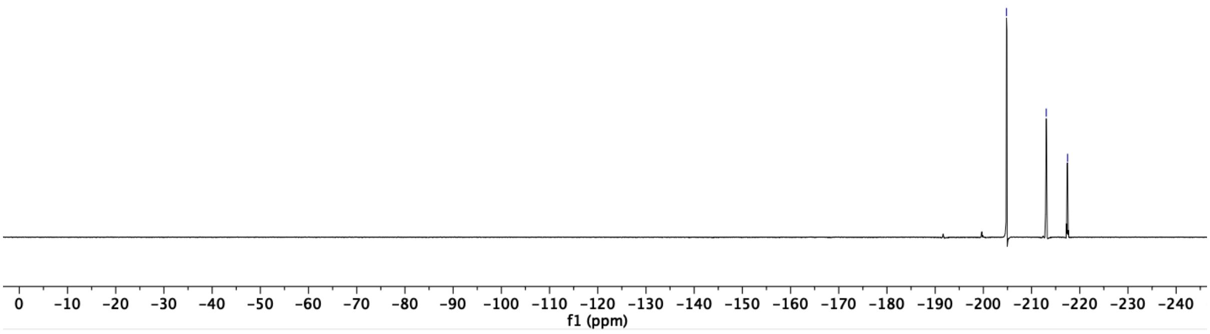

^{13}C NMR (126 MHz, Acetone)

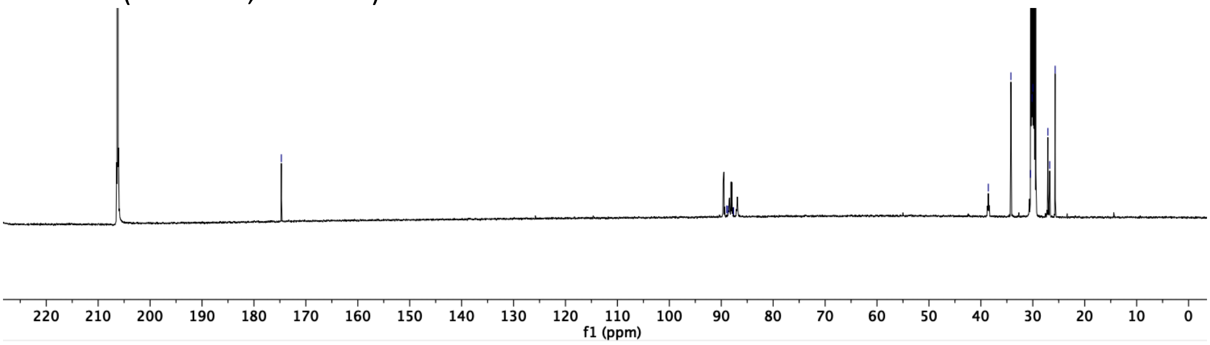

13-((1r,2R,3R,4s,5S,6S)-2,3,4,5,6-pentafluorocyclohexyl)tridecan-1-ol (**8**)

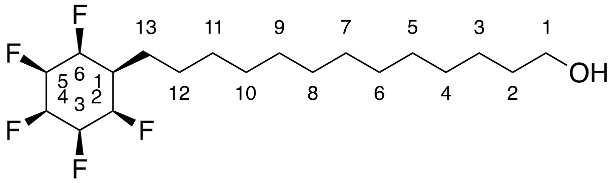

^{1}H NMR (500 MHz, Acetone)

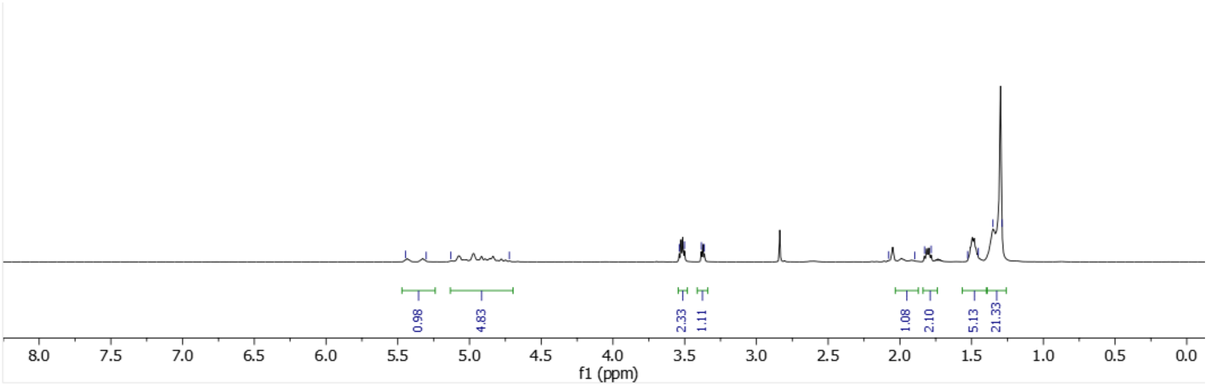

^{19}F NMR (470 MHz, Acetone)

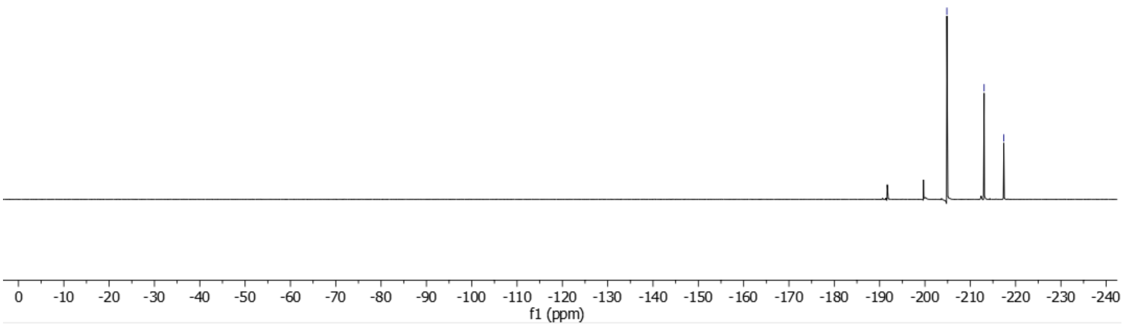

^{13}C NMR (126 MHz, Acetone)

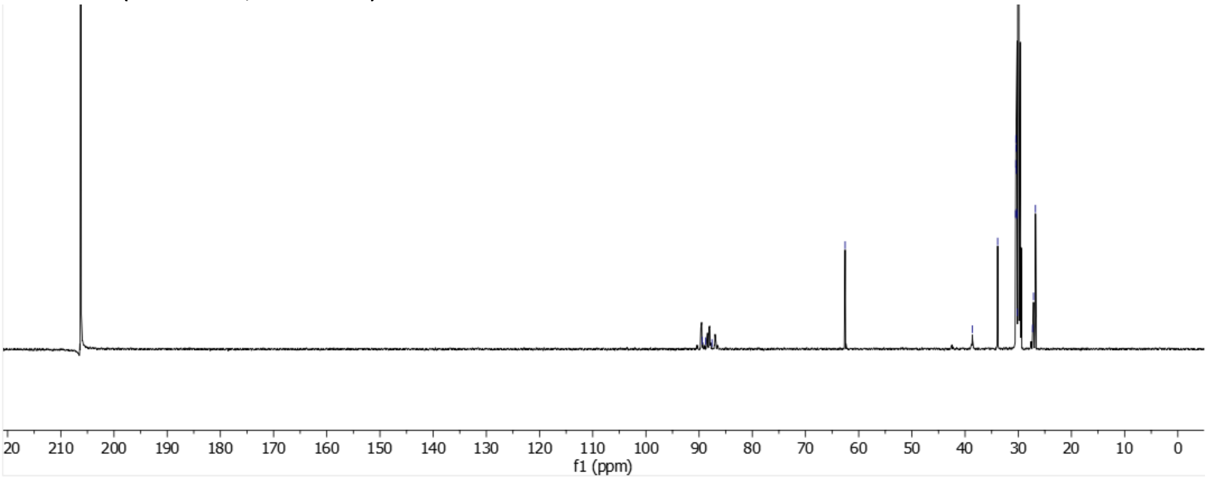

(1R,2R,3s,4S,5S,6r)-1,2,3,4,5-pentafluoro-6-tridecylcyclohexane **9**

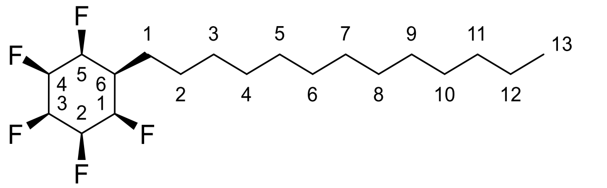

^{1}H NMR (400 MHz, $CDCl_3$)

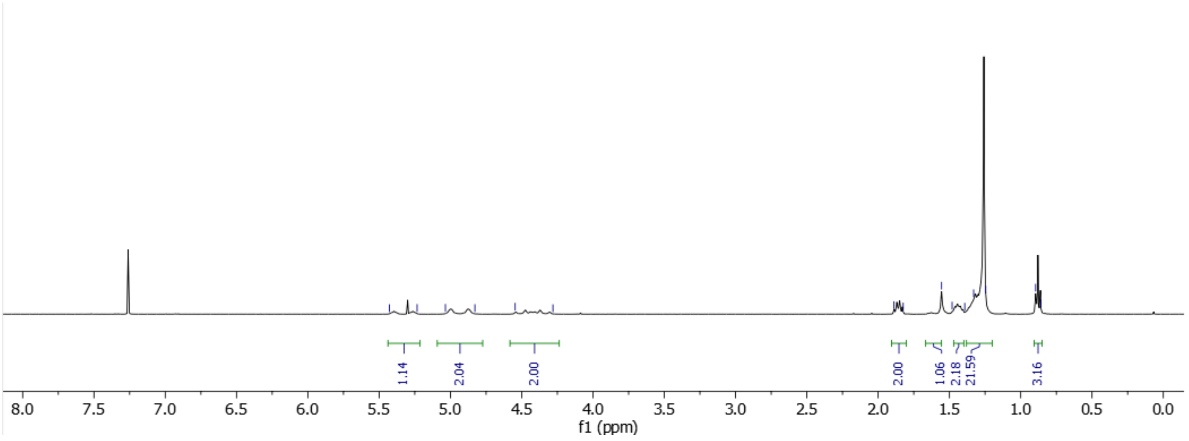

^{13}C NMR (101 MHz, $CDCl_3$)

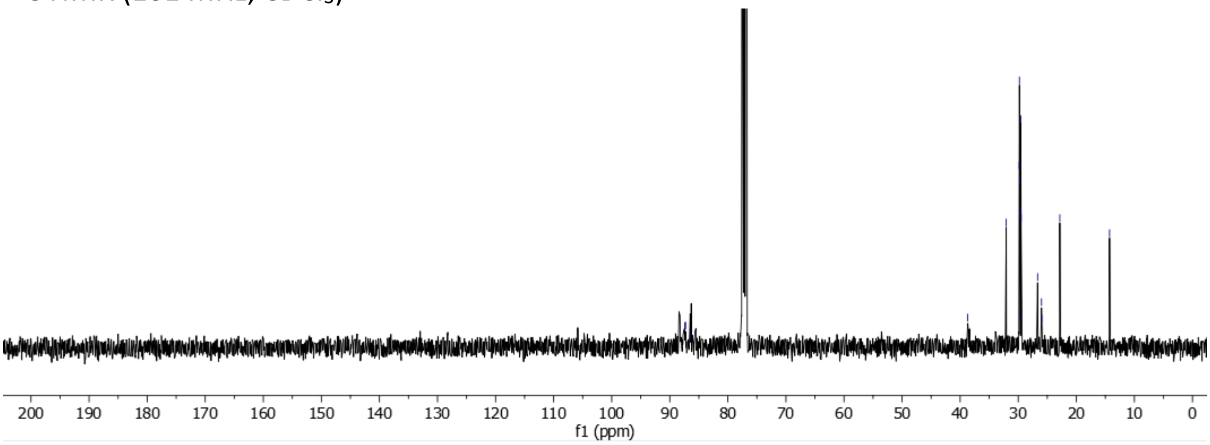

^{19}F NMR (376 MHz, $CDCl_3$)

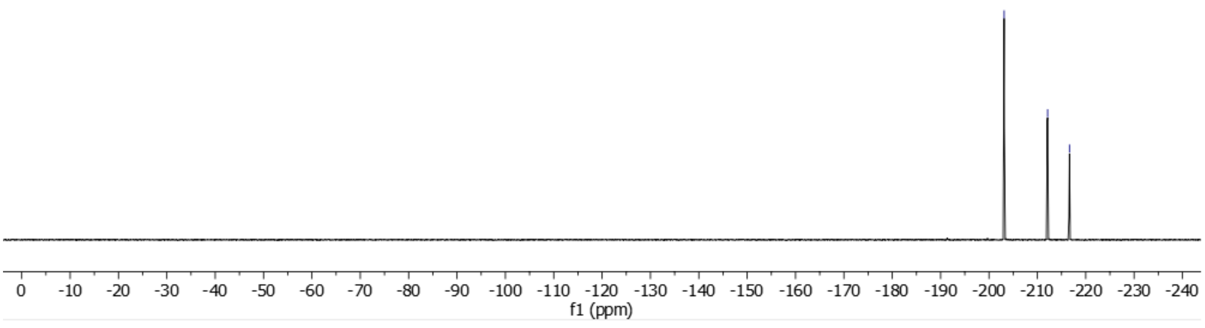

Supplement: SC-012-D1SC02130C-s001 [file SC-012-D1SC02130C-s001.pdf]
