# Supplementary material for: Supramolecular packing of alkyl substituted Janus face all-cis 2,3,4,5,6-pentafluorocyclohexyl motifs
Source: Chem Sci. 2021 Jun 4;12(28):9712–9. doi: 10.1039/d1sc02130c (PMC8293821; doi:10.1039/d1sc02130c)
Supplement: SC-012-D1SC02130C-s002 [file SC-012-D1SC02130C-s002.pdf]

Electronic Supplementary Material (ESI) for Chemical Science.
This journal is © The Royal Society of Chemistry 2021

Supplementary Information

Supramolecular packing of alkyl substituted Janus face all-*cis* 2,3,4,5,6-pentafluorocyclohexyl motifs

Joshua L. Clark, Alaric Taylor, Ailsa Geddis, Rifahath M. Neyyappadath, Bruno A. Piscelli, Cihang Yu, David B. Cordes, Alexandra M. Z. Slawin, Rodrigo A. Cormanich, Stefan Guldin, David O'Hagan

Index

**Synthetic protocols and compound characterisation**	**P2 - P 16**
**Computational Details**	**P17 – P24**
**Langmuir Isotherm Analysis set up**	**P25**
**X-Ray Crystallography Details**	**P26 - P27**

Synthetic protocols and compound characterisation

Synthesis of (cycloocta-1,5-dienyl)(2-(2,6-diisopropylphenyl)-3,3-dimethyl-2-azaspiro[4.5]decan-1-yl)rhodium chloride (2)

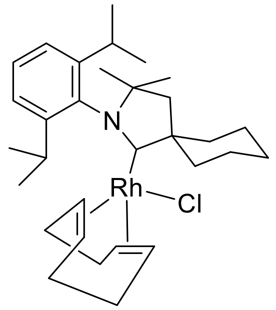

Rhodium-COD-chloride dimer (77 mg, 0.156 mmol, 0.5 eq), CAAC carbene chloride salt **X** (131 mg, 0.328 mmol, 1.05 eq) and KHMDS (150 mg, 0.750 mmol, 2.4 eq) were added to a schlenk tube inside an argon-filled glovebox. THF (10 mL) was added dropwise over 10 min at -78 °C. The resulting suspension was stirred for 10 min at -78 °C, and then warmed to room temperature and stirred for 16 h. The mixture was filtered and concentrated in vacuo to give the crude product, which was purified by flash column chromatography (19:1 pentane:Et_2O). The pure fractions were combined and concentrated in vacuo to give an oily residue. The residue was redissolved in DCM (1 mL) and **X** was precipitated by dropwise addition of pentane. The excess solvent was decanted leaving **X** as a yellow powder (97 mg, 54%): ^{1}H NMR ($CDCl_3$, 500 MHz) δ_H 7.46-7.38 (2H, m, ArH), 7.14 (1H, dd J = 7.5, 1,5, ArH), 5.24 (1H, t, J = 7.6), 4.60 (1H, q, J = 7.8), 3.94-3.85 (1H, m), 3.48-3.42 (1H, m), 2.92-2.84 (2H, m), 2.64- 2.45 (3H, m), 2.31-2.24 (1H, m), 2.20-2.09 (1H, m), 2.04-1.91 (3H, m), 1.79-1.72 (7H, m), 1.60-1.52 (2H, m), 1.50 (s, 3H), 1.46-1.30 (3H, m), 1.27-1.22 (9H, m), 1.20 (3H, s), 0.95 (3H, d, J = 6.7); ^{13}C NMR ($CDCl_3$, 125 MHz) δ_C 148.2, 146.4, 137.0, 129.0, 126.5, 124.1, 101.3 (d, J = 6.1 Hz), 98.2 (d, J = 5.8 Hz), 78.2, 71.9 (d, J = 15.0 Hz), 64.8, 64.6 (d, J = 13.9 Hz), 45.6, 41.8, 38.1, 35.0, 33.7, 30.9, 30.3, 28.9, 28.3, 28.0, 26.6, 26.4, 26.1, 25.9, 25.5, 24.6, 24.0, 22.6; data are in agreement with literature[1]

Synthesis of (*1R,2R,3s,4S,5S,6r*)-1,2,3,4,5-pentafluoro-6-methylcyclohexane (3)

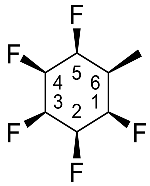

2,3,4,5,6-Pentafluoromethylbenzene (1 g, 5.49 mmol), silica gel (7 g), **2** (12 mg, 0.027 mmol, 0.5 mol%) and hexane (20 ml) was placed in a glass vial under under nitrogen. The autoclave was pressured with hydrogen (50 bar) and stirred for 24 h. After which the crude mixture was purified directly by flash column chromatography (SiO_2, CH_2Cl_2 in pentane) to give **3** (0.56 g, 3.0 mmol, 54 %) as a white crystalline solid; m.p. 182 °C; ^{1}H NMR (400 MHz, Acetone-d$_6$) δ_H 5.44-5.29 (1H, m, H-3), 5.04-4.75 (4H, overlapping m, H-1, H-2, H-4, H-5), 2.27- 2.05 (1H, m, H-6), 1.34 (3H, d J = 7.2 Hz, CH_3); ^{19}F NMR (377 MHz, Acetone-d$_6$) δ_F -205.1, -213.5, -217.5; ^{13}C NMR (101 MHz, Acetone) δ_C 90.2 (*C*F), 88.1 (*C*F) , 87.7 (*C*F), 33.8 (C-6), 12.1 (*C*H$_3$); HRMS m/z (ESI$^+$) (calculated $C_7H_9F_5Na^+$ = 211.0517) found 211.0515 [M+Na]$^+$

Synthesis of (*1r,2R,3R,4s,5S,6S*)-1-ethyl-2,3,4,5,6-pentafluorocyclohexane (4)

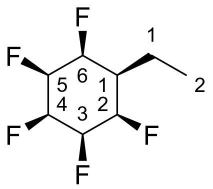

Activated 4 Å molecular sieves (5 g), 2,3,4,5,6-pentafluorostyrene (0.500 g, 2.58 mmol) and **2** (20 mg, 0.047 mmol, 2 mol%) were suspended in hexane (30 mL) in a vial and the vial placed inside an autoclave. The autoclave was pressurised with hydrogen to 50 Bar and the reaction mixture stirred at room temperature for 24 h. After depressurising and removing the vial, the suspension was filtered and concentrated in vacuo to give the crude product, which was purified by flash column chromatography (SiO_2, 10% EtOAc in hexane to 50% EtOAc in hexane) to give **4** as a white crystalline solid, (396 mg, 1.96 mmol, 76%); m.p. 148 °C; ^{1}H NMR (400 MHz, Acetone)

δ_H 5.47-5.30 (1H, m, FCH-4), 5.15-4.75 (4H, overlapping m, FCH-2, FCH-3, FCH-5, FCH-6), 1.92-1.77 (3H, overlapping m, FCCH-1, CH_2-1), 1.07 (3H, t J = 7.4 Hz, CH_3-2); ^{19}F NMR (377 MHz, Acetone) δ_F -204.9, -213.4, -217.5; ^{13}C NMR (176 MHz, Acetone) δ_C 89.1 (FC-4), 88.1 (FC-2, FC-6), 87.1 (FC-3, FC-5), 40.3 (FCC-1), 19.95 (CH$_2$-1), 11.4 (CH$_3$-2); HRMS m/z (ESI$^+$) (calculated $C_8H_{11}F_5Na^+$ = 225.0673) found 225.0669 $[M+Na]^+$.

Synthesis of (*E*)-1,2-bis(perfluorophenyl)ethene (11)

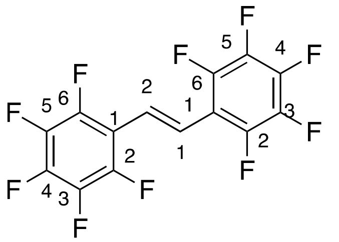

Zinc powder (0.75 g, 11.5 mmol, 2.25 eq) was added to a stirred suspension of $TiCl_4$ (0.6 mL, 5.5 mmol, 1.1 eq) in dry THF (25 mL), and the reaction stirred at 0 °C for 1 h. 2,3,4,5,6-Pentafluorobenzaldehyde (1.00 g, 5.1 mmol, 1 eq) was added dropwise over 10 mins and the reaction heated to reflux for 2 h. After cooling, water was added, and the mixture extracted into CH_2Cl_2. The combined extracts were washed with water, brine and dried over $MgSO_4$ and evaporated in vacuo. The residue was purified by flash column chromatography (SiO_2, petroleum ether) to give **11** as a white solid (173 mg, 19%); m.p. 91 °C; ^{1}H NMR (400.1 MHz, acetone-d6) δ_H: 7.36 (2H, s, CH=CH); ^{19}F{^{1}H} NMR (376.4 {400.1} MHz, acetone-d6) δ_F: -143.9 (4F), -156.5 (2F), -164.7 (4F), data are in accordance with the literature[2]

Synthesis of 1,2-bis((*1r,2R,3R,4S,5S,6S*)-2,3,4,5,6-pentafluorocyclohexyl)ethane (5)

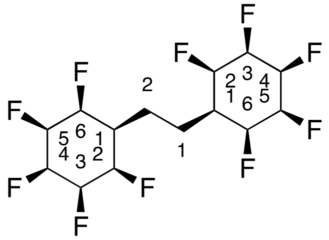

4 Å molecular sieves (2 g) were added to a solution of (E)-1,2-bis(perfluorophenyl)ethene (190 mg, 0.53 mmol, 1 equiv) and **2** (4 mg, 1 mol%) in dry hexane (8 mL) and the reaction was stirred for 72 h under 55 bar hydrogen pressure at 25 °C. Hydrogen ws carefully released from the autoclave and the reaction mass was filtered through celite, washed with acetone and then methanol to afford the title compound, 1,2-bis((1r,2R,3R,4S,5S,6S)-2,3,4,5,6-pentafluorocyclohexyl)ethane (56 mg, 28%); Mp: >250 °C; ^{1}H NMR (400 MHz, DMSO-d$_6$) δ_H 5.45-5.26 (2H, m, FCH-4), 5.11-4.68 (8H, overlapping m, FCH-2, FCH-3, FCH-5, FCH-6) 1.99-1.66 (6H, overlapping m, FCCH-1, CH$_2$-1, CH$_2$-2); ^{19}F NMR (376 MHz, DMSO-d$_6$) δ_F -203.3 (2F), -212.1 (2F), -216.4 (1F); HRMS (ESI$^+$) (calculated $C_{10}H_{16}F_{10}Na^+$ = 397.0990) found 397.0985 [M+Na]$^+$.

Synthesis of methyl (*E*)-11-(perfluorophenyl)undec-10-enoate (13)

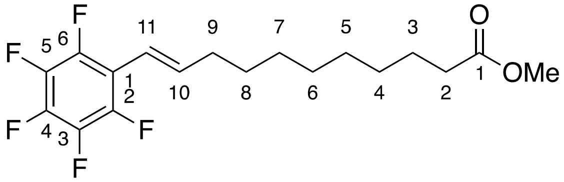

Methyl 10-undecenoate (198 mg, 1.00 mmol) and pentafluorostyrene (388 mg, 2.00 mmol) were added dropwise and simultaneously to a solution of 2nd Generation Hoveyda-Grubbs Catalyst® (12 mg, 0.019 mmol, 2 mol%) in CH_2Cl_2 (5 mL). The solution was heated to reflux for 14 h before being concentrated *in vacuo*. The residue was purified over silica eluting with hexane to 10% EtOAc in hexane) to give alkene **13** as a colourless oil, (195 mg, 0.536 mmol, 54%); ν_{max}/cm^{-1} 1740 (C=O), 1520 and 1495(C=C Ar); ^{1}H {^{19}F} NMR (500 MHz, CDCl$_3$): δ_H 6.55 (1H, dt *J* = 16.2, 7.1 Hz, H-10), 6.26 (1H, d *J* = 16.2 Hz, H-11), 3.68 (3H, s, OCH$_3$), 2.31 (2H, t *J* = 7.5 Hz, H-2), 2.29-2.24 (2H, m, H-9), 1.66-1.60 (2H, m, H-3), 1.51-1.45 (2H, m, H-8), 1.34-1.29 (8H, overlapping m, H-7, H-6, H-5, H-4); ^{13}C NMR (126 MHz, CDCl$_3$) δ_C 174.4 (C=O), 144.6 (Ar*C*F), 141.4 (C-10), 139.3 (Ar*C*F), 137.75 (Ar*C*F), 114.1 (C-11), 112.65 (ArC-1), 51.5 (O*C*H$_3$), 34.4 (C-9), 34.2 (C-2), 29.3 (*C*H$_2$), 29.3 (*C*H$_2$), 29.2 (*C*H$_2$), 29.2 (*C*H$_2$), 28.9 (*C*H$_2$), 25.0 (C-3); ^{19}F {^{1}H}

NMR (470 MHz, $CDCl_3$) δ_F -144.0 (ArCF), -158.2 (F-4), -163.6 (ArCF); HRMS m/z (ESI^+) (calculated $C_{18}H_{22}F_5O_2^+$ = 365.1534) found 365.1530 $[M+H]^+$.

Synthesis of (*E*)-11-(perfluorophenyl)undec-10-enal (14)

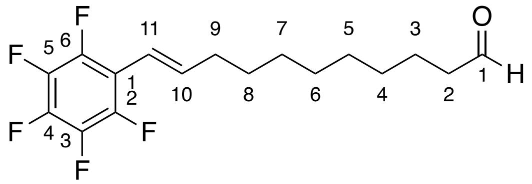

DIBAl-H (1.1 mL, 1M in hexane, 1.1 mmol) was added dropwise to a solution of **13** (130 mg, 0.357 mmol) in CH_2Cl_2 (9 mL) at -78 °C. The resulting solution was stirred for 40 min at -78 °C before being quenched by the careful addition of MeOH (1 mL). After stirring for 10 min, the solution was warmed to r.t. and saturated potassium sodium tartarate (Rochelle salt solution (10 mL) was added. The biphasic mixture was stirred at r.t. for 12 h and then extracted with CH_2Cl_2 (3 x 10 mL). The combined organic phase was dried over $MgSO_4$, filtered and concentrated *in vacuo* to give **14** as a colourless oil, (119 mg, 0.357 mmol, quantitative); ν_{max} cm^{-1} 2160 (C=C), 1734 (C=O), 1047 (C-F); ^1H NMR (700 MHz, $CDCl_3$) δ_H 9.76 (1H, t J = 1.8 Hz, H-1), 6.53 (1H, dt J = 16.2, 7.0 Hz, H-10), 6.24 (1H, dt J = 16.2, 1.5 Hz, H-11), 2.42 (2H, td J = 7.4, 1.8 Hz, H-2), 2.26-2.23 (2H, m, H-9), 1.63-1.60 (2H, m, H-3), 1.49-1.45 (2H, m H-8), 1.33-1.28 (8H, overlapping m, H-7, H-6, H-5, H-4); ^{13}C NMR (101 MHz, $CDCl_3$) δ_C 203.0 (C=O), 144.6 (Ar*C*F), 141.4 (C-10), 139.5 (Ar*C*F), 137.7 (Ar*C*F), 114.2 (C-11), 112.65 (ArC-1), 44.0 (C-2), 34.4 (C-9), 29.4 (CH_2), 29.3 (CH_2), 29.2 (CH_2), 29.2 (CH_2), 28.9 (CH_2), 22.2 (C-3); ^{19}F NMR (377 MHz, $CDCl_3$) δ_F -143.95 (ArCF), -158.1 (F-4), -163.6 (ArCF); HRMS m/z (EI^+) (calculated $C_{17}H_{19}F_5O^+$ = 334.1351) found 334.1351 $[M-e^-]^+$.

Synthesis of diethyl (perfluorophenyl)methyl)phosphonate (15)

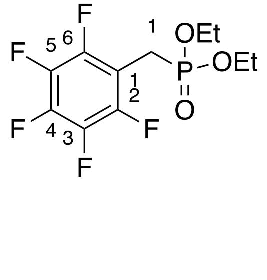

A microwave vial was charged with pentafluorobenzyl bromide (522 mg, 2.00 mmol) and triethyl phosphite (332 mg, 2.00 mmol) and then sealed using a Teflon cap. The vial was irradiated at 140 °C for 5 min and then traces of starting material was removed *in vacuo* to give **15** as a colourless oil, (631, 1.98 mmol, 99%); ν_{max}/cm^{-1} 2984 (C-H), 1522 and 1506 (C=C Ar), 1269 (C-F); ^{1}H NMR (300 MHz, $CDCl_3$) δ_H 4.14-4.02 (4H, m, OC*H*$_2$CH$_3$), 3.21 (1H, t *J* = 1.6 Hz, PCH-1a), 3.14 (1H, t *J* = 1.6 Hz, PCH-1b) 1.26 (6H, t *J* = 7.1 Hz, OCH$_2$C*H*$_3$); ^{13}C NMR (126 MHz, $CDCl_3$) δ_C 145.15 (Ar*C*F), 140.4 (Ar*C*F), 137.7 (Ar*C*F), 107.0 (Ar), 62.8 (O*C*H$_2$CH$_3$), 21.7 (PC-1b), 20.6 (PC-1a), 16.4 (OCH$_2$*C*H$_3$); ^{31}P NMR (162 MHz, $CDCl_3$) δ_P 21.63; ^{19}F NMR (376 MHz, $CDCl_3$) δ_F -141.4 (ArCF), -155.8 (F-4), -162.3 (ArCF); HRMS m/z (ESI$^+$) (calculated $C_{11}H_{12}O_3F_5NaP^+$ = 341.0336) found 341.0327 [M+Na]$^+$.

Synthesis of (*1E,11E*)-1,12-bis(perfluorophenyl)dodeca-1,11-diene (16)

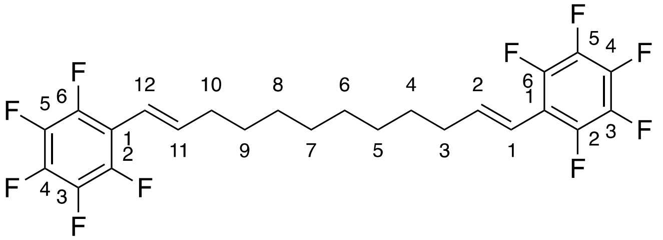

NaH (60% in oil, 58 mg, 1.46 mmol) was added to a solution of **15** (230 mg, 0.724 mmol) in THF (2.4 mL) at 0 °C. The suspension was stirred at 0 °C for 5 min before **14** (114 mg, 0.341 mmol) was added dropwise as a solution in THF (3 mL). The reaction was heated at 76 °C for 14 h. The solution was cooled to 0 °C and the reaction quenched by the careful addition of water (5 mL). The mixture was extracted into EtOAc (3 x 20 mL) and the combined organic phase was washed with saturated brine. The organic phase was dried over $MgSO_4$, filtered and concentrated *in vacuo*. The residue was purified by flash column chromatography (SiO_2, hexane) to give **16** as a colourless oil, (92 mg, 0.18 mmol, 53%); ν_{max} cm^{-1} 1520 and 1493 (C=C Ar); ^{1}H NMR (700 MHz, $CDCl_3$) δ_H 6.55 (2H, dt *J* = 16.2, 7.1 Hz, H-11, H-2), 6.26 (2H, dt *J* = 16.2, 1.3 Hz, H-1, H-12), 2.28-2.24 (4H, m, H-10, H-3), 1.51-1.46 (4H, m, CH_2), 1.38-1.31 (8H, overlapping m, CH_2); ^{19}F NMR (471 MHz, $CDCl_3$) δ -144.0 (ArCF), -158.1 (F-4), -163.6 (ArCF); ^{13}C NMR (176 MHz, $CDCl_3$) δ_C

144.6 (Ar*C*F), 141.4 (H_2C-11, H_2C-2), 139.4 (Ar*C*F), 137.8 (Ar*C*F), 114.2 (H_2C-12, H_2C-1), 112.7 (ArC-1), 34.45 (H_2C-10, H_2C-3), 29.5 (H_2C), 29.3 (H_2C), 29.0 (H_2C); HRMS m/z (EI^+) (calculated $C_{24}H_{20}F_{10}^+$ = 498.1400) found 498.1397 $[M\text{-}e^-]^+$.

Synthesis of 1,12-bis((*1r,2R,3R,4s,5S,6S*)-2,3,4,5,6-pentafluorocyclohexyl)dodecane (6)

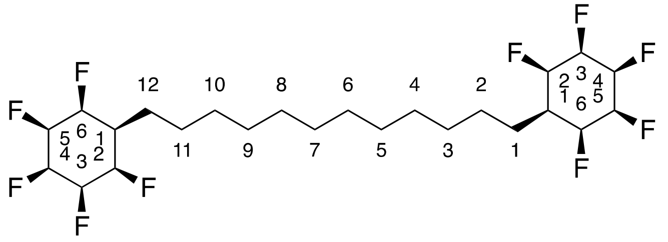

Activated 4 Å molecular sieves (500 mg), **16** (50 mg, 0.10 mmol) and **2** (2 mg, 0.005 mmol, 5 mol%) were suspended in hexane (2 mL) in a vial and the vial placed inside an autoclave. The autoclave was pressurised with hydrogen to 50 Bar and the reaction mixture stirred at room temperature for 24 h. After depressurising and removing the vial, the suspension was filtered and concentrated in vacuo to give the product, which was purified by flash column chromatography (SiO_2, 20% EtOAc in hexane to 100% EtOAc) to give **6** as a white crystalline solid, (17 mg, 0.033 mmol, 33%); m.p. 188 °C; v_{max}/cm^{-1} 1132 and 1049 (C-F); ^1H NMR (500 MHz, $CDCl_3$) δ_H 5.43-5.26 (2H, m, FCH-4), 5.03-4.88 (4H, m, FCH-2, FCH-6), 4.56-4.34 (4H, m, FCH-3, FCH-5), 1.90-1.85 (4H, m, CH_2-1, CH_2-12), 1.74-1.56 (2H, m, FCHCH-1), 1.49-1.43 (4H, m, CH_2-11, CH_2-2), 1.39-1.28 (16H, overlapping m, CH_2); ^{19}F NMR (471 MHz, Acetone) δ_F -204.8, -213.05, -217.45 (F-4); ^{13}C NMR (126 MHz, $CDCl_3$) δ_C 87.3 (FC-2, FC-6), 87.1 (FC-4), 86.5 (FC-3, FC-5), 38.6 (FC*C*-1), 32.1 (H_2C), 29.8 (H_2C), 29.7 (H_2C), 29.6 (H_2C), 26.6 (H_2C-11, H_2C-2), 26.0 (H_2C-1, H_2C-12); HRMS m/z (ESI^+) (calculated $C_{24}H_{36}F_{10}Na^+$ = 537.2550) found 537.2540 $[M+Na]^+$.

Synthesis of methyl 13-phenyltridec-10-enoate (19)

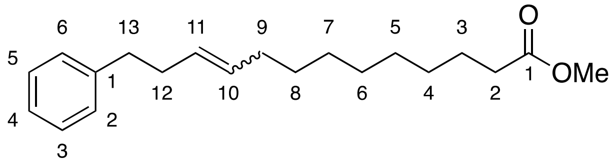

Methyl 10-undecenoate (1.051 g, 5.300 mmol) and 4-phenylbutene (0.700 g, 5.30 mmol) were simultaneously added dropwise to a solution Grubbs first generation Catalyst® (0.217 g, 0.264 mmol, 5 mol%) in CH_2Cl_2 (12 mL). The solution was then heated to reflux for 14 h before being concentrated *in vacuo* to give **19** as a colourless oil as a mixture of diastereomers, which were not separated prior to the next step, (0.626 g, 2.07 mmol, 39%); ν_{max}/cm^{-1} 1740 (C=O); ^{1}H NMR (500 MHz, $CDCl_3$) δ_H 7.31-7.16 (5H, overlapping m, ArH), 5.50-5.37 (2H, m, H-10, H-11), 3.67 (3H, s, OCH_3), 2.70-2.65 (2H, m, CH_2), 2.35-2.29 (4H, overlapping m, CH_2), 1.99-1.95 (2H, m, CH_2), 1.66-1.59 (2H, m, CH_2), 1.35-1.24 (10H, overlapping m, CH_2); ^{13}C NMR (126 MHz, $CDCl_3$) δ_C 174.45 (C=O), 142.3 (ArC-1), 131.2 (C=C), 130.7 (C=C), 129.4 (C=C), 128.8 (C=C), 128.6 (Ar*C*H), 128.3 (Ar*C*H), 125.8 (Ar*C*H), 51.6 (O*C*H_3), 36.3 (*C*H_2), 34.6 (*C*H_2), 34.2 (*C*H_2), 32.65 (*C*H_2), 29.6 (*C*H_2), 29.4 (*C*H_2), 29.3 (*C*H_2), 29.3 (*C*H_2), 29.1 (*C*H_2), 25.1 (*C*H_2); HRMS m/z (ESI^+) (calculated $C_{20}H_{30}O_2Na^+$ = 325.2138) found 325.2133 $[M+Na]^+$.

Synthesis of methyl 13-cyclohexyltridecanoate (20)

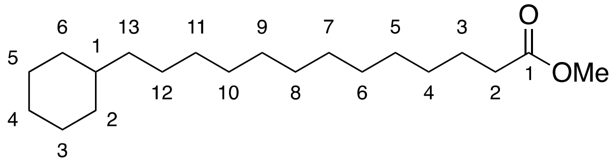

Activated 4 Å molecular sieves (500 mg), **19** (54 mg, 0.18 mmol) and **2** (6 mg, 0.015 mmol, 8 mol%) were suspended in hexane (2 mL) in a vial and the vial placed inside an autoclave. The autoclave was pressurised with hydrogen to 50 Bar and the reaction mixture stirred at room temperature for 24 h. After depressurising and removing the vial, the suspension was filtered and concentrated in vacuo to give the crude product, which was purified by flash column

chromatography (SiO_2, hexane) to give **20** as a colourless oil, (41 mg, 0.132 mmol, 74%); ν_{max}/cm^{-1} 1742 (C=O); ^{1}H NMR (500 MHz, $CDCl_3$) δ_H 3.66 (3H, s, OCH_3), 2.29 (2H, t *J* = 7.6 Hz, $OCCH_2$-2), 1.71-1.57 (7H, overlapping m, CH_2), 1.32-1.10 (24H, overlapping m, CH_2), 0.89-0.79 (2H, m, CH_2); ^{13}C NMR (126 MHz, $CDCl_3$) δ 174.5 (C=O), 51.6 (O*C*H_3) , 37.8 (CH_2), 37.7 (CH_2), 34.3 (OC*C*H_2-2), 33.6 (CH_2), 30.15 (CH_2), 29.9 (CH_2), 29.8 (CH_2), 29.8 (CH_2), 29.7 (CH_2), 29.6 (CH_2), 29.4 (CH_2), 29.3 (CH_2), 27.0 (CH_2), 26.9 (CH_2), 26.6 (CH_2), 25.1 (CH_2); HRMS m/z (ESI$^+$) (calculated $C_{20}H_{38}O_2Na^+$ = 333.2764) found 333.2758 $[M+Na]^+$.

Synthesis of 13-cyclohexyltridecanoic acid (21)

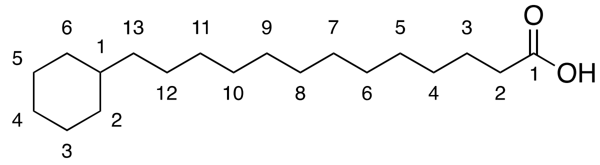

NaOH solution (1M, 4 mL, 4 mmol) was added to a solution of **20** (43 mg, 0.14 mmol) in MeOH (10 mL). The solution was stirred at reflux for 14 h before being acidified by the careful addition of HCl (1M, 30 mL). The mixture was extracted into CH_2Cl_2 (3 x 50 mL) before being dried over $MgSO_4$, filtered and concentrated *in vacuo* to give **21** as a crystalline white solid, (41 mg, 0.14 mmol, 100%); m.p. 65 °C; ν_{max}/cm^{-1} 2914 (CO-H), 1699 (C=O); ^{1}H NMR (500 MHz, $CDCl_3$) δ_H 2.34 (2H, t *J* = 7.6 Hz, $OCCH_2$-2), 1.78-1.61 (7H, overlapping m, CH_2), 1.33-1.12 (24H, overlapping m, CH_2), 0.90-0.81 (2H, m, CH_2); ^{13}C NMR (126 MHz, $CDCl_3$) δ_C 180.6 (C=O), 37.85 (*C*H_2), 37.7 (*C*H_2), 34.3 (OC*C*H_2-2), 33.6 (*C*H_2), 30.2 (*C*H_2), 29.9 (*C*H_2), 29.8 (*C*H_2), 29.8 (*C*H_2), 29.75 (*C*H_2), 29.6 (*C*H_2), 29.4 (*C*H_2), 29.2 (*C*H_2), 27.0 (*C*H_2), 26.9 (*C*H_2), 26.6 (*C*H_2), 24.8 (*C*H_2); HRMS m/z (ESI$^-$) (calculated $C_{19}H_{35}O_2^-$ = 295.2643) found 295.2637 $[M-H]^+$

Synthesis of 13-cyclohexyltridecan-1-ol (22)

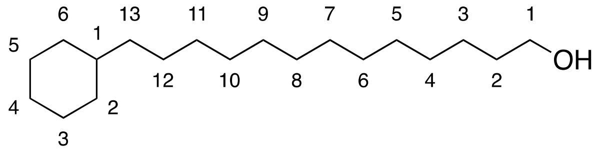

DIBAL-H (1M in hexane, 1.05 mL, 1.05 mmol) was added to a solution of **21** (0.156 g, 0.419 mmol) in CH_2Cl_2 (3 mL) at -78 °C. The solution was warmed to r.t. and stirred for 1 h before being diluted with Et_2O and cooled to 0 °C. The solution was then quenched by the sequential addition of water (0.04 mL), NaOH (15% w/w, 0.04 mL) and then water (0.1 mL). The solution was then warmed to r.t. and stirred for 15 min before being dried over $MgSO_4$ and the suspension stirred for a further 15 min. The suspension was filtered and concentrated *in vacuo* to give **22** as a white amorphous solid (0.096 g, 0.34 mmol, 81%); ν_{max}/cm^{-1} 3447 (O-H), 1049 (C-OH); ^1H NMR (500 MHz, $CDCl_3$) δ_H 3.64 (2H, t *J* = 6.7 Hz, OCH_2-1), 1.76-1.62 (6H, m, CH_2), 1.60-1.54 (2H, m, CH_2), 1.38-1.14 (26H, m, CH_2), 0.91-0.83 (2H, m, CH_2); ^{13}C NMR (126 MHz, $CDCl_3$) δ_C 63.0 (O*C*H_2-1), 37.7 (*C*H_2), 37.6 (*C*H_2), 33.5 (*C*H_2), 32.8 (*C*H_2), 30.0 (*C*H_2), 29.7 (*C*H_2), 29.7 (*C*H_2), 29.7 (*C*H_2), 29.6 (*C*H_2), 29.5 (*C*H_2), 26.9 (*C*H_2), 26.8 (*C*H_2), 26.5 (*C*H_2), 25.8 (*C*H_2); HRMS m/z (ESI^+) (calculated $C_{19}H_{38}ONa^+$ = 305.2815) found 305.2798 $[M+Na]^+$.

Synthesis of methyl 12-hydroxydodecanoate (23)

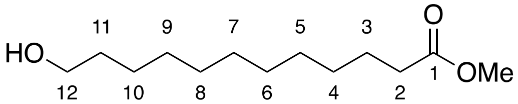

Acetyl chloride (2.14 mL, 30 mmol) was added dropwise to a solution of 12-hydroxydodecanoic acid (4.059 g, 18.78 mmol) in MeOH (60 mL). The solution was heated to reflux for 14 h before being neutralised by careful addition of NaOH to pH 7. Excess MeOH was removed *in vacuo* and the residue was extracted into EtOAc (3 x 50 mL). The organic phase was dried over $MgSO_4$, filtered and concentrated *in vacuo* to give **23** as a colourless oil which was used without further purification, (4.105 g, 95%); ν_{max}/cm^{-1} 3400br (O-H), 1738 (C=O); ^1H NMR (500 MHz, $CDCl_3$)

δ_H 3.66 (3H, s, OCH_3), 3.63 (2H, t *J* = 6.5 Hz, H-12), 2.30 (2H, t *J* = 7.5 Hz, H-2), 1.65-1.52 (5H, overlapping m, CH_2, OH), 1.35-1.25 (14H, overlapping m, CH_2); ^{13}C NMR (126 MHz, $CDCl_3$) δ_C 174.5 (C=O), 63.2 (C-12), 51.6 (O*C*H_3), 34.25 (C-2), 32.9 (CH_2), 29.7 (CH_2), 29.6 (CH_2), 29.5 (CH_2), 29.4 (CH_2), 29.3 (CH_2), 25.85 (CH_2), 25.1 (CH_2); HRMS m/z (ESI$^+$) (calculated $C_{13}H_{26}O_3Na^+$ = 253.1774) found 253.1768 $[M+Na]^+$.

Synthesis of methyl 12-oxododecanoate (24)

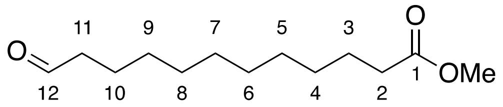

DMSO (2.6 mL, 37 mmol) was as added to a solution of $(COCl)_2$ (2.31 g, 18.2 mmol) in CH_2Cl_2 CH_2Cl_2 (52 mL) at -78 °C. The solution was stirred for 30 mins at -78 °C before **23** (3.82 g, 16.6 mmol) in CH_2Cl_2 (17.6 mL) was added dropwise. The solution was stirred at -60 °C for 45 mins before triethylamine (11.6 mL, 83.2 mmol) was added and it was then warmed to r.t.. The solution was stirred for 1 h at r.t. before being diluted with water (100 mL) and then extracted into CH_2Cl_2 (2 x 100 mL). The organic phase was washed with brine, dried over $MgSO_4$, filtered and concentrated *in vacuo*. The residue was purified by flash column chromatography (SiO_2, hexane to 10% EtOAc in hexane) to give **24** as a colourless oil, (2.84 g, 12.3 mmol, 74%); ν_{max}/cm^{-1} 1736 (C=O); ^{1}H NMR (500 MHz, $CDCl_3$) δ_H 9.75 (1H, s, H-12), 3.66 (OCH_3), 2.41 (2H, t *J* = 7.1 Hz, H-11), 2.29 (2H, t *J* = 7.5 Hz, H-2), 1.63-1.59 (4H, overlapping m, CH_2), 1.33-1.23 (12H, overlapping m, CH_2); ^{13}C NMR (126 MHz, $CDCl_3$) δ_C 203.1 (C-12), 174.5 (C-1), 51.6 (O*C*H_3), 44.0 (C-11), 34.2 (C-2), 29.5 (CH_2), 29.5 (CH_2), 29.5 (CH_2), 29.3 (CH_2), 29.3 (CH_2), 29.3 (CH_2), 25.1 (CH_2), 22.2 (CH_2), data are in agreement with literature.[3]

Synthesis of methyl *E*-13-(perfluorophenyl)tridec-12-enoate (27)

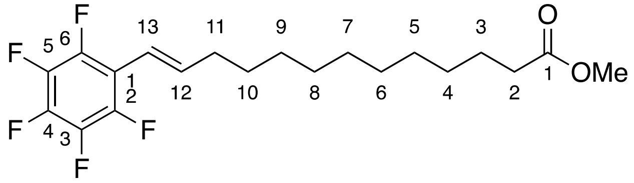

NaH (60% in oil, 0.350 g, 8.76 mmol) was added to a solution of **15** (1.394 g, 4.381 mmol) in THF (12 mL) at 0 °C. The suspension was stirred for 5 mins at 0 °C before **24** (1.00 g, 4.38 mmol) was added dropwise. The solution was heated to 50 °C for 14 h before being quenched by the careful addition of water (5 mL) at 0 °C. The mixture was extracted into EtOAc (3 x 20 mL) and the combined organic phase was washed with saturated brine. The organic phase was dried over $MgSO_4$, filtered and concentrated *in vacuo*. The residue was purified by flash column chromatography (SiO_2, hexane to 50% EtOAc in hexane) to give **27** as a colourless oil (0.995 g, 4.36 mmol, 58%) which was a mixture of E:Z (5:1) diastereomers which were not separated (only peaks of the major isomer are assigned); ν_{max}/cm^{-1} 1740 (C=O); ^1H NMR (500 MHz, $CDCl_3$) δ_H 6.54 (1H, dt *J* = 16.2, 7.1 Hz, H-12), 6.25 (1H, d *J* = 16.2, H-13), 3.66 (3H, s, OCH_3), 2.30 (2H, t *J* = 7.5 Hz, H-2), 2.25 (2H, app q *J* = 7.1 Hz, H-11), 1.64-1.58 (2H, m, CH_2), 1.50-1.45 (2H, m, CH_2), 1.36-1.26 (12 H, overlapping m, CH_2); ^{13}C NMR (126 MHz, $CDCl_3$) δ_C 174.5 (C=O), 144.7 (C-F), 141.5 (C-12), 139.6 (C-F), 137.4 (C-F), 114.1 (C-13), 112.7 (ArC-1), 51.6 (O*C*H_3), 34.45 (C-11), 34.25 (C-2), 29.6 (CH_2), 29.6 (CH_2), 29.5 (CH_2), 29.5 (CH_2), 29.4 (CH_2), 29.3 (CH_2), 29.0 (CH_2), 25.1 (CH_2); ^{19}F NMR (471 MHz, $CDCl_3$) δ_F -143.9 (ArCF), -158.1 (F-4), -163.5 (ArCF); HRMS m/z (ESI^+) (calculated $C_{20}H_{25}O_2F_5Na^+$ = 415.1667) found 415.1657 $[M+Na]^+$.

Synthesis of methyl 13-(*1r,2R,3R,4s,5S,6S*)-2,3,4,5,6-pentafluorocyclohexyl)tridecanoate (28)

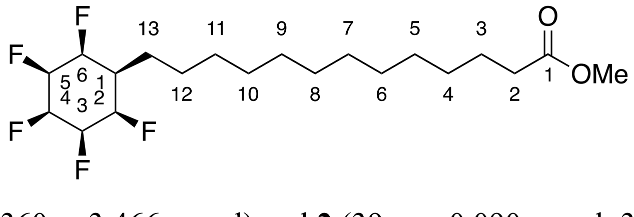

Silica gel (8.5 g), **27** (1.360 g, 3.466 mmol) and **2** (39 mg, 0.090 mmol, 3 mol%) were suspended in hexane (50 mL) in a vial and the vial placed inside an autoclave. The autoclave was pressurised with hydrogen to 50 Bar and the reaction mixture stirred at room temperature for 24 h. After depressurising and removing the vial, the suspension was filtered and concentrated in vacuo to give the crude product, which was purified by flash column chromatography (SiO_2, 50% EtOAc

in hexane to 100% EtOAc) to give **28** as a white crystalline solid, (0.300 g, 0.749 mmol, 22%); m.p. 112 °C; ν_{max}/cm^{-1} 1734 (C=O), 1047 (C-F); ^{1}H NMR (500 MHz, $CDCl_3$) δ_H 5.41-5.25 (1H, m, FCH-4), 5.03-4.86 (2H, m, FCH-2, FCH-6), 4.54-4.32 (2H, m, FCH-3, FCH-6), 3.66 (3H, s, OCH_3), 2.30 (2H, t, J = 7.6 Hz, CH_2-2), 1.88-1.83 (2H, m, CH_2), 1.72-1.48 (5H, overlapping m, FCCH-1, CH_2-3, CH_2), 1.46-1.41 (2H, m, CH_2), 1.35-1.25 (16H, overlapping m, CH_2); ^{19}F NMR (471 MHz, $CDCl_3$) δ_F -203.1, -212.1, -216.7 (F-4); ^{13}C NMR (126 MHz, $CDCl_3$) δ_C 174.5 (C=O), 87.4 (FC-2, FC-6), 87.3 (FC-4), 86.4 (FC-3, FC-5), 51.6 (O*C*H_3), 38.6 (FC*C*-1), 34.3 (*C*H_2-2), 29.7 (*C*H_2), 29.7 (*C*H_2), 29.6 (*C*H_2), 29.6 (*C*H_2), 29.5 (*C*H_2), 29.4 (*C*H_2), 29.3 (*C*H_2), 26.8 (*C*H_2), 26.6 (*C*H_2), 26.0 (*C*H_2), 25.1 (*C*H_2); HRMS m/z (ESI$^+$) (calculated $C_{20}H_{33}O_2F_5Na^+$ = 423.2293) found 423.2286 $[M+Na]^+$.

Synthesis of 13-(*1r,2R,3R,4s,5S,6S*)-2,3,4,5,6-pentafluorocyclohexyl)tridecanoic acid (7)

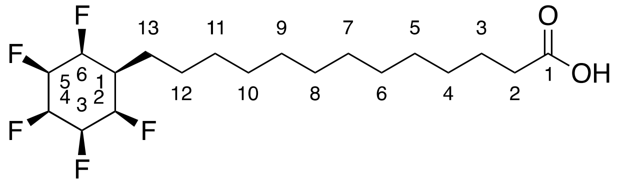

A suspension of **28** (90 mg, 0.22 mmol) and HCl (6N, 100 mL) was heated to reflux and stirred for 14 h before being cooled to 0 °C. The reaction was basified by careful addition of saturated $NaHCO_3$ solution. Residual starting material was extracted into EtOAc (3 x 200 mL) and the aqueous phase was re-acidified by addition of HCL (1M). The re-acidified solution was extracted into EtOAc (3 x 200 mL) and this organic phase was dried over $MgSO_4$, filtered and concentrated *in vacuo* to give **7** as a white crystalline solid, (81 mg, 0.21 mmol, 95%); m.p. 178 °C; ν_{max}/cm^{-1} 1701 (C=O), 1126 and 1049 (C-F); ^{1}H NMR (500 MHz, Acetone) δ_H 10.40 (COOH), 5.46-5.32 (1H, m, FCH-4), 5.12-4.95 (2H, m, FCH-2, FCH-6) 4.97-4.77 (2H, m, FCH-3, FCH-5), 2.27 (2H, t J = 7.5 Hz, CH_2-2), 2.06-1.90 (1H, m, FCCH-1), 1.83-1.78 (2H, m, CH_2), 1.61-1.55 (2H, m, CH_2), 1.52-1.46 (2H, m, CH_2), 1.37-1.29 (16H, overlapping m, CH_2); ^{19}F NMR (471 MHz, Acetone) δ_F -204.8, -213.0, -217.5 (F-4); ^{13}C NMR (126 MHz, Acetone) δ_C 174.7 (C=O), 88.95 (FC-2, FC-6), 88.75 (FC-4), 87.6 (FC-3, FC-5), 38.6 (FC*C*-1), 34.2 (*C*H_2-2), 30.4 (*C*H_2), 30.4 (*C*H_2), 30.4 (*C*H_2), 30.3 (*C*H_2), 30.2 (*C*H_2), 30.1 (*C*H_2), 30.1 (*C*H_2), 30.0 (*C*H_2), 27.1 (*C*H_2), 26.7

(CH_2), 25.7 (CH_2); HRMS m/z (ESI$^+$) (calculated $C_{19}H_{31}O_2F_5Na^+$ = 409.2136) found 409.2132 [M+Na]$^+$.

Synthesis of 13-(*1r,2R,3R,4s,5S,6S*)-2,3,4,5,6-pentafluorocyclohexyl)tridecan-1-ol (8)

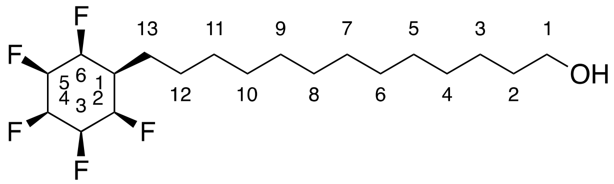

Diisobutylaluminium hydride solution (1M in hexane, 0.49 mL, 0.49 mmol) was added to a solution of **28** (79 mg, 0.197 mmol) in CH_2Cl_2 (1.5 mL) at -78 °C. The reaction was warmed to r.t. and stirred for a further 1 h. The reaction was diluted with Et_2O (10 mL), cooled to 0 °C and quenched by the sequential addition of water (0.02 mL), NaOH (15% w/w, 0.02 mL) and water (0.05 mL). The mixture was warmed to r.t and $MgSO_4$ was added. The suspension was stirred for 15 mins before being filtered and concentrated *in vacuo* to give the crude product, which was purified by flash column chromatography (SiO_2, 80% EtOAc in hexane to 100% EtOAc) to give **8** as a white crystalline solid, (47 mg, 0.126 mmol, 64%); m.p. 140 °C; v_{max}/cm^{-1} 3325 (O-H), 1126 and 1049 (C-F); ^{1}H NMR (500 MHz, Acetone) δ_H 5.45-5.30 (1H, m, FCH-4), 5.13-4.72 (4H, overlapping m, FCH-2, FCH-3, FCH-5, FCH-6) 3.54-3.50 (2H, m, CH_2-1), 3.37 (1H, t J = 5.2 Hz, OH), 2.08-1.90 (1H, m, FCCH-1), 1.83-1.78 (2H, m, CH_2), 1.53-1.45 (4H, overlapping m, CH_2), 1.35-1.29 (18H, overlapping m, CH_2); ^{19}F NMR (470 MHz, Acetone) δ_F -204.8, -213.05, -217.4; ^{13}C NMR (126 MHz, Acetone) δ_C 89.4 (FC-2, FC-6), 88.8 (FC-4), 87.6 (FC-3, FC-5), 62.5 (CH_2OH), 38.6 (FCC-1), 33.9 (H_2C), 30.5 (CH_2), 30.5 (CH_2), 30.4 (CH_2), 30.4 (CH_2), 30.4 (CH_2), 30.3 (CH_2), 30.3 (CH_2), 30.2 (CH_2), 27.3 (CH_2), 27.1 (CH_2), 26.8 (CH_2); HRMS m/z (ESI$^+$) (calculated $C_{19}H_{33}OF_5Na^+$ = 395.2344) found 395.2343 [M+Na]$^+$.

(*1R,2R,3s,4S,5S,6r*)-1,2,3,4,5-pentafluoro-6-tridecylcyclohexane 9

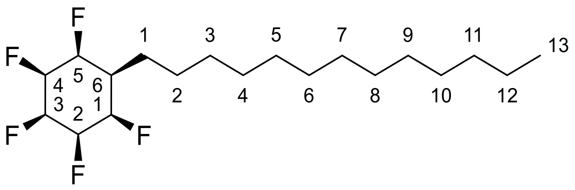

1-Allyl-2,3,4,5,6-pentafluorobenzene (0.306 mL, 2.00 mmol) and 1-dodecene (0.444 mL, 2.00 mmol) were added dropwise and simultaneously to a solution of Grubbs first generation catalyst® (82 mg, 5 mol%) in CH_2Cl_2 (6 mL). The solution was heated to 40 °C for 14 h and was then concentrated *in vacuo* and the residue was filtered through a plug of silica. The silica was washed with hexane. The filtrate was concentrated *in vacuo* to give the product **30** which was not purified prior to the next step. The product (530 mg), activated 4 Å molecular sieves (5.3 g) Silica gel (8.5 g) and **2** (25 mg, 0.058 mmol) were suspended in hexane (20 mL) in a vial and the vial placed inside an autoclave. The autoclave was pressurised with hydrogen to 50 Bar and the reaction mixture stirred at room temperature for 24 h. After depressurising and removing the vial, the suspension was filtered and concentrated in vacuo to give the product, which was purified by flash column chromatography (SiO_2, 20% EtOAc in hexane to 40% EtOAc) to give **9** as a white crystalline solid, (228 mg, 0.640 mmol, 32% over two steps); m.p. 121 °C; ^1H NMR (400 MHz, $CDCl_3$) δ_H 5.43-5.23 (1H, m, FCH-3), 5.04-4.83 (2H, m, FCH-1, FCH-5), 4.55-4.28 (2H, m, FCH-2, FCH-4), 1.89-1.82 (2H, m, CH_2), 1.66-1.51 (1H, m, FCCH-6), 1.48-1.39 (2H, m, CH_2), 1.33-1.25 (20H, overlapping m, CH_2), 0.88 (3H, t J = 6.9 Hz, CH_3-13); ^{13}C NMR (101 MHz, $CDCl_3$) δ_C 87.4 (FC-1, FC-5), 87.3 (FC-3), 86.4 (FC-2, FC-4), 38.7 (FCC-6), 32.1 (CH_2), 29.8 (CH_2), 29.8 (CH_2), 29.8 (CH_2), 29.7 (CH_2), 29.6 (CH_2), 29.6 (CH_2), 29.5 (CH_2), 26.7 (CH_2), 26.0 (CH_2), 26.0 (CH_2), 22.8 (CH_2), 14.3 (CH_3-13); ^{19}F NMR (376 MHz, $CDCl_3$) δ_F -203.1, -212.1, -216.6; HRMS m/z (ESI$^+$) (calculated $C_{19}H_{33}F_5Na^+$ = 379.2395) found 379.2390 $[M+Na]^+$.

References

1. M. P. Wiesenfeldt, Z. Nairoukh, W. Lei and F. Glorius, *Science*, 2017, **357**, 908-912
2. V. Rauniyar, H. Zhai, and D. G. Hall, *J. Am. Chem. Soc.*, 2008, **130**, 8481-8490
3. J. Ternel, J. Couturier, J. Dubois, J. Carpentier, *Adv. Synth. Catal.*, 2013, **355**, 3191-3204

Computational Details

Compounds **1**, **3** and **4** were optimized at the M06-2X/aug-cc-pVTZ theoretical level and harmonic frequency calculations were carried out at the same level in order to identify each conformer as true energy minima, showing no imaginary frequencies using the Gaussian16 Rev C.01 program.[1] Single point energies using the DLPNO approximation for CCSD(T) implemented in ORCA 4.2.1 and def2-TZVP basis set were calculated over the optimized geometry and considered as reference for a DFT benchmark study (Figure S1). The M06-L Minnesota meta-GGA functional with GD3 empirical dispersion correction showed the lowest MAE (Mean Absolute Error) with respect to DLPNO-CCSD(T), and thus was chosen for the subsequent calculations. Compounds **1**, **3** and **4** were reoptimized at the M06L-D3/aug-cc-pVTZ theoretical level and harmonic frequency calculations were carried out at the same level to confirm each conformer as true energy minima, showing no imaginary frequencies. The same harmonic frequency calculations were used to obtain Gibbs free energies at standard pressure and temperature for gas-phase calculations. NBO calculations, including NPA, NCE and NSA analysis were done over the M06L-D3/aug-cc-pVTZ molecular density functions using the NBO 7.0 program.[2] The total molecular dipole calculated at the M06L-D3/aug-cc-pVTZ theoretical level was decomposed in each NLMO contribution using the DIPOLE analysis from NBO 7.0.

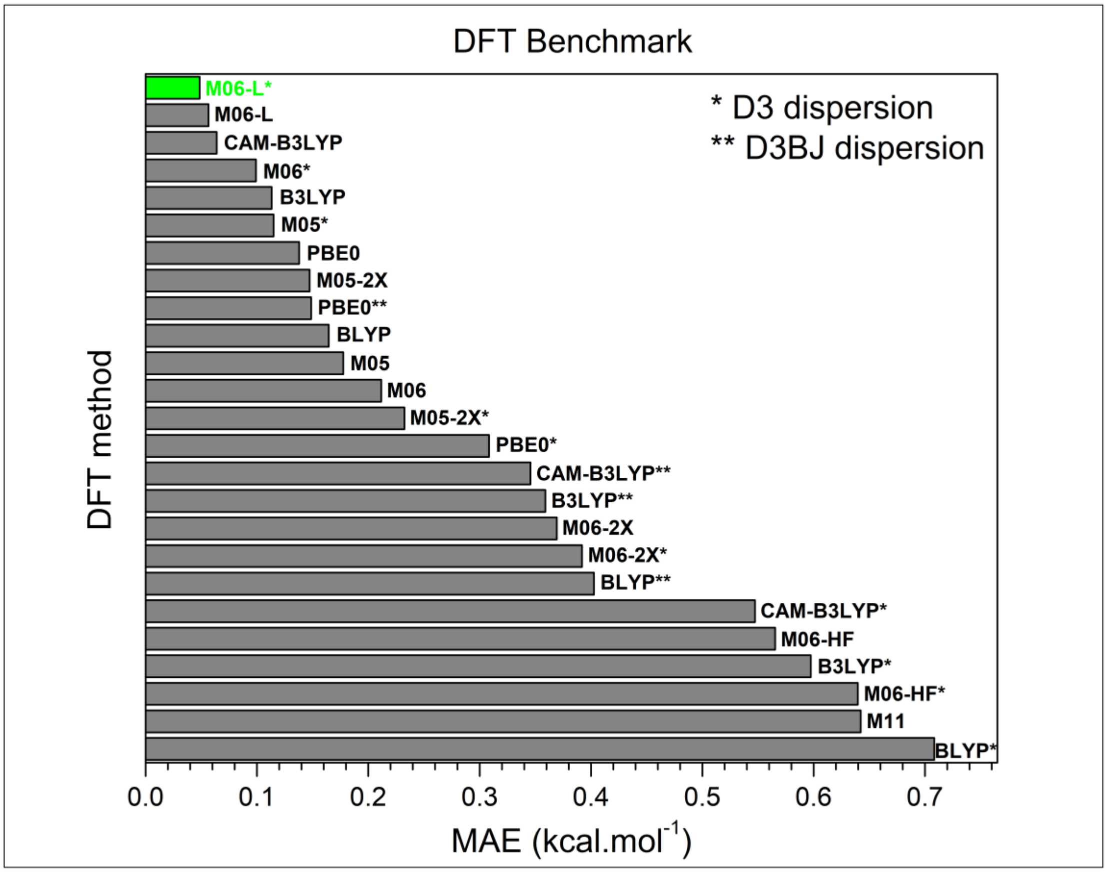

Figure S1. Mean Absolute Errors (MAE) of the single point energies (with thermal correction to Gibbs Free Energy obtained at M06L-D3/aug-cc-pVTZ theoretical level) obtained in different DFT methods using aug-cc-pVTZ basis set with respect to the energy obtained at the DLPNO-CCSD(T)/def2-TZVP level. Highlighted in green is the functional with lowest MAE (0.05 kcal mol^{-1}).

Table S1. NBO analysis results for compounds **3** and **4** calculated at the M06L-D3/aug-cc-pVTZ theoretical level. **Δ*E*(T)** is the total electronic, **Δ*E*(L)** the Lewis, **Δ*E*(NL)** the non-Lewis, **Δ*E*(NCE)** the electrostatic and **Δ*E*(NSA)** the steric energies (in kcal mol^{-1}). Negative values represent a preference of the Me (or Et) group for the axial conformer, and positive ones for the equatorial conformer.

	**Δ*G***	**Δ*E*(T)**	**Δ*E*(L)**	**Δ*E*(NL)**	**Δ*E*(NCE)**	**Δ*E*(NSA)**
**3**	0.76	0.22	-0.95	1.17	-12.03	1.57
**4**	1.63	1.51	0.68	0.82	-8.46	1.13

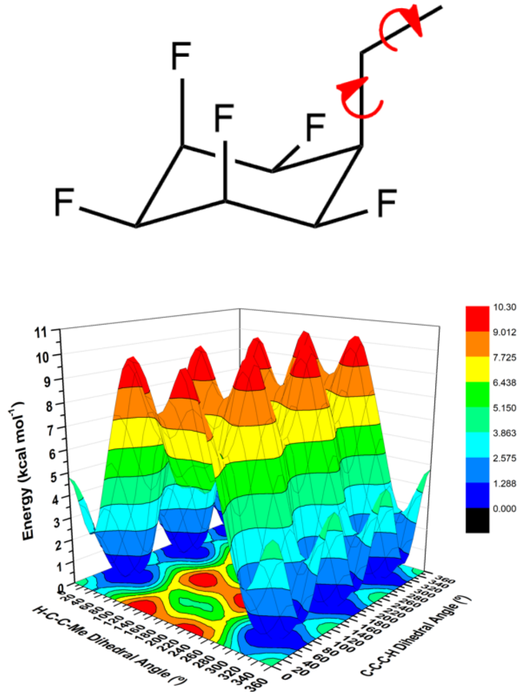

4Et-ax

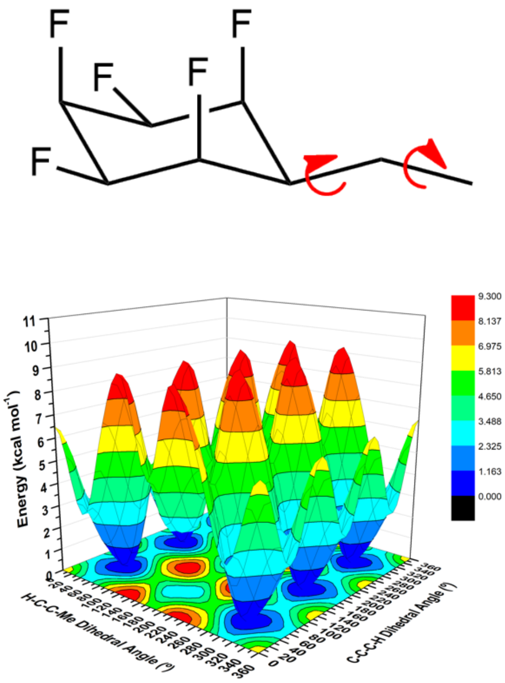

4Et-eq

Figure S2. Potential energy surface (PES) for the rotation associated with the ethyl group (dihedrals are highlighted) of **4** from 0° to 360° in steps of 10° calculated at the M06L-D3/6-311++G(d,p) theoretical level.

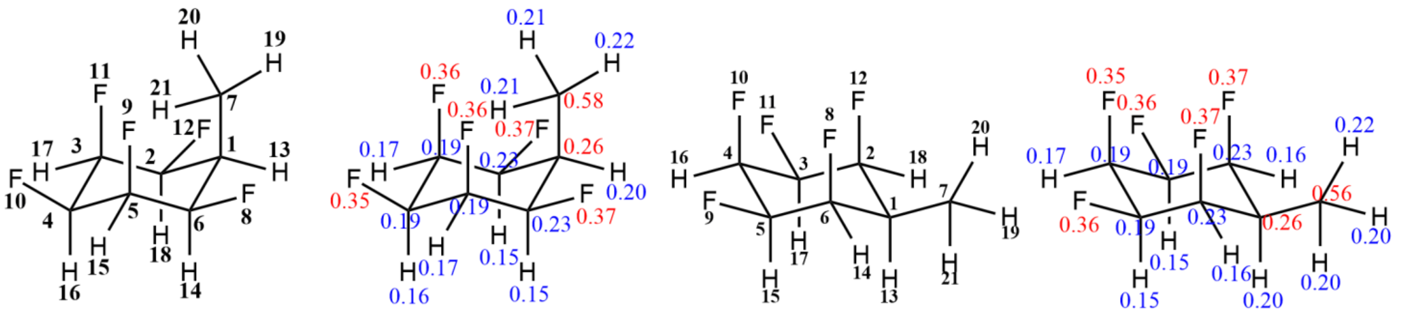

Table S2. Atom-atom electrostatic interactions (kcal mol^{-1}) obtained at M06L-D3/aug-cc-pVTZ theoretical level using NPA charges for **3**. Negative values represent a stabilization for the axial position of the methyl group and positive values stabilization for the equatorial position.

	**1**	**2**	**3**	**4**	**5**	**6**	**7**	**8**	**9**	**10**	**11**	**12**	**13**	**14**	**15**	**16**	**17**	**18**	**19**	**20**
**1**	-	-	-	-	-	-	-	-	-	-	-	-	-	-	-	-	-	-	-	-
**2**	-0.07	-	-	-	-	-	-	-	-	-	-	-	-	-	-	-	-	-	-	-
**3**	0.44	-0.08	-	-	-	-	-	-	-	-	-	-	-	-	-	-	-	-	-	-
**4**	0.08	0.28	-0.25	-	-	-	-	-	-	-	-	-	-	-	-	-	-	-	-	-
**5**	0.44	-0.03	-0.43	-0.25	-	-	-	-	-	-	-	-	-	-	-	-	-	-	-	-
**6**	-0.07	0.57	-0.03	0.28	-0.08	-	-	-	-	-	-	-	-	-	-	-	-	-	-	-
**7**	0.38	-0.64	-1.83	-1.48	-1.83	-0.64	-	-	-	-	-	-	-	-	-	-	-	-	-	-
**8**	-0.17	1.39	1.13	1.42	0.23	-0.56	0.66	-	-	-	-	-	-	-	-	-	-	-	-	-
**9**	1.84	-1.44	-1.25	-0.02	0.41	-0.3	9.54	0.05	-	-	-	-	-	-	-	-	-	-	-	-
**10**	-1.44	1.44	0.17	-0.14	0.17	1.44	0.13	-6.2	0.33	-	-	-	-	-	-	-	-	-	-	-
**11**	1.84	-0.3	0.41	-0.02	-1.25	-1.44	9.54	0.17	6.54	0.33	-	-	-	-	-	-	-	-	-	-
**12**	-0.17	-0.56	0.23	1.42	1.13	1.39	0.66	-6.02	0.17	-6.2	0.05	-	-	-	-	-	-	-	-	-
**13**	-0.11	0.33	-0.97	-0.44	-0.97	0.33	-1.11	-1.95	-0.15	0.86	-0.15	-1.95	-	-	-	-	-	-	-	-
**14**	0.48	0.80	0.20	0.58	-0.37	-0.43	2.37	0.57	1.73	0.22	-0.61	0.2	-0.08	-	-	-	-	-	-	-
**15**	0.72	-0.19	-0.63	0.39	0.62	0.59	-0.83	-2.16	-1.02	-2.06	-0.44	0.47	-1.31	0.10	-	-	-	-	-	-
**16**	-0.38	0.87	-0.36	-0.57	-0.36	0.87	-1.02	0.24	1.79	0.50	1.79	0.24	0.00	1.08	0.14	-	-	-	-	-
**17**	0.72	0.59	0.62	0.39	-0.63	-0.19	-0.83	0.47	-0.44	-2.06	-1.02	-2.16	-1.31	0.05	-0.99	0.14	-	-	-	-
**18**	0.48	-0.43	-0.37	0.58	0.20	0.80	2.37	0.20	-0.61	0.22	1.73	0.57	-0.08	0.99	0.05	1.08	0.10	-	-	-
**19**	-0.26	0.27	0.34	0.37	0.51	0.27	-2.73	-2.20	-2.85	-0.1	-2.19	-0.31	0.46	-1.31	0.30	0.27	0.13	-0.38	-	-
**20**	-0.26	0.27	0.51	0.37	0.34	0.27	-2.73	-0.31	-2.19	-0.1	-2.85	-2.2	0.46	-0.38	0.13	0.27	0.30	-1.31	0.68	-
**21**	0.22	0.01	1.19	0.89	1.19	0.01	-1.61	2.07	-5.96	-0.39	-5.96	2.07	0.13	-0.81	0.72	0.47	0.72	-0.81	0.43	0.43

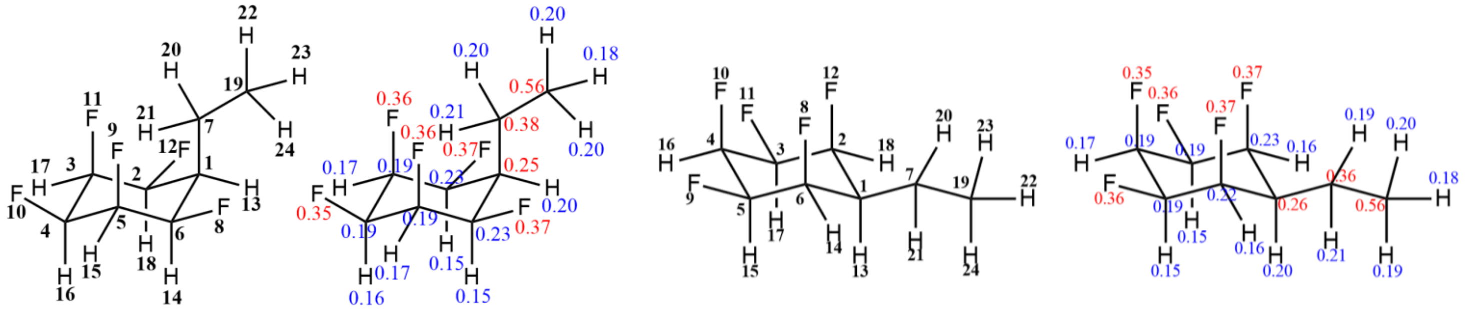

Table S3. Atom-atom electrostatic interactions (kcal mol^{-1}) obtained at M06L-D3/aug-cc-pVTZ theoretical level using NPA charges for **4**. Negative values represent a stabilization for the axial position of the ethyl group and positive values stabilization for the equatorial position.

	1	2	3	4	5	6	7	8	9	10	11	12	13	14	15	16	17	18	19	20	21	22	23
**1**	-	-	-	-	-	-	-	-	-	-	-	-	-	-	-	-	-	-	-	-	-	-	-
**2**	-0.63	-	-	-	-	-	-	-	-	-	-	-	-	-	-	-	-	-	-	-	-	-	-
**3**	0.41	0.13	-	-	-	-	-	-	-	-	-	-	-	-	-	-	-	-	-	-	-	-	-
**4**	0.01	0.5	-0.34	-	-	-	-	-	-	-	-	-	-	-	-	-	-	-	-	-	-	-	-
**5**	0.35	0.16	-0.47	-0.22	-	-	-	-	-	-	-	-	-	-	-	-	-	-	-	-	-	-	-
**6**	-0.18	0.81	-0.09	0.26	-0.10	-	-	-	-	-	-	-	-	-	-	-	-	-	-	-	-	-	-
**7**	0.81	-1.02	-1.28	-1.05	-1.23	-0.43	-	-	-	-	-	-	-	-	-	-	-	-	-	-	-	-	-
**8**	-0.05	1.08	1.20	1.40	0.17	-0.55	0.39	-	-	-	-	-	-	-	-	-	-	-	-	-	-	-	-
**9**	1.84	-1.60	-1.13	-0.03	0.40	-0.23	6.02	0.06	-	-	-	-	-	-	-	-	-	-	-	-	-	-	-
**10**	-1.35	1.17	0.30	-0.14	0.15	1.50	0.22	-6.24	0.42	-	-	-	-	-	-	-	-	-	-	-	-	-	-
**11**	2.07	-0.69	0.64	-0.03	-1.30	-1.50	6.74	0.27	6.52	0.25	-	-	-	-	-	-	-	-	-	-	-	-	-
**12**	-0.09	-1.15	0.37	1.40	1.14	1.41	0.63	-6.00	0.04	-6.23	-0.15	-	-	-	-	-	-	-	-	-	-	-	-
**13**	-0.33	0.58	-0.99	-0.42	-0.95	0.33	-0.90	-1.92	-0.12	0.85	-0.22	-1.97	-	-	-	-	-	-	-	-	-	-	-
**14**	0.44	0.92	0.15	0.54	-0.4	-0.57	1.54	0.64	1.79	0.28	-0.6	0.21	-0.11	-	-	-	-	-	-	-	-	-	-
**15**	0.66	-0.08	-0.69	0.38	0.62	0.56	-0.56	-2.18	-0.98	-2.01	-0.42	0.49	-1.29	0.08	-	-	-	-	-	-	-	-	-
**16**	-0.41	1.01	-0.42	-0.54	-0.35	0.82	-0.72	0.25	1.79	0.50	1.79	0.21	0.00	1.02	0.14	-	-	-	-	-	-	-	-
**17**	0.62	0.8	0.58	0.42	-0.62	-0.19	-0.71	0.44	-0.46	-2.11	-1.06	-2.24	-1.27	0.04	-0.99	0.16	-	-	-	-	-	-	-
**18**	0.54	-0.35	-0.56	0.55	0.18	0.76	1.51	0.27	-0.52	0.31	1.93	0.83	-0.2	0.97	0.02	1.07	-0.01	-	-	-	-	-	-
**19**	0.40	0.50	0.55	0.04	-0.17	-1.32	-0.10	4.02	-1.27	0.93	-3.57	-0.37	0.65	-1.21	-0.09	0.06	0.53	-0.73	-	-	-	-	-
**20**	-0.83	1.51	1.57	1.37	1.60	0.7	-4.08	-1.20	-6.57	-1.66	-6.16	-1.5	-0.26	-1.21	0.84	0.77	0.90	-0.02	0.65	-	-	-	-
**21**	-0.24	2.42	0.78	-0.21	-0.97	-1.93	1.77	6.33	5.81	-0.85	2.36	-3.53	-1.20	0.58	-0.59	-0.18	0.64	5.07	0.89	-1.99	-	-	-
**22**	0.21	-0.42	-0.11	0.08	0.23	0.22	-0.15	-0.99	-1.74	0.20	-1.03	0.79	0.07	-0.39	0.17	0.02	-0.06	-1.14	-0.31	0.38	1.64	-	-
**23**	0.17	-1.57	-0.74	-0.28	-0.04	0.34	0.08	-1.15	-0.92	0.93	0.49	2.54	1.19	-0.09	-0.01	-0.15	-0.47	-2.61	-0.60	0.27	2.31	-0.73	-
**24**	-0.34	-0.47	-0.15	0.34	0.79	1.63	-1.49	-5.56	-2.85	-0.32	-0.84	0.48	-0.01	0.26	0.54	0.24	-0.24	-1.47	0.19	1.14	-2.68	0.37	0.19

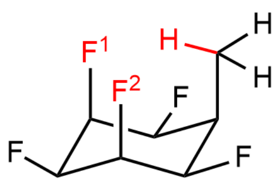

$\mathbf{3}_{\text{Me-ax}}$

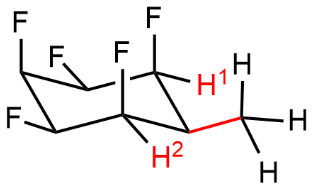

$\mathbf{3}_{\text{Me-eq}}$

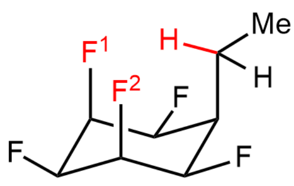

$\mathbf{4}_{\text{Me-ax}}$

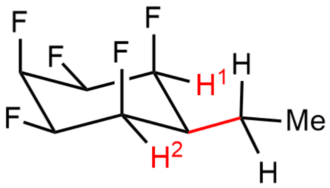

$\mathbf{4}_{\text{Me-eq}}$

Table S4. Selected NLMOs steric repulsions that destabilize the alkyl groups in **3** and **4** in the axial orientation, calculated at the M06L-D3/aug-cc-pVTZ theoretical level. Energies given are in kcal mol^{-1}.

	**Me-axial**		**Me-equatorial**			**Et-axial**		**Et-equatorial**	
	**LP(1) F^1 / σ_{C-H}**	0.84				**LP(1) F^1 / σ_{C-H}**	0.51		
	**LP(2) F^1 / σ_{C-H}**	1.00	**σ_{C-H1} / σ_{C-C}**	1.20		**LP(2) F^1 / σ_{C-H}**	0.59	**σ_{C-H1} / σ_{C-C}**	0.93
**3**	**LP(3) F^1 / σ_{C-H}**	0.51			**4**				
	**LP(1) F^2 / σ_{C-H}**	0.84				**LP(1) F^2 / σ_{C-H}**	0.99		
	**LP(2) F^2 / σ_{C-H}**	1.00	**σ_{C-H2} / σ_{C-C}**	1.20		**LP(2) F^2 / σ_{C-H}**	0.69	**σ_{C-H2} / σ_{C-C}**	1.18
	**LP(3) F^2 / σ_{C-H}**	0.51				**LP(3) F^2 / σ_{C-H}**	1.28		

Table S5 Contribution of selected bonds and lone pairs in compounds **1**, **3** and **4** for the molecular dipole z component (in Debye) calculated at the M06L-D3/aug-cc-pVTZ theoretical level.

**Compound**	**1**	$\mathbf{3}_{\text{Me-ax}}$	$\mathbf{3}_{\text{Me-eq}}$	$\mathbf{4}_{\text{Et-ax}}$	$\mathbf{4}_{\text{Et-eq}}$
**C-F$_{ax}$**	1.84	1.89	1.88	1.86	1.87
**F$_{ax}$ LPs^a**	1.33	1.41	1.39	1.38	1.40
**C-F$_{eq}$^{c}**	0.54	0.55	0.55	0.52	0.48
**F$_{eq}$ LPs^a**	0.44	0.44	0.44	0.45	0.39
**C-R$_{ax}$^{b}**	-	-0.17	-	-0.16	-
**C-R$_{eq}$^{b}**	-	-	-0.08	-	-0.09
**C-H$_{ax}$^{c}**	-1.69	-1.71	-1.69	-1.65	-1.67
**C-H$_{eq}$^{c}**	-0.74	-0.79	-0.77	-0.75	-0.76
**Molecular Dipole (z)**	5.49	3.85	5.13	3.72	4.86
**Molecular Dipole**	5.49	3.87	5.49	3.74	5.47

[a] Dipoles given are the sum of the contribution of the 3 fluorine lone pairs; [b] R = Et or Me; [c] Dipoles given are the average of each bond individual contribution.

Table S6. Cartesian coordinates, energies and Lowest harmonic vibrational frequencies of the optimized geometries for compounds **1**, **3** and **4** obtained at the M06L-D3/aug-cc-pVTZ level in the gas-phase.

1
Energy (hartrees) = -831.448621487
No negative frequencies
Lowest harmonic vibrational frequency (cm^{-1}) = 107.52

C	1.42428200	-0.12062100	-0.68587300
C	0.83881900	1.20452200	-0.22239800
C	-0.60733200	1.29370700	-0.68559700
C	-1.46265400	0.12395100	-0.22291900
C	-0.81681500	-1.17298800	-0.68579000
C	0.62393800	-1.32844000	-0.22306300
H	1.41328200	2.03023800	-0.65190400
H	1.42496500	-0.12010600	-1.78401800
H	-2.46470700	0.20883100	-0.65305000
H	-0.81747600	-1.17379100	-1.78394700
H	1.05170700	-2.23880400	-0.65270500
H	-0.60751300	1.29409900	-1.78373300
F	0.68194000	-1.45049900	1.13887300
F	0.91612900	1.31563500	1.13953700
F	-1.59829200	0.13496000	1.13891400
F	2.72275800	-0.23084900	-0.26276600
F	-1.56096100	-2.24258600	-0.26225600
F	-1.16176200	2.47320000	-0.26305800

3$_{Me-ax}$
Energy (hartrees) = -771.52281474
No negative frequencies
Lowest harmonic vibrational frequency (cm^{-1}) = 107.17

C	1.45865800	0.00006800	-0.30410100
C	-0.76510200	-1.26804000	-0.21239900
C	-1.48416500	-0.00007500	-0.63861700
C	-0.76522800	1.26796600	-0.21241000
C	0.66039000	1.22826900	-0.73678500
C	0.66050700	-1.22820800	-0.73678500
C	1.87181100	0.00008800	1.16016900
F	1.31500200	-2.37985500	-0.35507700
F	-0.77767900	-1.38853300	1.15786600
F	-2.76833300	-0.00013600	-0.15566400
F	-0.77783100	1.38847300	1.15785500
F	1.31477200	2.37997800	-0.35507300
H	2.37053300	0.00011200	-0.90679500
H	0.59507500	-1.24468800	-1.83322000
H	-1.28537900	-2.14143000	-0.61499800
H	-1.53527900	-0.00008300	-1.73505000
H	-1.28558700	2.14130100	-0.61502200
H	0.59496100	1.24474400	-1.83322000
H	2.47365900	-0.88071600	1.36970400
H	2.47360800	0.88092900	1.36969400
H	1.02380500	0.00006700	1.83530500

3$_{Me-ax}$
Energy (hartrees) = -771.52281474
No negative frequencies
Lowest harmonic vibrational frequency (cm^{-1}) = 107.17

C	1.45865800	0.00006800	-0.30410100
C	-0.76510200	-1.26804000	-0.21239900
C	-1.48416500	-0.00007500	-0.63861700
C	-0.76522800	1.26796600	-0.21241000
C	0.66039000	1.22826900	-0.73678500
C	0.66050700	-1.22820800	-0.73678500
C	1.87181100	0.00008800	1.16016900
F	1.31500200	-2.37985500	-0.35507700
F	-0.77767900	-1.38853300	1.15786600
F	-2.76833300	-0.00013600	-0.15566400
F	-0.77783100	1.38847300	1.15785500
F	1.31477200	2.37997800	-0.35507300
H	2.37053300	0.00011200	-0.90679500
H	0.59507500	-1.24468800	-1.83322000
H	-1.28537900	-2.14143000	-0.61499800
H	-1.53527900	-0.00008300	-1.73505000
H	-1.28558700	2.14130100	-0.61502200
H	0.59496100	1.24474400	-1.83322000
H	2.47365900	-0.88071600	1.36970400
H	2.47360800	0.88092900	1.36969400
H	1.02380500	0.00006700	1.83530500

4$_{Et-ax}$
Energy (hartrees) = -810.841679449
No negative frequencies
Lowest harmonic vibrational frequency (cm^{-1}) = 48.35

4$_{Et-ax}$
Energy (hartrees) = -810.841679449
No negative frequencies
Lowest harmonic vibrational frequency (cm^{-1}) = 48.35

C	1.07474000	0.33320100	-0.60526000	C	1.07474000	0.33320100	-0.60526000
C	-0.03344200	1.35901900	-0.84503700	C	-0.03344200	1.35901900	-0.84503700
C	-1.30335700	1.08992000	-0.05939300	C	-1.30335700	1.08992000	-0.05939300
C	-1.82477600	-0.28542900	-0.43386800	C	-1.82477600	-0.28542900	-0.43386800
C	-0.79862300	-1.38604100	-0.22820100	C	-0.79862300	-1.38604100	-0.22820100
C	0.48149500	-1.02779000	-0.96800900	C	0.48149500	-1.02779000	-0.96800900
C	1.73290900	0.43265100	0.77657600	C	1.73290900	0.43265100	0.77657600
F	1.40167900	-2.03575700	-0.76911700	F	1.40167900	-2.03575700	-0.76911700
F	-0.55897000	-1.56809500	1.11415200	F	-0.55897000	-1.56809500	1.11415200
F	-2.96684700	-0.56970800	0.27085600	F	-2.96684700	-0.56970800	0.27085600
F	-1.05521100	1.15720600	1.29244100	F	-1.05521100	1.15720600	1.29244100
F	0.41858000	2.62952400	-0.55825300	F	0.41858000	2.62952400	-0.55825300
H	1.84691600	0.54396700	-1.35164300	H	1.84691600	0.54396700	-1.35164300
H	0.23258700	-1.02939800	-2.03842900	H	0.23258700	-1.02939800	-2.03842900
H	-1.18963500	-2.33255500	-0.61150100	H	-1.18963500	-2.33255500	-0.61150100
H	-2.08180200	-0.26365300	-1.50062600	H	-2.08180200	-0.26365300	-1.50062600
H	-2.05597000	1.84914500	-0.28819400	H	-2.05597000	1.84914500	-0.28819400
H	-0.30607200	1.35115900	-1.90895500	H	-0.30607200	1.35115900	-1.90895500
H	1.72687900	1.48379800	1.06480300	H	1.72687900	1.48379800	1.06480300
H	1.13313700	-0.08566100	1.51993500	H	1.13313700	-0.08566100	1.51993500
C	3.16035800	-0.08276700	0.79160900	C	3.16035800	-0.08276700	0.79160900
H	3.61348800	0.06693800	1.76981000	H	3.61348800	0.06693800	1.76981000
H	3.77751300	0.44498100	0.06291500	H	3.77751300	0.44498100	0.06291500
H	3.21405500	-1.14383600	0.56067900	H	3.21405500	-1.14383600	0.56067900

References

1. M. J. Frisch, G. W. Trucks, H. B. Schlegel, G. E. Scuseria, M. A. Robb, J. R. Cheeseman, G. Scalmani, V. Barone, G. A. Petersson, H. Nakatsuji, X. Li, M. Caricato, A. V. Marenich, J. Bloino, B. G. Janesko, R. Gomperts, B. Mennucci, H. P. Hratchian, J. V. Ortiz, A. F. Izmaylov, J. L. Sonnenberg, D. Williams-Young, F. Ding, F. Lipparini, F. Egidi, J. Goings, B. Peng, A. Petrone, T. Henderson, D. Ranasinghe, V. G. Zakrzewski, J. Gao, N. Rega, G. Zheng, W. Liang, M. Hada, M. Ehara, K. Toyota, R. Fukuda, J. Hasegawa, M. Ishida, T. Nakajima, Y. Honda, O. Kitao, H. Nakai, T. Vreven, K. Throssell, J. A. Montgomery, Jr., J. E. Peralta, F. Ogliaro, M. J. Bearpark, J. J. Heyd, E. N. Brothers, K. N. Kudin, V. N. Staroverov, T. A. Keith, R. Kobayashi, J. Normand, K. Raghavachari, A. P. Rendell, J. C. Burant, S. S. Iyengar, J. Tomasi, M. Cossi, J. M. Millam, M. Klene, C. Adamo, R. Cammi, J. W. Ochterski, R. L. Martin, K. Morokuma, O. Farkas, J. B. Foresman, and D. J. Fox, Gaussian 16 (Revision C.01), Gaussian, Inc., Wallingford CT, 2016.
2. E. D. Glendening, J, K. Badenhoop, A. E. Reed, J. E. Carpenter, J. A. Bohmann, C. M. Morales, P. Karafiloglou, C. R. Landis, and F. Weinhold, NBO 7.0., Theoretical Chemistry Institute, University of Wisconsin, Madison, 2018

Langmuir Isotherm analysis set up

Langmuir isotherms were carried out using a Biolin Scientific KSV Nima KN-2002 (“medium”) Langmuir Trough with PC interface (for displaying real time surface pressures and barrier positions) and subphase temperature control. The resolution for surface tension measurement was 0.03 µN/m, with a measuring range up to 300 mN/m. The subphase temperature was regulated with a Grant GR150 water circulator and monitored using a digital probe.

Cleaning

Troughs and barriers were cleaned using an Alconox solution (10g/L), which was rubbed over the PTFE surface in circular motions with surfactant-free Kimwipes. The components were subsequently rinsed with DI water followed by blow drying with nitrogen in five consecutive cycles.

Calibration

The Wilhelmy probe was calibrated following the manufacturer’s guidelines, with a precisely measured washer (273.2 mg) that served as calibration weight. Measurements of the surface pressure were conducted using Wilhelmy plates made of paper, which were soaked in DI water for 15 min prior to each experiment. A surface tension measurement of the subphase (DI water) was performed to validate the geometrical features of the Wilhelmy plate; by zeroing the Wilhelmy balance whilst the water-saturated Wilhelmy plate was suspended in mid-air, then touching the plate to the water surface and ensuring a reading of 72.8 ± 0.1 mN/m was recorded. Movement of the barriers was restricted by physical limit switches placed at the “fully open” and “fully closed” positions. These were located at barrier positions 0 mm and 130 mm, respectively. Please note that the open area was 23,650 mm^2 and the closed was 2,410 mm^2, leading to a maximum compression ratio of ~10.

Filling and surface cleaning

In order to fill the trough with the subphase, the barriers were fully closed at barrier position of 130 mm. DI water was added from outside the barrier-delimited surface until the water level exceeded the barrier-trough contact level by 2-5 mm (a meniscus formed due to the hydrophobicity of the PTFE on the surface of the trough). In order to assess cleanliness of the water surface, the balance was set to 0 mN/m in the fully open position, and a full-compression isotherm measurement was performed at constant compression speed (typically 65 mm/min). A surface pressure of increase of <0.3 mN/m was set as the acceptance criteria for the subphase surface to be considered as “clean”. Otherwise, surface-borne contaminants were removed using a pipet tip that was attached to a negative pressure aspirator and liquid trap (or further still, emptying the subphase and returning to the cleaning procedure).

Deposition:

Once the set-up was calibrated and assessed as “clean”, samples were deposited either dropwise/manually from a microsyringe over the course of 30 min, or by continuous micro-propulsive injection over a similar time interval (using a syringe pump attached to a hypodermic needle at the air liquid interface). An equilibration period of 30 min was allowed post-spreading. Thereafter, the compression was carried out a constant rate relative to remaining area of 5% per minute. See Table S6 for further details:

Table S6 Experimental details for Langmuir Blodgett experiments.

**Compound**	**7**	**8**	**21**	**22**
**Solvent**	$(CH_3)_2CO$	$CHCl_3$	$CHCl_3$	$CHCl_3$
**Concentration [mg/ml]**	0.65	1.25	0.32	0.89
**Total volume [ul]**	100	20	100	20
**Type of deposition**	syringe pump	manual	manual	manual

X-ray Crystallography Details

X-ray diffraction data for compounds **3** and **8** were collected at 125 K using a Rigaku MM-007HF High Brilliance RA generator/confocal optics with XtaLAB P200 diffractometer [Cu Kα radiation (λ = 1.54187 Å)]. Diffraction data for compound **4** were collected at 93 K using a Rigaku FR-X Ultrahigh Brilliance Microfocus RA generator/confocal optics with XtaLAB P200 diffractometer [Mo Kα radiation (λ = 0.71075 Å)]. Diffraction data for compounds **5**, **6** and **9** were collected at 173 K using a Rigaku MM-007HF High Brilliance RA generator/confocal optics with XtaLAB P100 diffractometer [Cu Kα radiation (λ = 1.54187 Å)]. In all cases, intensity data were collected using either just ω steps or both ω and φ steps accumulating area detector images spanning at least a hemisphere of reciprocal space. Data for all compounds analysed were collected using CrystalClear.[1] and processed (including correction for Lorentz, polarization and absorption) using either CrystalClear or CrysAlisPro.[2] Structures were solved by dual-space (SHELXT[3]) or direct (SIR2004[4]) methods, and refined by full-matrix least-squares against F^2 (SHELXL-2018/3[5]). Non-hydrogen atoms were refined anisotropically, and hydrogen atoms were refined using a riding model, except for the OH hydrogen in **8** which was located from the difference Fourier map and refined isotropically subject to a distance restraint. Compounds **5**, **6** and **8** all diffracted weakly at high angles, resulting in elevated values of R_{int}, and correspondingly higher values of R_1 and wR_2. Compounds **6** and **8** were refined as two-component non-merohedric twins, however the continuing elevated values of R_1 and wR_2 suggest further twinning or polycrystallinity could be present, although all attempts to incorporate this into the refinement were unsuccessful. Despite the somewhat lower data-quality for compounds **5**, **6** and **8**, all three structures could be determined unambiguously. All calculations were performed using either the CrystalStructure[6] or Olex2[7] interface. Selected crystallographic data are presented in Table S7. Deposition numbers 2068702-2068707 contains the supplementary crystallographic data for this paper. These data are provided free of charge by the joint Cambridge Crystallographic Data Centre and Fachinformationszentrum Karlsruhe Access Structures service www.ccdc.cam.ac.uk/structures.

References

1. CrystalClear-SM Expert v2.1., Rigaku Americas, The Woodlands, Texas, USA, and Rigaku Corporation, Tokyo, Japan, 2015.
2. CrysAlisPro v1.171.39.8d or v1.171.40.14a. Rigaku Oxford Diffraction, Rigaku Corporation, Oxford, U.K., 2015-2018.
3. G. M. Sheldrick, *Acta Crystallogr., Sect. A*. 2015, **71**, 3-8.
4. M. C. Burla, R. Caliandro, M. Camalli, B. Carrozzini, G. L. Cascarano, L. De Caro, C. Giacovazzo, G. Polidori and R. Spagna, *J. Appl. Crystallogr*. 2005, **38**, 381-388.
5. G. M. Sheldrick, *Acta Crystallogr., Sect. C*. 2015, **71**, 3-8.
6. CrystalStructure v4.3.0. Rigaku Americas, The Woodlands, Texas, USA, and Rigaku Corporation, Tokyo, Japan, 2018.
7. O. V. Dolomanov, L. J. Bourhis, R. J. Gildea, J. A. K. Howard and H. Puschmann, *J. Appl. Crystallogr*. 2009, **42**, 339-341.

Table S7. Selected crystallographic data.

	**3**	**4**	**5**	**6**	**8**	**9**
CCDC number	2068702	2068703	2068704	2068705	2068706	2068707
empirical formula	$C_7H_9F_5$	$C_8H_{11}F_5$	$C_{14}H_{16}F_{10}$	$C_{24}H_{36}F_{10}$	$C_{19}H_{33}F_5O$	$C_{19}H_{33}F_5$
fw	188.14	202.17	374.27	514.53	372.46	356.46
crystal description	Colourless prism	Colourless plate	Colourless plate	Colourless plate	Colourless plate	Colourless plate
crystal size [mm^3]	0.10×0.10×0.10	0.10×0.10×0.01	0.05×0.03×0.01	0.05×0.05×0.01	0.03×0.02×0.01	0.33×0.12×0.01
Temperature [K]	125	93	173	173	125	173
space group	*C*2/*m*	*Pbca*	*P*2$_1$/*c*	*P*2$_1$/*c*	*P*2$_1$2$_1$2$_1$	P2_1
a [Å]	8.3314(3)	15.594(7)	6.3268(9)	18.982(6)	5.5481(6)	5.64684(18)
b [Å]	7.6652(3)	12.835(7)	11.8350(16)	5.1880(10)	5.5766(6)	5.62268(14)
c [Å]	11.5888(4)	8.557(4)	9.9391(16)	12.315(2)	62.078(8)	30.1035(11)
β [°]	99.667(3)		103.293(16)	97.79(2)		95.117(3)
vol [Å]^3	729.57(5)	1712.7(15)	724.28(19)	1201.6(5)	1920.7(4)	951.99(5)
Z	4	8	2	2	4	2
ρ (calc) [g/cm^3]	1.713	1.568	1.716	1.422	1.288	1.243
μ [mm^{-1}]	1.715	0.166	1.726	1.193	0.943	0.887
F(000)	384	832	380	540	800	384
reflections collected	3739	18234	7216	11100	21334	12752
independent reflections (R_{int})	773 (0.0149)	1575 (0.1006)	1318 (0.1814)	2183 (0.1189)	3910 (0.2214)	3392 (0.0361)
parameters, restraints	63, 0	119, 0	110, 0	155, 0	231, 1	218, 1
GoF on F^2	1.172	0.955	1.091	1.723	1.809	1.091
R_1 [$I > 2\sigma(I)$]	0.0339	0.0515	0.1179	0.1661	0.1806	0.0427
wR_2 (all data)	0.0955	0.1381	0.3811	0.5161	0.4893	0.1233
largest diff. peak/hole [e/Å^3]	0.29, -0.23	0.41, -0.22	0.57, -0.42	0.67, -0.56	0.67, -0.57	0.25, -0.20
